# Discovery of a New Class of Lipophilic Pyrimidine-Biphenyl Herbicides Using an Integrated Experimental-Computational Approach

**DOI:** 10.3390/molecules29112409

**Published:** 2024-05-21

**Authors:** Yitao Yan, Yinglu Chen, Hanxian Hu, Youwei Jiang, Zhengzhong Kang, Jun Wu

**Affiliations:** 1 Department of Chemistry, Zhejiang University, Hangzhou 310058, China; 2 School of Physics, Zhejiang University, Hangzhou 310027, China; 3 Hangzhou Jingyinkang Biological Technology Co., Ltd., Hangzhou 311110, China; 4 Beijing Life Science Academy, Beijing 102200, China; kangzhzh2007@126.com

**Keywords:** acetohydroxyacid synthase, herbicide, biphenyl, hydrophobic interaction, molecular docking, molecular dynamics simulation

## Abstract

Herbicides are useful tools for managing weeds and promoting food production and sustainable agriculture. In this study, we report on the development of a novel class of lipophilic pyrimidine-biphenyl (PMB) herbicides. Firstly, three PMBs, **Ia**, **IIa**, and **IIIa**, were rationally designed via a scaffold hopping strategy and were determined to inhibit acetohydroxyacid synthase (AHAS). Computational simulation was carried out to investigate the molecular basis for the efficiency of PMBs against AHAS. With a rational binding mode, and the highest in vitro as well as in vivo potency, **Ia** was identified as a preferable hit. Furthermore, these integrated analyses guided the design of eighteen new PMBs, which were synthesized via a one-step Suzuki–Miyaura cross-coupling reaction. These new PMBs, **Iba-ic**, were more effective in post-emergence control of grass weeds compared with **Ia**. Interestingly, six of the PMBs displayed 98–100% inhibition in the control of grass weeds at 750 g ai/ha. Remarkably, **Ica** exhibited ≥ 80% control against grass weeds at 187.5 g ai/ha. Overall, our comprehensive and systematic investigation revealed that a structurally distinct class of lipophilic PMB herbicides, which pair excellent herbicidal activities with new interactions with AHAS, represent a noteworthy development in the pursuit of sustainable weed control solutions.

## 1. Introduction

Herbicides are useful tools for managing weeds and thus promote food production and sustainable agriculture along with pesticides [1] and fungicides [2]. Acetohydroxyacid synthase (AHAS) is the primary enzyme involved in the biosynthesis of branched-chain amino acids (BCAAs) in plants, bacteria, and fungi but not mammals [3]. AHAS catalyzes the conversion of two molecules of pyruvate or 2-ketobutyrate to form acetolactate or acetohydroxybutyrate, respectively, which are the first intermediate products in the biosynthesis of BCAAs [4]. Inhibition of AHAS by its inhibitors results in the death of plants or microorganisms due to the failure to produce BCAAs. Owing to these features, AHAS has been identified as an effective target for herbicides [5] and fungicides [6] since the 1980s.

As a well-established mode of action, herbicides targeting AHAS offer several advantages for agriculture, including low mammalian toxicity, low application rate, and good crop selectivity [3]. To date, five major classes of AHAS-inhibiting herbicides, including sulfonylureas (SUs), pyrimidinylbenzoates (PYBs), triazolopyrimidines (TPs), sulfonylamino-carbonyl-triazolinones (SCTs), and imidazolinones (IMIs) have been developed and widely used in weed control [7]. Over the past two decades, the crystal structures of plant AHAS [3] and its complexes with herbicides [7,8,9,10,11,12] have been determined by Guddat and coworkers. These studies provided new insights into the inhibitory mechanism of the herbicides, suggesting that in addition to considering the affinity of AHAS to herbicides, the effect of cumulative inhibition should also be considered [7]. In addition, the binding modes of the five classes of herbicides revealed by the crystal structures are divided into two major types, i.e., type-A (SUs, PYBs, TPs, and SCTs) [7] and type-B (IMIs) [9]. For type-A, the herbicides enter the AHAS binding pocket with a heterocyclic ring and rely on π-π stacking interaction and hydrogen bondings [7,9,10], while for type-B, IMIs bind to the entrance of the AHAS pocket and also form hydrogen bondings [9]. These insights are paving the way towards the design of novel AHAS inhibitors and the discovery of novel interactions within this well-studied biochemical system.

On the basis of the comprehensive understanding of the AHAS inhibitory mechanism, significant progress has been achieved in the computer-aided design of novel AHAS inhibitors in recent years. For example, Yang and coworkers [13,14,15,16,17,18,19] have designed several series of novel AHAS inhibitors based on the binding modes of PYBs, exhibiting excellent herbicidal activities and anti-resistance properties. Of particular interest were two classes of biphenyl-containing AHAS inhibitors [14,15]. These inhibitors bearing carboxyl groups were polarized. Key to the binding of these AHAS inhibitors were the hydrogen bondings formed between the oxygen atoms of the carboxyl group and R377, K256’, or S653 and π-π interaction with W574. Esterification of the carboxyl group had a detrimental effect on the herbicidal activity [14]. Apparently, the carboxyl group was one of the key factors for them to maintain their herbicidal activities. As can be seen, designing a new class of carboxyl-free biphenyl herbicides is extremely challenging.

**Figure 1:**
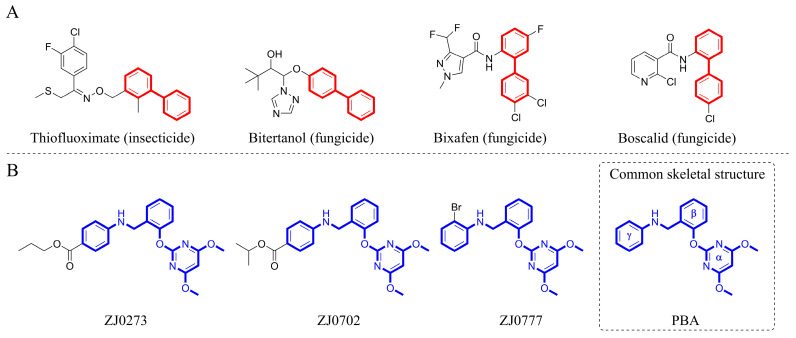
(**A**) Agrochemicals containing biphenyl moiety and (**B**) pyrimidinyloxybenzylamines (PBAs).

Determination of the crystal structure revealed that there is a set of non-polar residues (i.e., V485, M570, V571, M124’, F206’) in the deepest part of the AHAS binding pocket [7], which offers the possibility for the pocket to achieve complementarity with lipophilic inhibitors. Additionally, it has been realized that the interaction of lipophilic moieties of the inhibitors with the receptor is driven by the tendency of non-polar molecules to aggregate and repel water, which is known as the hydrophobic effect [20]. After being buried in the hydrophobic pocket, lipophilic moieties interact with the non-polar residues and displace water molecules, enhancing the affinity of the inhibitors to the target receptor. On the other hand, biphenyls are characterized by strong hydrophobicity, good lipid solubility, cell membrane permeability, and metabolic stability [21,22] and are important molecular skeletons in many agrochemicals (Figure 1A) [23]. Also, biphenyls can be easily synthesized via transition-metal-catalyzed Suzuki–Miyaura cross-coupling reaction, one of the most powerful tools for the construction of the biphenyl core [24]. Based on the above analysis, biphenyl was chosen as a privileged scaffold for novel herbicide discovery in this work. To our knowledge, the design and development of lipophilic AHAS-inhibiting herbicides have not been given adequate attention. Therefore, it is still possible to find new chemistry and novel interactions within this well-studied AHAS system. Herein, we provide a detailed account of our de novo design of lipophilic pyrimidine-biphenyl (PMB), our optimization efforts, and structure–activity relationship (SAR) studies via an integrated experimental-computational approach.

## 2. Results

ZJ0273, ZJ0702, and ZJ0777 (Figure 1B), belonging to pyrimidinyloxybenzylamines (PBAs), are active ingredients in oilseed rape herbicides [25]. This class of herbicides inhibits AHAS and exerts excellent herbicidal activities with the recommended application rate as low as 45–67.5 g ai/ha (gram active ingredient per hectare). The structure of pyrimidinyloxybenzylamines is comprised of a hydrophilic pyrimidine ring and two hydrophobic phenyl rings, which are connected via an oxygen atom and methylene-amine (-CH_2_-NH-) linker. The three aromatic rings are linked by sp^3^ atoms so that the structure shows great flexibility. For these reasons, we started with a structure-based de novo herbicide design from PBA sharing the same skeleton with ZJ0273, ZJ0702, and ZJ0777 (Figure 1B).

**Figure 2:**
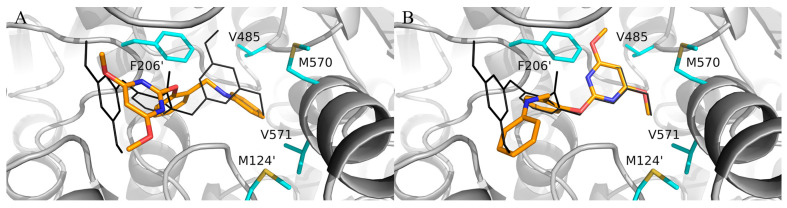
Superposition of bispyribac (black lines) with (**A**) pose-1 and (**B**) pose-2 of PBA (orange sticks) in the AHAS pocket. The non-polar residues surrounding the binding pocket are shown in cyan sticks.

**Figure 3:**
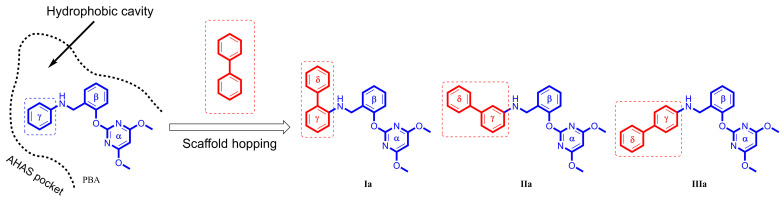
Design of PMBs **Ia**, **IIa**, and **IIIa** from PBA.

In the preliminary docking study, PBA was successfully docked into the binding pocket of AHAS. As shown in Figure 2, PBA could overlap with bispyribac via two possible binding modes (poes-1 and pose-2). Pose-2 was similar to the type-A binding mode (vide supra), while pose-1 was not found in any other AHAS inhibitors. Further analysis of pose-1 revealed that the γ-ring of PBA established hydrophobic interactions with non-polar residues in the pocket, suggesting that the hydrophobic structural fragment could be introduced at the γ-ring of PBA. Therefore, PBA was utilized as a starting point for the design of novel AHAS herbicides based on pose-1. As shown in Figure 2, when PBA entered the hydrophobic cavity via pose-1, we observed that there was still some extra space around the γ-ring of PBA. We hypothesized that replacing the γ-ring of PBA with a more lipophilic biphenyl ring via a scaffold hopping approach would have the potential to greatly enhance the AHAS inhibitory activity since the bulkier biphenyl ring could efficiently fill the extra space and thus enhance the hydrophobic interactions with the non-polar residues in the cavity. Thus, we first designed three lipophilic PMBs, **Ia**, **IIa**, and **IIIa** (Figure 3).

**Ia**, **IIa**, and **IIIa** were synthesized according to the procedure shown in Scheme S1. Aminobiphenyls (**1a**–**c**) reacted with salicylaldehyde (**2**) to form Schiff bases, followed by the reduction with sodium borohydride to yield benzylamines (**3a**–**c**), which in turn reacted with 2-methylsulfonyl-4,6-dimethoxypyrimidine (**4**) in the presence of cesium carbonate to afford PMBs **Ia**, **IIa**, and **IIIa**.

To evaluate the potency of PMBs along with PBA against AHAS, their IC_50_ values were determined. Four compounds had inhibitory activity at micromolar levels and showed the following trend in activity: **Ia** (157 µM) > **IIa** (189 µM) > **IIIa** (343 µM) > PBA (593 µM). The PMBs exhibited more potent AHAS inhibitory activity than that of PBA, indicating that the introduction of the biphenyl core as a good replacer of the γ-ring in PBA was an ideal scaffold hopping strategy.

**Figure 4:**
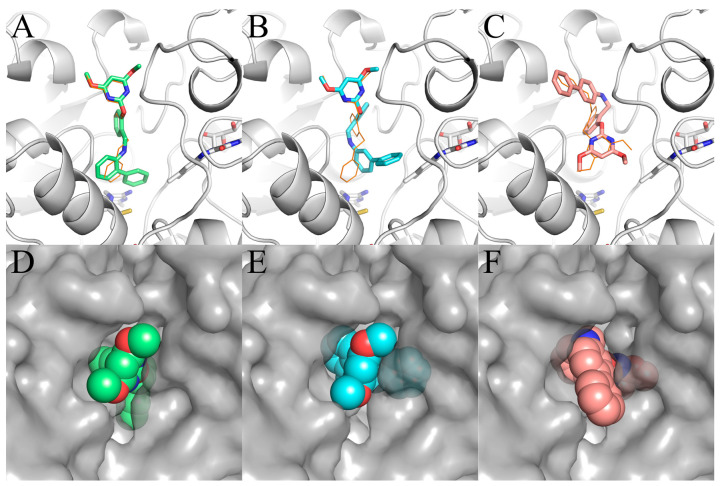
Simulated binding modes of (**A**,**D**) **Ia**, (**B**,**E**) **IIa**, and (**C**,**F**) **IIIa** in AHAS shown in stick model (**A**–**C**) and surface representation (**D**–**E**). FAD and ThAthDP are shown in white sticks. PBA is shown in orange lines.

After validating the AHAS inhibition activity of carboxyl-free PMBs **Ia**, **IIa**, and **IIIa**, we set out to evaluate the inhibition mechanism of three newly discovered AHAS inhibitors in an effort to identify the original hit from these PMBs. To begin with, we performed molecular docking studies on **Ia**, **IIa**, and **IIIa** with AHAS and found that all three hybrids may exert their potency by blocking the substrate access channel in AHAS, like many other AHAS herbicides (Figure 4).

Our docking studies also yielded crucial information concerning the conformation of **Ia**, **IIa**, and **IIIa** (Figure 4). In the binding modes of **Ia** and **IIa**, the biphenyl moieties of **Ia** and **IIa** were found to enter deeply into the binding pocket, whereas their pyrimidine moieties were directed toward the solvent. The binding modes of **Ia** and **IIa** were similar to pose-1 found in PBA. In contrast, for **IIIa**, the pyrimidine moiety entered into the AHAS pocket, and the biphenyl moiety pointed outward, which was comparable to pose-2. The surface representation of the binding of **Ia**, **IIa**, and **IIIa** to AHAS is shown in Figure 4D–F. In pose-1, two oxygen atoms and two nitrogen atoms of pyrimidine moiety in **Ia** and **IIa** faced toward the solvent (Figure 4D,E). Thus, they did provide a favorable polar surface to interact externally with the solvent. In contrast, for pose-2, the non-polar biphenyl moiety faced toward the solvent (Figure 4F), which would be unfavorable for the occurrence of the solvation process.

Interestingly, **Ia** and **IIa** with the binding pose-1 had stronger AHAS inhibitory activity than **IIIa** with binding pose-2. Therefore, this promising binding pose-1 could serve as the active conformation for use in the subsequent virtual screening and structure optimization.

To shed further light on the binding modes of PMBs **Ia**, **IIa**, and **IIIa** with AHAS, and in an effort to assay the stabilities of the binding systems, 50 ns MD simulations were performed. As shown in Figure S1, the root-mean-square deviation (RMSD) values of the AHAS backbone atoms were found to be 0.31, 0.17, and 0.28 nm for ligand–AHAS complexes of compounds **Ia**, **IIa**, and **IIIa**, respectively. The dynamic convergences of the three systems were all achieved after 40 ns of simulations. Hence, MD simulation trajectories in the time range of 40–50 ns can be used to analyze the ligand–AHAS systems.

**Figure 5:**
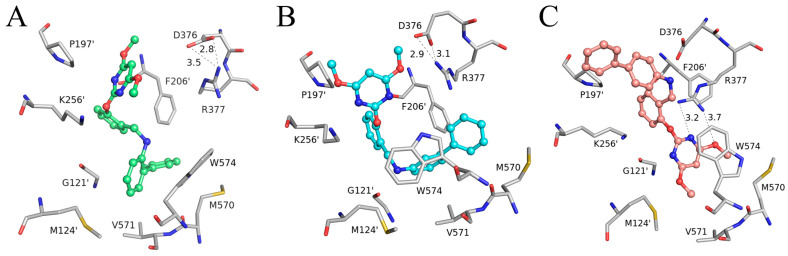
Key residues of AHAS interacting with (**A**) **Ia**, (**B**) **IIa**, and (**C**) **IIIa** at equilibrium of MD simulation. Ligands are shown in the stick and ball model, and residues are shown in the stick model. Hydrogen bonds are indicated with black dotted lines. The distances (Å) labeled in the figure are averaged over a 40–50 ns trajectory.

The specific binding modes of PMBs in AHAS revealed by MD simulations are shown in Figure 5, where snapshots of representative conformations were obtained using a GROMOS-based clustering algorithm [26]. As shown in Figure 5A, the biphenyl moiety of **Ia** established hydrophobic interactions with surrounding residues (M570, V571, W574, G121’, M124’, and F206’). Surprisingly, H-bonding interactions between **Ia** and residues R377 or K256’ were not observed. Moreover, neither the γ-ring nor the δ-ring of **Ia** formed a π-π interaction with W574. This suggested that non-polar interactions played a more important role in the binding of **Ia** to AHAS. As indicated in Figure 5B, **IIa** also formed non-polar contacts with nearby residues (V571, W574, G121’, and M124’). As with **Ia**, **IIa** also failed to form H-bonds with residues R377 or K256’. In contrast, under appropriate geometrical conditions, the imine moiety was able to form a unique N-H∙∙∙π interaction with W574 [27]. As illustrated in Figure 5C, the conformation of **IIIa** changed from V-shaped (Figure 4C) to linear and brought the biphenyl moiety closer to P197’ and K256’, thus enhancing their hydrophobic interactions. The pyrimidine ring of **IIIa** was stabilized by the formation of a bidentate hydrogen bond with R377 and a π-π interaction with W574. Notably, the formation of a bidentate H-bond between **IIIa** and R377 brought R377 closer to **IIIa** and away from D376, resulting in the broken H-bond between R377 and D376. This phenomenon was not observed in either **Ia**–AHAS or **IIa**–AHAS.

To further explore the cause of the inhibitory potency against AHAS, we performed binding free energy analyses of **Ia**, **IIa**, and **IIIa** using the MMPBSA method [28] (Table 1). The binding free energy (Δ*G*_bind_) correlated with the experimental values of **Ia** (IC_50_ = 157 µM), **IIa** (IC_50_ = 189 µM), and **IIIa** (IC_50_ = 343 µM), supporting the reliability of MD simulations. The van der Waals interactions played a more important role than the electrostatic interaction, suggesting that the non-polar interactions in the three systems were more favorable for the binding of ligands **Ia**, **IIa**, and **IIIa** to AHAS. On the other hand, the values of Δ*G*_SOL_ were positive for all three systems, indicating that PMBs needed to overcome desolvation energy barriers to form ligand–AHAS complexes. Therefore, the influence of Δ*G*_SOL_ on Δ*G*_bind_ was not negligible.

**Figure 6:**
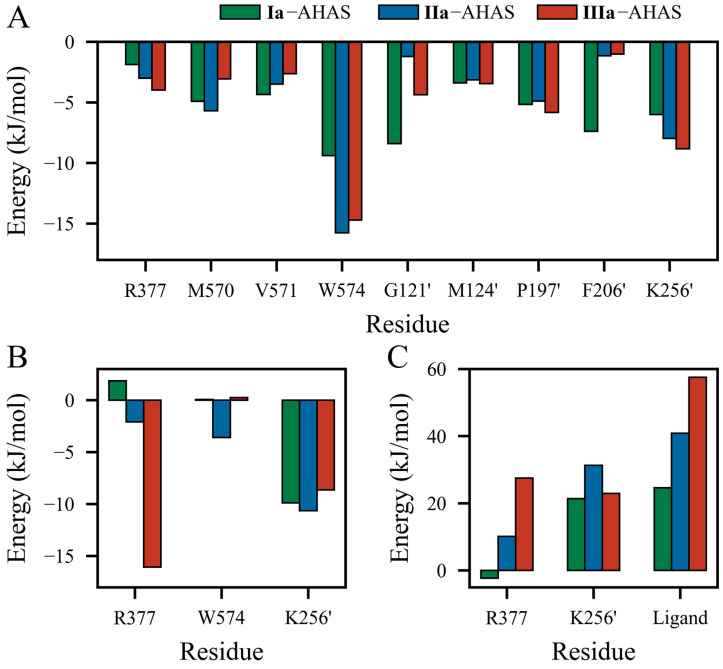
Energy contributions of key residues to the binding of PMBs with AHAS at the equilibrium of the MD simulation. Contributions of residues to total (**A**) van der Waals interaction energy (Δ*E*_VdW_), (**B**) electrostatic interaction energy (Δ*E*_ELE_), and (**C**) solvation energy (Δ*G*_SOL_).

To investigate the detailed contributions of residues in **Ia**–AHAS, **IIa**–AHAS, and **IIIa**–AHAS systems, the energy terms, i.e., Δ*E*_VdW_, Δ*E*_ELE_, and Δ*G*_SOL_ were decomposed into per-residue energy contributions separately. The energy contributions of key residues are shown in Figure 6. As shown in Figure 6A, the Δ*E*_VdW_ contributions of non-polar residues (M570, V571, W574, G121’, M124’, P197’, and F206’) in **Ia**–AHAS were more significant and relatively well-distributed. These residues were distributed around the biphenyl ring with similar non-polar contact distances. On the other hand, R377 moved farther away from **Ia**, so its Δ*E*_VdW_ contribution was relatively insignificant. In **IIa**–AHAS, the Δ*E*_VdW_ contributions were concentrated on residues W574 and K256’ (−15.755 kJ/mol and −7.974 kJ/mol, respectively). This was because the biphenyl moiety moved toward W574 and away from the other residues during the MD process. In **IIIa**–AHAS, the Δ*E*_VdW_ contributions of residues W574, K256’, and P197’ were significant since the pyrimidine ring of **IIIa** had a π-π interaction with W574 and the biphenyl group was close to K256’ and P197’.

Figure 6B shows the contributions of residues to electrostatic interaction energy (Δ*E*_ELE_). **IIIa** formed the strongest electrostatic interaction with residue R377 (−16.069 kJ/mol). Obviously, this was due to the formation of a bidentate H-bond between R377 and two nitrogen atoms of the pyrimidine ring in **IIIa**. In contrast, **IIa** displayed relatively weaker interaction (−2.093 kJ/mol) while electrostatic repulsion existed between **Ia** and R377 (+1.855 kJ/mol). In addition, K256’ exhibited significant Δ*E*_ELE_ contributions in all three systems by interacting with the pyrimidine ring (**Ia** and **IIa**) or oxygen atom (**IIIa**). Interestingly, the electrostatic contribution of W574 was −3.601 kJ/mol in **IIa**–AHAS but negligible in **Ia**–AHAS and **IIIa**–AHAS.

As shown in Figure 6C, with the exception of residue R377 (−2.287 kJ/mol) in **Ia**–AHAS, R377, K256’, and ligands all had positive Δ*G*_SOL_ values. In three systems, for residue K256’, the desolvation cost in **IIa**–AHAS was bigger than that in **Ia**–AHAS and **IIIa**–AHAS, but **Ia**–AHAS and **IIIa**–AHAS were comparable. In addition, the desolvation costs of both R377 and ligand decreased in this order: **Ia**–AHAS < **IIa**–AHAS < **IIIa**–AHAS. Notably, the desolvation costs of R377, K256’, and ligand in **Ia**–AHAS were much lower than those in **IIa**–AHAS and **IIIa**–AHAS, indicating a more favorable binding of **Ia** to AHAS.

**Figure 7:**
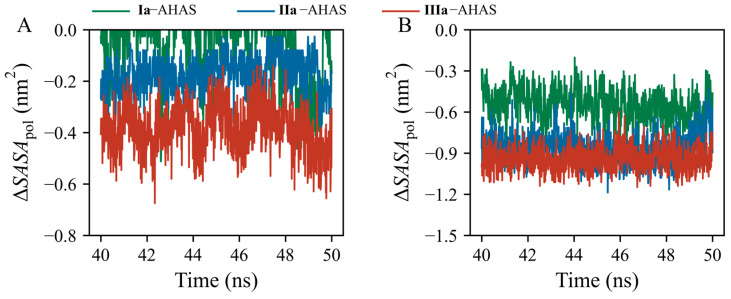
Time-evolution of polar SASA changes (∆*SASA*_pol_) of (**A**) R377 and (**B**) ligands.

The standard variations in Δ*G*_SOL_ contributions of R377, K256’, and ligands among the three systems were 12.2, 4.4, and 13.4, respectively. This indicated that R377 and ligands contributed to Δ*G*_SOL_ to a larger extent than K256’. Thus, the important roles of residue R377 and ligands in the desolvation cost should be explored and clarified by analyzing changes in solvent-accessible surface areas (SASAs). R377 is a positively charged polar amino acid residue with a high aqueous pKa value (~13.8) [29], imparting its hydrophilicity and making it difficult to dehydrate. Upon ligand binding to AHAS, the polar guanidino group of R377 dissociated from water and bound to the ligand, during which the existing interaction of R377 with water was weakened, leading to its destabilization. In addition, the pyrimidine moiety is also hydrophilic and needs to overcome the desolvation cost when bound to AHAS. Thus, we monitored polar SASA changes (Δ*SASA*_pol_) for R377 and ligands in the three systems over a time range of 40–50 ns (Figure 7). Δ*SASA*_pol_ was calculated as the difference in polar SASA between bound and unbound states (Equation (2)). In the three systems, the values of Δ*SASA*_pol_ for R377 and ligands were negative (Figure 7A,B), indicating that both R377 and ligands buried a part of their polar surface area into the complex interior upon binding to AHAS. The order of *SASA*_pol_ decrements (i.e., decrease in burial of polar surface area) for both R377 and ligands was **IIIa**–AHAS > **IIa**–AHAS > **Ia**–AHAS (Figure 7A,B). Therefore, in combination with the per-residue decomposition of desolvation cost (Figure 6C), it could be inferred that the lower desolvation cost in **Ia**–AHAS compared to those in **IIa**–AHAS and **IIIa**–AHAS was due to the burial of the smaller polar surface area of R377 and **Ia** in the complex interior upon binding [30].

Based on the above-mentioned findings, the inhibitory mechanism of PMBs was revealed. Upon their binding to AHAS, two possible binding modes, i.e., pose-1 and pose-2, could be adopted by PMBs depending on the position of the δ-ring. Pose-1 was generally more favorable than pose-2. More specifically, in pose-1, the biphenyl moiety penetrated deeper into the hydrophobic cavity of AHAS, and the pyrimidine ring remained exposed to the solvent, thus avoiding the destabilization caused by the desolvation process. At the same time, the hydrophilic residue R377 was far away from the ligand and was therefore more likely to be stabilized by water. Furthermore, in pose-1, van der Waals interaction was the driving force for binding and was stronger and more favorable than electrostatic interaction.

Taken together, compound **Ia** was ultimately selected as a lead compound and an ideal starting point for further optimization. It was particularly important to emphasize that lipophilic PMB **Ia** had the following characteristics: a favorable binding model (pose-1), lower binding free energy, and higher in vitro AHAS inhibitory activity, as well as good in vivo herbicidal activity (see Table S1 for the full data set).

With **Ia** in hand, we initiated our structural optimization by evaluating a variety of substituents at the δ-ring. This process was facilitated by the virtual screening and a simple, library-friendly synthesis. Firstly, the eighteen output structures **Iba-ic** containing various substituents at the δ-ring were obtained based on the calculated binding free energy (Δ*G*_bind_) (Table 2, see Table S3 for the full data set). Compared with **Ia**, some virtual hits **Iba**, **Ibc**, **Ica**, **Icb**, **Icc**, **Igc**, **Iha**, and **Iia** had much lower Δ*G*_bind_ values, ranging from -138.720 to -145.547 kJ/mol. Furthermore, eighteen compounds were synthesized using a one-step Suzuki–Miyaura coupling reaction.

As outlined in Table 2, **5** was prepared according to the literature [31] and then reacted with various substituted phenylboronic acids **6ba-ic** to afford the corresponding target compounds **Iba-ic**, giving moderate to excellent yields.

With the eighteen output structures **Iba-ic** in hand, we began exploring the structure–activity relationships. The pre- and post-emergence herbicidal activity of compounds were evaluated against four representative weeds at 750 g ai/ha under greenhouse conditions (Table 2, see Table S2 for the full data set). Generally, the total herbicidal activity of these compounds in pre-emergence was superior to that of lead compound **Ia**, but the potency in post-emergence was related to the nature and position of the substituents. The introduction of 2-substituents in **Ia** with a methyl group (**Iba**), methoxy group (**Ica**), and trifluoromethyl group (**Iia**) improved total post-emergence herbicidal activity, but other 2-substituents gave either comparable (**Iea**: 2-OH) or reduced (**Ida**: 2-NH_2_; **Ifa**: 2-F; **Iga**: 2-Cl; **Iha**: 2-Br) activity relative to **Ia**. In contrast, 3-substituted analogs (**Icb**: 3-OMe; **Idb**: 3-NH_2_) were beneficial, while another (**Ibb**: 3-Me) showed a minor decrease in activity. We found that functionalizing 4-positions with a methyl group (**Ibc**), methoxy group (**Icc**), and chlorine (**Igc**) performed effectively, and others (**Idc**: 4-NH_2_; **Ifc**: 4-F; **Iic**: 4-CF_3_) had a slight decrease in total herbicidal activity, but were also acceptable.

Interestingly, the herbicidal spectrum of **Iba-ic** was slightly different from that of **Ia** but provided promising levels of grass weed control. For example, six compounds **Iba** (2-Me), **Ica** (2-OMe), **Icb** (3-OMe), **Icc** (4-OMe), **Iha** (2-Br), and **Iia** (2-CF_3_) displayed even 98–100% inhibition against grass weeds (EC/DS), indicating that the introduction of a methoxy group and other hydrophobic groups (Me, CF_3_, Br) significantly increased the inhibition rate compared with **Ia**. All of these compounds were within the range of virtual hits predicted by Δ*G*_bind_ (Table 2), with up to a 75% hit rate for the virtual screen. Moreover, **Ica** (2-OMe), **Icb** (3-OMe), and **Icc** (4-OMe) all showed 100% inhibition against EC and DS, indicating that -OMe is the most promising group.

To assess whether the target compounds had the potential to be developed as graminicidal herbicides, we further tested the herbicidal activity of compounds **Iba**, **Ica**, **Icb**, **Icc**, **Iha**, and **Iia** against EC and DS at application rates of 187.5–375 g ai/ha. The positive control was selected as 2-((4,6-dimethoxypyrimidin-2-yl)oxy)benzoic acid (PYB1), an AHAS inhibitor [32]. As shown in Table 3, at the rate of 375 g ai/ha, all compounds showed moderate to excellent herbicidal activity. When the spraying dosage was reduced to 187.5 g ai/ha, **Ica** still exhibited 85% control against EC, and 80% control against DS, respectively, indicating that its herbicidal potency was comparable to PYB1. These promising findings indicated that compound **Ica** has great potential to be developed as a new class of carboxyl-free PMB herbicides.

Furthermore, the decomposition of the binding free energy (Table S3) showed that **Ica** had a much lower desolvation cost (∆*G*_SOL_ = 76.363 kJ/mol), while the van der Waals interaction (∆*E*_VdW_ = −208.265 kJ/mol) played a more important role than the electrostatic interaction (∆*E*_ELE_ = −13.654 kJ/mol), indicating that the non-polar interaction was more favorable for the binding **Ica** to AHAS. Another possible explanation for the high herbicidal activity of **Ica** is the enhanced preorganization of the biphenyl group [33]. The methoxy group in **Ica** served to turn the γ-ring and δ-ring out of coplanarity to preorganize the active conformation of the biphenyl group [34]. In other words, the preferred conformation of bound and unbound **Ica** was similar, which facilitated its binding with AHAS [35]. The preorganization effect of *ortho*-substituted biphenyls also provides a rational explanation for the other high herbicidal activities of PMBs **Iba**, **Iha**, and **Iia**.

**Figure 8:**
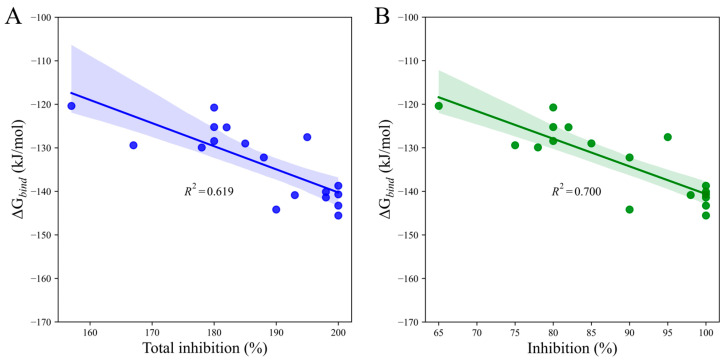
Linear regression plots depict the relationship between the calculated binding free energy of **Iba-ic** and the herbicidal activity against (**A**) EC and DS and (**B**) DS only. The shaded areas indicate the estimated confidence interval regions at a 95% confidence level around the true regression lines.

To assess the predictive power of our scoring function, we compared calculated binding free energy (∆*G*_bind_) to total post-emergence graminicidal activity for **Iba-ic**. We observed a reasonably good correlation (*R*^2^ = 0.619, *p*-value = 0.000) between the two data sets via simple regression analysis (Figure 8A). Interestingly, when considering DS only, the linear correlation observed was even better (*R*^2^ = 0.700, *p*-value = 0.000, Figure 8B). These findings highlighted the power of our scoring function in predicting the total post-emergence graminicidal activity for PMBs.

## 3. Discussion

AHAS-inhibiting herbicides greatly contribute to food production and sustainable agriculture and have attracted much attention from researchers. Most of these herbicides have carboxyl [14,15,16,17,36,37,38,39] or sulfonyl groups [19], which impart high polarity to them. In this case, polar interactions play an extremely important role in the binding of these herbicides to AHAS. However, hydrophobic interaction is another important type of interaction in molecular recognition that has not received enough attention in the design of herbicides [40]. In this work, the herbicidal design of lipophilic PMB **Ia** was accomplished by utilizing this important hydrophobic interaction. As a result, the lipophilic biphenyl moiety of **Ia** penetrated easily and deeply into the hydrophobic cavity of AHAS, forming extensive hydrophobic interactions with the non-polar residues via pose-1. Binding free energy calculation showed that van der Waals interaction plays a more important role in the binding of **Ia** with AHAS, further justifying the hydrophobic interaction. This pattern of hydrophobic interactions has been reported in inhibitors targeting other biosystems [41,42] but is unique to AHAS. Furthermore, the introduction of the methoxy group in the 2-position of the δ-ring regulated the torsion angle of the biphenyl close to the active conformation [33], making **Ica** bind more easily with AHAS and providing superior herbicidal activities compared with **Ia**.

On the other hand, in the process of using herbicides, formulation studies are required to improve weed control, in which adjuvants play a key role [43]. Specifically, the rational use of adjuvants has several advantages, such as improving the effectiveness of herbicides, reducing the amount of herbicides, enhancing the stability of herbicides, and minimizing the impact of herbicides on the environment [44,45,46,47]. In this work, the PMBs are formulated using DMSO as a solvent and Tween-80 as a nonionic surfactant that enables these compounds to penetrate the leaf surface wax and reach the plant tissue [48]. Obviously, intensive study of the formulations and the further optimization of adjuvants could enhance the effectiveness of pyrimidine-biphenyl herbicides.

Collectively, the novel chemical structure of the PMBs, along with their ease of synthesis, exceptional herbicidal activities, and new binding mode with AHAS pave a unique way for the development of AHAS-inhibiting herbicides.

## 4. Materials and Methods

### 4.1. Enzyme Assays

The expression and purification of wild-type *Arabidopsis thaliana* AHAS were performed according to the methods reported in the literature [49]. Specific activity determination assay in vitro was conducted as described by Fan et al. [50] with some modifications. **Ia**, **IIa**, **IIIa**, and PBA were dissolved in DMSO and diluted with ddH_2_O to the appropriate concentrations before use. To the enzyme solution (resulted by mixing 100 µL of 0.05 mM *At*AHAS, 50 µL of 0.5 mM K_2_HPO_4_-KH_2_PO_4_ buffer, 50 µL of 10 mM ThDP, 50 µL of 100 mM MgCl_2_, 5 µL of 1.0 mM FAD and 145 µL of ddH_2_O) was added 50 µL of compound solution followed by 50 µL 500 mM sodium pyruvate. The assays were performed in a final volume of 500 µL at 37 °C for 60 min. The enzymatic reaction was terminated by the addition of 37.5 µL of 2 M H_2_SO_4_ and incubation at 60 °C for 15 min. Then, 500 µL of freshly prepared 0.5% creatine and 5.0% α-naphthol in 2.5 M NaOH was added to the mixture, and the resulting solution was heated to 60 °C for another 15 min. The absorbance of the resulting red solution was tested at 525 nm. The inhibition rates and IC_50_ values of the tested compounds were calculated as described by Min et al. [51].

### 4.2. Herbicidal Activities under Greenhouse Conditions

The herbicidal activities were evaluated in the Green Pesticide Collaborative Innovation Center of Zhejiang A&F University (Hangzhou, China). All test compounds were dissolved in DMSO to 1.0% and then diluted with Tween-80 (0.1% aqueous solution) to the appropriate concentrations before use. The pre- and post-emergence herbicidal activities of the tested compounds were evaluated against two grass weeds, *Echinochloa crusgalli* (EC) and *Digitaria sanguinalis* (DS), and two broadleaf weeds, *Abutilon theophrasti* (AT) and *Cassia tora* (CT). Oxyfluorfen or 2-((4,6-dimethoxypyrimidin-2-yl)oxy)benzoic acid was selected as positive control. Final evaluation was performed within 21 days of treatment by visual rating scales of 0–100%. The remaining conditions were set in accordance with the methodology described in the literature [52].

### 4.3. Molecular Docking Study

The molecular docking simulation of compounds was performed using AutoDock 4.2 software [53]. The crystal structure of *Arabidopsis thaliana* AHAS monomer (PDB ID: 5K3S) [7] was obtained from the Protein Data Bank. The acceptor consisted of two chains, A and B. All water and ligands except for FAD and ThAthDP were removed from the structure, and a grid box with a volume of 3375 Å^3^ at the binding site was created. All residues of the receptor were set rigid, while the ligands were flexible. In total, 1000 runs clustered by 2 Å of root-mean-square deviation (RMSD) criteria were launched for each compound. The other parameters of the docking model were set as default. The binding scores generated from the built-in scoring function and the number of modes in the clusters were used as criteria to determine the final binding modes.

### 4.4. MD Simulation Study

Conventional molecular dynamics simulations of inhibitor–*At*AHAS complexes were performed using GROMACS 2018.4 software package [54] applying the CHARMM36 force field [55]. The topology files for ligands and cofactors were prepared from the CGenFF program [56,57]. A 2720 nm^3^ cubic box was created for each simulation system, charged with TIP3P waters [58]. Each system was neutralized by adding 32 Cl^−^ ions. All the systems were optimized using 2500 cycles of steepest descent energy minimization. Then, all systems were heated from 0 to 300 K with 1000 kJ/mol/nm^2^ force constant restraints on heavy atoms of protein, ligand, and cofactors, followed by 100 ps equilibrations in the NPT ensemble (*T* = 300 K and *P* = 1 bar). Finally, MD production runs without any constrain were performed in the NPT ensemble (*T* = 300 K and *P* = 1 bar) for each system.

### 4.5. Binding Free Energy Calculation

The binding free energy and per-residue free energy decomposition for the inhibitor–*At*AHAS systems were calculated using the molecular mechanics Poisson–Boltzmann surface area (MMPBAS) [28] method employing Adaptive Poisson–Boltzmann Solver (apbs) [59] module. The binding free energy (Δ*G*_bind_) referred to the difference between the free energy of the protein–ligand system and protein–ligand in the unbound state, which was calculated using the following formula:

Δ*G*_bind_ = Δ*E*_MM_ + Δ*G*_SOL_ − *T*Δ*S* (1)

where Δ*E*_MM_ is the change in potential energy during the formation of the complex in a vacuum and consists of van der Waals (Δ*E*_VdW_) and electrostatic (Δ*E*_ELE_) components. Δ*G*_SOL_ represents the change in solvation-free energy, which consists of polar (Δ*G*_P_) and non-polar (Δ*G*_NP_) parts. The entropic contribution − *T*Δ*S* for the systems was neglected in this study.

### 4.6. Solvent Accessible Surface Area (SASA) Calculation

SASA was determined using GROMACS SASA utility [60] with a 1.4 Å probe radius. Polar SASA (*SASA*_pol_) was defined as the total SASA of polar atoms (i.e., oxygens, nitrogens) and their attached hydrogen atoms [61]. Polar SASA change (∆*SASA*_pol_) was defined as:

∆*SASA*_pol_ = *SASA*_pol, bound_ − *SASA*_pol, unbound_ (2)

where *SASA*_pol, bound_ and *SASA*_pol, unbound_ are the polar SASA in bound and unbound states, respectively.

## 5. Conclusions

In conclusion, we have discovered a new class of lipophilic pyrimidine-biphenyl (PMB) herbicides by means of de novo design using the biphenyl as the molecular core and a subsequent integrated in vitro AHAS test, in vitro herbicidal activity evaluation, molecular docking, and MD simulation approach. To improve the efficiency of this computer-aided herbicide design, we carried out virtual screening by introducing our binding model for PMBs. As a consequence, the discovery of new PMB herbicides was successful to the extent that 15 out of 18 designed PMBs exhibited a 90–100% post-emergence control against grass weeds at 750 g ai/ha. In particular, **Ica** with lower binding free energy (Δ*G*_bind_ = −145.547 kJ/mol) was found to be effective in controlling grass weeds at lower dosages and had the potential to be developed into a graminicidal herbicide. We found that the herbicidal potency could be enhanced by lowering the desolvation cost and reinforcing more extensive hydrophobic contacts in the AHAS pocket for the design of new AHAS-inhibiting herbicides.

## Figures and Tables

**Table 1:** Calculated binding free energy and energy components of **Ia**, **Iia**, and **IIIa**.

System	Calculated Energy (kJ/mol)				
	Δ*E*_VdW_	Δ*E*_ELE_	Δ*E*_MM_	Δ*G*_SOL_	Δ*G*_bind_
**Ia**–AHAS	−190.013	−24.397	−214.410	89.455	−124.955
**IIa**–AHAS	−190.307	−42.914	−233.222	122.967	−110.255
**IIIa**–AHAS	−191.220	−68.200	−259.420	170.556	−88.864

**Table 2:**
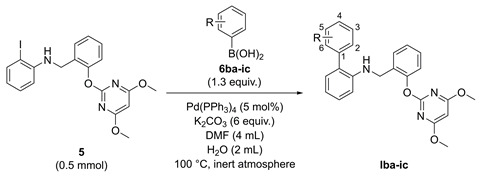
Synthesis, calculated binding free energy (Δ*G*_bind_), and herbicidal activities of **Iba-ic**.

Compd.	R	Δ*G*_bind_ (kJ/mol)	Inhibition ^a^ (Post-Emergence, %)				
			EC ^b^	DS	AT	CT	Total
**Ia**	-H	−124.955	60	85	85	85	315
**Iba**	2-Me	**−141.408 ^c^**	98	100	45	98	341
**Ibb**	3-Me	−128.998	100	85	45	80	310
**Ibc**	4-Me	**−144.177**	100	90	60	80	330
**Ica**	2-OMe	**−145.547**	100	100	50	88	338
**Icb**	3-OMe	**−138.720**	100	100	40	85	325
**Icc**	4-OMe	**−143.275**	100	100	70	88	358
**Ida**	2-NH_2_	−120.744	100	80	65	20	265
**Idb**	3-NH_2_	−125.291	100	82	65	90	337
**Idc**	4-NH_2_	−127.550	100	95	25	88	308
**Iea**	2-OH	−125.229	100	80	50	85	315
**Iec**	4-OH	−120.380	92	65	35	75	267
**Ifa**	2-F	−135.177	92	75	40	82	289
**Ifc**	4-F	−132.218	98	90	45	70	303
**Iga**	2-Cl	−128.437	100	80	40	80	300
**Igc**	4-Cl	**−140.856**	95	98	55	70	318
**Iha**	2-Br	**−140.702**	100	100	50	45	295
**Iia**	2-CF_3_	**−140.103**	98	100	60	80	338
**Iic**	4-CF_3_	−129.919	100	78	45	90	313

^a^ Herbicidal activities were tested at rates of 750 g ai/ha. ^b^ Abbreviations: EC, *Echinochloa crusgalli*; DS, *Digitaria sanguinalis*; AT, *Abutilon theophrasti*; CT, *Cassia tora.* ^c^ Virtual hits are shown in bold font.

**Table 3:** Dose-reduction trials of **Ica-cc**, **Iba**, **Iha**, and **Iia**.

Compd.	Dosage (g ai/ha)	Inhibition ^a^ (%)	
		EC ^b^	DS
**Iba**	375	90	80
	187.5	85	70
**Ica**	375	90	85
	187.5	85	80
**Icb**	375	80	70
	187.5	75	60
**Icc**	375	80	75
	187.5	75	70
**Iha**	375	85	70
	187.5	75	60
**Iia**	375	80	85
	187.5	70	65
PYB1 ^c^	375	90	90
	187.5	85	85

^a^ Inhibition rates in post-emergence treatment. ^b^ Abbreviations: EC, *Echinochloa crusgalli*; DS, *Digitaria sanguinalis*. ^c^ PYB1 (2-((4,6-dimethoxypyrimidin-2-yl)oxy)benzoic acid) was used as positive control.

## Data Availability

Data is contained within the article or Supplementary Materials.

## Supplementary Materials

The following supporting information can be downloaded at: https://www.mdpi.com/article/10.3390/molecules29112409/s1. Figure S1: Backbone root mean square deviation (RMSD) of AHAS in the simulation systems; Table S1: Herbicidal activities of **Ia**, **IIa**, and **IIIa**; Table S2: Herbicidal activities of **Ia** and **Iba-ic**; Table S3: Calculated binding free energy and energy components of **Ia** and **Iba-ic**; ^1^H NMR, ^13^C NMR, ^19^F NMR, and HRMS characterization of target compounds **Ia**, **IIa**, **IIIa**, and **Iba-ic**; and the original spectra for all the target compounds; Scheme S1: General procedure A for the synthesis of **Ia**, **IIa** and **IIIa**; Scheme S2: General procedure B for the synthesis of **Iba-ic**. Ref. [31] is cited in Supplementary Materials.
