# Supplementary material for: Discovery of a New Class of Lipophilic Pyrimidine-Biphenyl Herbicides Using an Integrated Experimental-Computational Approach
Source: Molecules. 2024 May 21;29(11):2409. doi: 10.3390/molecules29112409 (PMC11173721; doi:10.3390/molecules29112409)

Supporting Information

Discovery of a New Class of Lipophilic Pyrimidine-Biphenyl Herbicides Using an Integrated Experimental-Computational Approach

Yitao Yan [1,*,†], Yinglu Chen [1,†], Hanxian Hu [2], Youwei Jiang [3], Zhengzhong Kang [4] and Jun Wu [1,*]

[1] Department of Chemistry, Zhejiang University, Hangzhou 310058, China; ylchen94@zju.edu.cn
[2] School of Physics, Zhejiang University, Hangzhou 310027, China; 11936032@zju.edu.cn
[3] Hangzhou Jingyinkang Biological Technology Co., Ltd., Hangzhou 311110, China; jiangyw@genekine.com
[4] Beijing Life Science Academy, Beijing 102200, China; kangzhzh2007@126.com
* Correspondence: 11937068@zju.edu.cn (Y.Y.); wujunwu@zju.edu.cn (J.W.)
† These authors contributed equally to this work.

Table of contents

1. General Information S2
2. Experiment Section S3
3. Supplementary Tables S13
4. Supplementary Figure S15
5. Original NMR Spectra for **Ia**, **IIa**, **IIIa** and **Iba-ic** S16

1. General Information

Unless otherwise indicated, all reagents were purchased from commercial distributors and used without further purification. Flash column chromatography was performed using silica gel (300-400 mesh). Thin layer chromatography (TLC) was performed using glass 0.25 mm silica gel GF254 plates. Nuclear magnetic resonance (NMR) spectra were recorded with a Bruker AVANCE 400 MHz instrument. ^{1}H, and ^{13}C chemical shifts are reported in ppm downfield of tetramethylsilane and referenced to residual solvent peak ($CHCl_3$ = 7.26 ppm for ^{1}H NMR, 77.16 ppm for ^{13}C NMR) unless otherwise noted. ^{19}F NMR chemical shifts were determined relative to internal standard ($CFCl_3$= 0.0). Multiplicities are reported using the following abbreviations: s = singlet, d = doublet, t = triplet, q = quartet, m = multiplet, dd = doublet of doublets, td = triplet of doublets. High resolution mass spectra (HRMS) were recorded on WATERS GCT Premier using EI-TOF or SHIMADZU LCMS-IT-TOF using ESI-TOF operating in positive ion mode. Melting points were determined using WRR apparatus and not corrected.

2. Experiment Section

2.1 General Procedure A for the Synthesis of Ia, IIa and IIIa

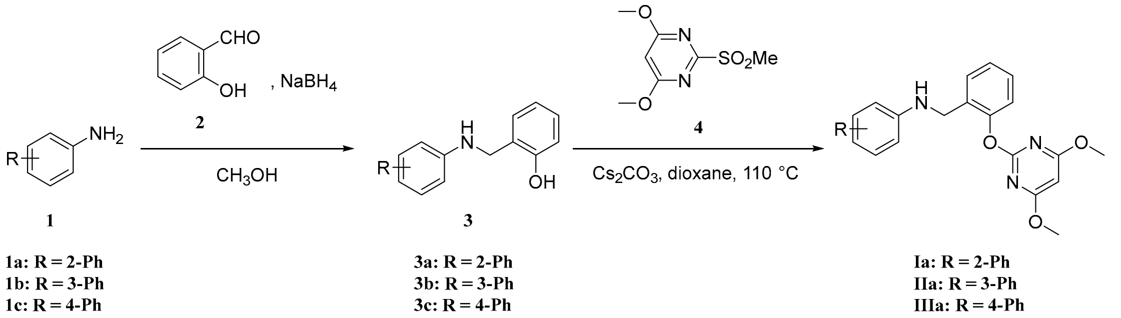

Scheme S1. General procedure A for the synthesis of **Ia**, **IIa** and **IIIa**.

General procedure A: After aminobiphenyl **1** (1.0 mmol) was dissolved in methanol (6 mL), salicylaldehyde (**2**, 1.2 mmol) was added dropwise. The mixture was stirred for 30 min until complete consumption of **1**, as detected by TLC. Subsequently, $NaBH_4$ (1.5 mmol) was slowly added to the resulted mixture. After the reaction was completed, the solution was concentrated in vacuum. The crude mixture was dissolved in 1,4-dioxane (6 mL) followed by the addition of Cs_2CO_3 (1.5 mmol) and **4** (1.2 mmol). The solution was heated at 110 °C for 4 h until complete consumption of **3**. Afterwards, the reaction mixture was quenched with brine. The aqueous layer was extracted with ethyl acetate (3×10 mL), and combined organic extracts were dried over Na_2SO_4. After filtration and evaporation of the solvents under vacuum, the crude product was purified by column chromatography on silica gel with petroleum ether/ethyl acetate (V/V=10/1) as the eluent.

Analytical data for the products **Ia**, **IIa** and **IIIa**:

***N*-(2-((4,6-dimethoxypyrimidin-2-yl)oxy)benzyl)-[1,1'-biphenyl]-2-amine (Ia)**

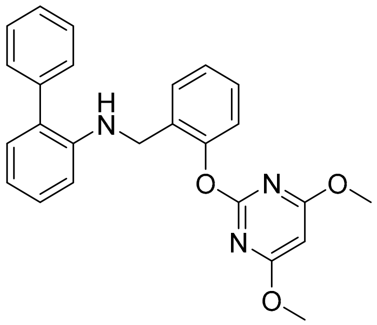

The title compound **Ia** was prepared from [1,1'-biphenyl]-2-amine (**1a**) according to general procedure A. **Ia** was obtained as colorless oil (340 mg, yield 82%). ^{1}H NMR (400 MHz, $CDCl_3$) δ 7.45 – 7.38 (m, 5H), 7.37 – 7.24 (m, 2H), 7.21 – 7.06 (m, 4H), 6.78 (t, J = 7.1 Hz, 1H), 6.66 (d, J = 8.1 Hz, 1H), 5.76 (s, 1H), 4.33 (s, 2H), 3.77 (s, 6H). ^{13}C NMR (101 MHz, $CDCl_3$) δ 172.91, 164.04, 150.81, 144.03, 139.16, 131.06, 130.19, 129.31, 128.91, 128.58, 128.45, 128.08, 128.01, 127.28, 125.63, 122.54, 117.72, 111.48, 84.72, 54.16, 43.57. HRMS m/z (ESI-TOF): calcd for $C_{25}H_{24}N_3O_3$ $(M+H)^+$: 414.1812; found 414.1814.

***N*-(2-((4,6-dimethoxypyrimidin-2-yl)oxy)benzyl)-[1,1'-biphenyl]-3-amine (IIa)**

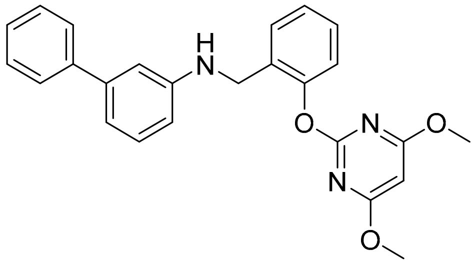

The title compound **IIa** was prepared from [1,1'-biphenyl]-3-amine (**1b**) according to general procedure A. **IIa** was obtained as white solid (367 mg, yield 88%; mp: 126.5-127.2°C). ^{1}H NMR (400 MHz, $CDCl_3$) δ 7.54 – 7.47 (m, 3H), 7.43 – 7.36 (m, 2H), 7.35 – 7.29 (m, 2H), 7.26 – 7.13 (m, 3H), 6.92 (ddd, *J* = 7.6, 17, 1.0 Hz, 1H), 6.77 (t, *J* = 2.1 Hz, 1H), 6.55 (dd, *J* = 7.7, 1.9 Hz, 1H), 5.76 (s, 1H), 4.41 (s, 2H), 3.77 (s, 6H). ^{13}C NMR (101 MHz, $CDCl_3$) δ 173.10, 164.41, 151.12, 148.43, 142.38, 141.80, 131.65, 129.60, 129.17, 128.69, 128.33, 127.24, 125.84, 122.84, 116.74, 111.97, 111.83, 84.71, 54.31, 43.61. HRMS m/z (ESI-TOF): calcd for $C_{25}H_{24}N_3O_3$ $(M+H)^+$: 414.1812; found 414.1815.

***N*-(2-((4,6-dimethoxypyrimidin-2-yl)oxy)benzyl)-[1,1'-biphenyl]-4-amine (IIIa)**

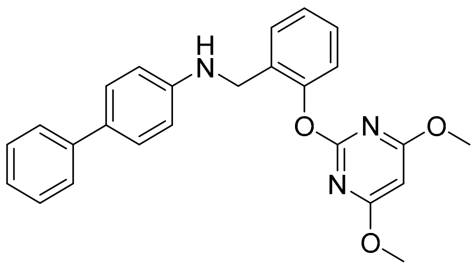

The title compound **IIIa** was prepared from [1,1'-biphenyl]-4-amine (**1c**) according to general procedure A. **IIIa** was obtained as white solid (326 mg, yield 78%; mp: 123.0-123.6°C). ^{1}H NMR (400 MHz, $CDCl_3$) δ 7.53 – 7.46 (m, 3H), 7.41 – 7.35 (m, 4H), 7.32 (td, *J* = 7.7, 1.8 Hz, 1H), 7.30 – 7.17 (m, 2H), 7.16 (dd, *J* = 8.0, 1.3 Hz, 1H), 6.65 – 6.59 (m, 2H), 5.79 (s, 1H), 4.38 (s, 2H), 3.81 (s, 6H). ^{13}C NMR (101 MHz, $CDCl_3$) δ 173.13, 164.41, 151.11, 147.45, 141.34, 131.60, 130.49, 129.09, 128.74, 128.35, 127.92, 126.38, 126.16, 125.87, 122.85, 113.25, 84.74, 54.33, 43.54. HRMS m/z (EI-TOF): calcd for $C_{25}H_{23}N_3O_3$ $(M)^+$: 413.1739; found 413.1737.

2.2 General Procedure B for the Synthesis of Iba-ic.

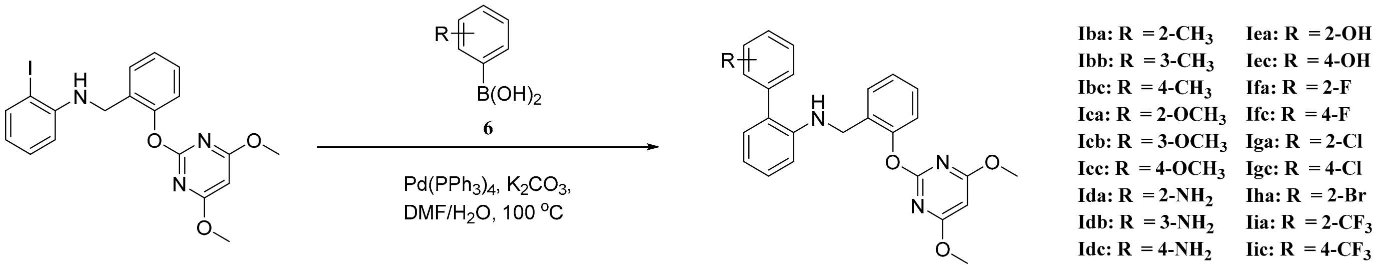

5 **I**

Scheme S2. General procedure B for the synthesis of **Iba-ic**.

General procedure B: Compound **5a** was synthesized base on a known procedure [1]. To a solution of compound **5** (0.5 mmol) and phenylboronic acid **6** (0.65 mmol) in DMF (4 ml) at room temperature was added $Pd(PPh_3)_4$ (0.025 mmol) under argon, followed by the addition of K_2CO_3 (3 mmol) in H_2O (2 ml). The resulting mixture was heated at 100 °C for 1 hour. Afterwards, the reaction mixture was quenched with brine. The aqueous layer was extracted with ethyl acetate (3×10 mL), and combined

organic extracts were dried over Na_2SO_4. After filtration and evaporation of the solvents under vacuum, the crude product was purified by column chromatography on silica gel with petroleum ether/ethyl acetate (V/V=10/1) as the eluent.

Analytical data for the products **Iba-ic**:

***N*-(2-((4,6-dimethoxypyrimidin-2-yl)oxy)benzyl)-2'-methyl-[1,1'-biphenyl]-2-amine (Iba)**

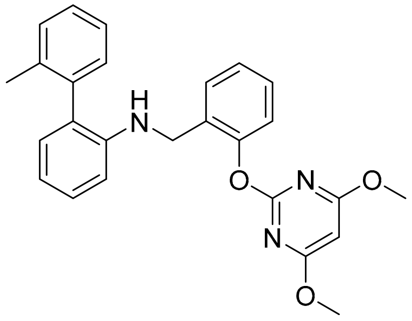

The title compound **Iba** was prepared from *o*-tolylboronic acid (**6ba**) according to general procedure B. **Iba** was obtained as colorless oil (197 mg, yield 91%). ^1H NMR (400 MHz, $CDCl_3$) δ 7.35 (d, *J* = 7.6 Hz, 1H), 7.30 – 7.21 (m, 4H), 7.20 – 7.08 (m, 4H), 6.98 (dd, *J* = 7.4, 1.5 Hz, 1H), 6.75 (t, *J* = 7.3 Hz, 1H), 6.62 (d, *J* = 8.1 Hz, 1H), 5.77 (s, 1H), 4.31 (s, 2H), 3.78 (s, 6H), 2.15 (s, 3H). ^{13}C NMR (101 MHz, $CDCl_3$) δ 172.97, 164.17, 150.73, 144.94, 138.48, 137.26, 131.79, 130.35, 130.30, 129.74, 128.40, 128.06, 127.80, 127.30, 126.29, 125.63, 122.51, 116.87, 110.52, 84.75, 54.16, 42.97, 19.79. HRMS m/z (EI-TOF): calcd for $C_{26}H_{25}N_3O_3$ (M^+): 427.1896; found 427.1895.

***N*-(2-((4,6-dimethoxypyrimidin-2-yl)oxy)benzyl)-3'-methyl-[1,1'-biphenyl]-2-amine (Ibb)**

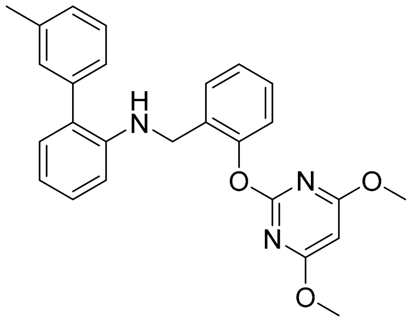

The title compound **Ibb** was prepared from *m*-tolylboronic acid (**6bb**) according to general procedure B. **Ibb** was obtained as light yellow oil (215 mg, yield 100%). ^1H NMR (400 MHz, $CDCl_3$) δ 7.44 (d, *J* = 7.5 Hz, 1H), 7.37 – 7.21 (m, 4H), 7.23 – 7.07 (m, 5H), 6.75 (td, *J* = 7.4, 1.1 Hz, 1H), 6.61 (d, *J* = 8.1 Hz, 1H), 5.78 (s, 1H), 4.35 (s, 2H), 3.78 (s, 6H), 2.41 (s, 3H). ^{13}C NMR (101 MHz, $CDCl_3$) δ 173.00, 164.20, 150.84, 144.76, 139.40, 138.67, 131.73, 130.17, 128.80, 128.52, 128.29, 128.05, 127.91, 127.85, 126.34, 125.70, 122.60, 117.16, 110.91, 84.76, 54.20, 43.26, 21.58. HRMS m/z (EI-TOF): calcd for $C_{26}H_{25}N_3O_3$ (M^+): 427.1896; found 427.1897.

***N*-(2-((4,6-dimethoxypyrimidin-2-yl)oxy)benzyl)-4'-methyl-[1,1'-biphenyl]-2-amine (Ibc)**

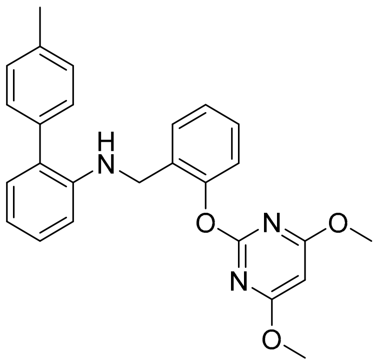

The title compound **Ibc** was prepared from *p*-tolylboronic acid (**6bc**) according to general procedure B. **Ibc** was obtained as white solid (199 mg, yield 92%; mp: 82.6-83.9°C). ^{1}H NMR (400 MHz, $CDCl_3$) δ 7.44 – 7.37 (m, 1H), 7.32 – 7.24 (m, 3H), 7.22 (d, *J* = 7.9 Hz, 2H), 7.20 – 7.15 (m, 1H), 7.14 – 7.08 (m, 2H), 7.06 (dd, *J* = 7.4, 1.5 Hz, 1H), 6.74 (td, *J* = 7.4, 1.0 Hz, 1H), 6.62 (d, *J* = 8.1 Hz, 1H), 5.76 (s, 1H), 4.32 (s, 2H), 3.76 (s, 6H), 2.39 (s, 3H). ^{13}C NMR (101 MHz, $CDCl_3$) δ 172.97, 164.19, 150.86, 144.86, 136.90, 136.44, 131.67, 130.22, 129.66, 129.24, 128.43, 127.92, 127.67, 125.68, 122.62, 117.15, 110.81, 84.78, 54.18, 43.31, 21.27. HRMS m/z (EI-TOF): calcd for $C_{26}H_{25}N_3O_3$ (M^+): 427.1896; found 427.1898.

***N*-(2-((4,6-dimethoxypyrimidin-2-yl)oxy)benzyl)-2'-methoxy-[1,1'-biphenyl]-2-amine (Ica)**

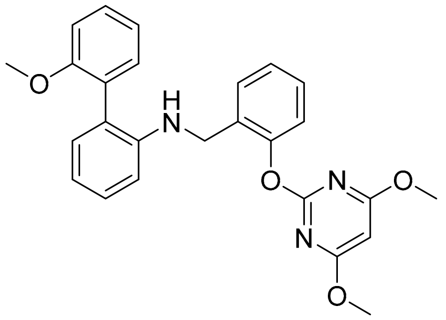

The title compound **Ica** was prepared from (2-methoxyphenyl)boronic acid (**6ca**) according to general procedure B. **Ica** was obtained as light yellow oil (220 mg, yield 98%). ^{1}H NMR (400 MHz, $CDCl_3$) δ 7.45 (d, *J* = 7.5 Hz, 1H), 7.36 (td, *J* = 8.2, 1.7 Hz, 1H), 7.30 – 7.22 (m, 2H), 7.20 – 6.96 (m, 6H), 6.76 (t, *J* = 7.2 Hz, 1H), 6.61 (d, *J* = 7.8 Hz, 1H), 5.78 (s, 1H), 4.35 (d, *J* = 4.1 Hz, 2H), 3.82 (s, 3H), 3.80 (s, 6H). ^{13}C NMR (101 MHz, $CDCl_3$) δ 173.02, 164.20, 156.92, 150.72, 145.34, 132.08, 131.99, 130.75, 129.13, 128.57, 128.02, 127.72, 125.68, 124.67, 122.40, 121.25, 116.93, 111.19, 110.72, 84.68, 55.67, 54.24, 42.77. HRMS m/z (EI-TOF): calcd for $C_{26}H_{25}N_3O_4$ (M^+): 443.1845; found 443.1843.

***N*-(2-((4,6-dimethoxypyrimidin-2-yl)oxy)benzyl)-3'-methoxy-[1,1'-biphenyl]-2-amine (Icb)**

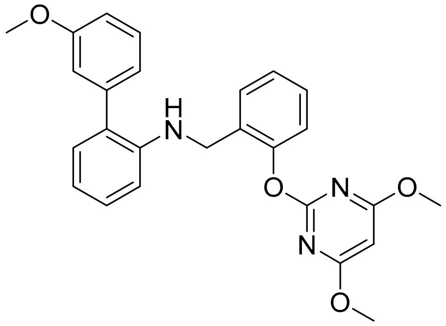

The title compound **Icb** was prepared from (3-methoxyphenyl)boronic acid (**6cb**) according to general procedure B. **Icb** was obtained as white solid (215 mg, yield 96%; mp: 118.5-120.1°C). ^{1}H NMR (400 MHz, $CDCl_3$) δ 7.43 (d, *J* = 7.5 Hz, 1H), 7.34 (t, *J* = 7.9 Hz, 1H), 7.28 (t, *J* = 6.6 Hz, 1H), 7.19 (t, *J* = 7.4 Hz, 1H), 7.16 – 7.08 (m, 3H), 7.02 (d, *J* = 7.6 Hz, 1H), 6.98 (s, 1H), 6.89 (dd, *J* = 8.6, 2.2 Hz, 1H), 6.75 (t, *J* = 7.4 Hz, 1H), 6.62 (d, *J* = 8.1 Hz, 1H), 5.77 (s, 1H), 4.34 (s, 2H), 3.84 (s, 3H), 3.78 (s, 6H).

^{13}C NMR (101 MHz, $CDCl_3$) δ 173.00, 164.19, 160.06, 150.87, 144.68, 140.86, 131.64, 130.09, 129.96, 128.70, 128.34, 127.97, 127.58, 125.71, 122.63, 121.64, 117.17, 114.83, 113.04, 110.96, 84.78, 55.33, 54.22, 43.25. HRMS m/z (EI-TOF): calcd for $C_{26}H_{25}N_3O_4$ (M^+): 443.1845; found 443.1843.

***N*-(2-((4,6-dimethoxypyrimidin-2-yl)oxy)benzyl)-4'-methoxy-[1,1'-biphenyl]-2-amine (Icc)**

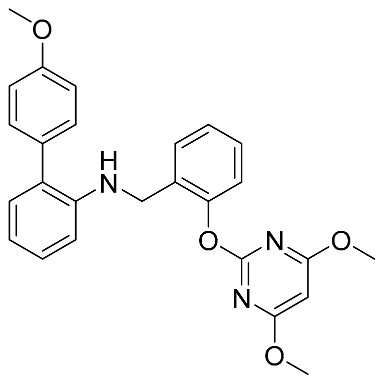

The title compound **Icc** was prepared from (4-methoxyphenyl)boronic acid (**6cc**) according to general procedure B. **Icc** was obtained as white solid (215 mg, yield 96%; mp: 104.8-105.3°C). ^{1}H NMR (400 MHz, $CDCl_3$) δ 7.40 (dd, *J* = 7.6, 1.1 Hz, 1H), 7.31 (d, *J* = 8.6 Hz, 2H), 7.27 (td, *J* = 7.9, 1.6 Hz, 1H), 7.17 (td, *J* = 7.5, 1.0 Hz, 1H), 7.14 – 7.08 (m, 2H), 7.05 (dd, *J* = 7.4, 1.5 Hz, 1H), 6.95 (d, *J* = 8.7 Hz, 2H), 6.75 (t, *J* = 7.4 Hz, 1H), 6.64 (d, *J* = 8.1 Hz, 1H), 5.76 (s, 1H), 4.32 (s, 2H), 3.84 (s, 3H), 3.76 (s, 6H). ^{13}C NMR (101 MHz, $CDCl_3$) δ 173.01, 164.21, 158.87, 150.92, 144.84, 131.58, 131.55, 130.52, 130.32, 128.53, 128.39, 128.02, 127.59, 125.72, 122.66, 117.36, 114.40, 111.02, 84.81, 55.38, 54.25, 43.49. HRMS m/z (EI-TOF): calcd for $C_{26}H_{25}N_3O_4$ (M^+): 443.1845; found 443.1847.

***N*²-(2-((4,6-dimethoxypyrimidin-2-yl)oxy)benzyl)-[1,1'-biphenyl]-2,2'-diamine (Ida)**

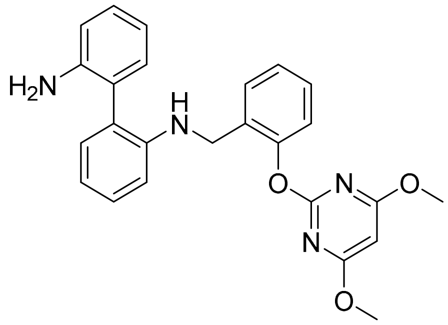

The title compound **Ida** was prepared from (2-aminophenyl)boronic acid (**6da**) according to general procedure B. **Ida** was obtained as light yellow solid (210 mg, yield 97%; mp: 84.8-87.0°C). ^{1}H NMR (400 MHz, $CDCl_3$) δ 7.39 (d, *J* = 7.6 Hz, 1H), 7.26 (dd, *J* = 7.7, 1.6 Hz, 1H), 7.21 – 7.06 (m, 6H), 6.82 (td, *J* = 7.4, 1.0 Hz, 1H), 6.80 – 6.72 (m, 2H), 6.62 (d, *J* = 8.1 Hz, 1H), 5.78 (s, 1H), 4.33 (d, *J* = 1.8 Hz, 2H), 3.78 (s, 6H). ^{13}C NMR (101 MHz, $CDCl_3$) δ 173.03, 164.19, 150.81, 145.21, 144.36, 131.68, 131.28, 130.79, 128.98, 128.97, 128.22, 127.93, 125.71, 124.41, 124.30, 122.55, 118.98, 117.50, 115.73, 111.08, 84.75, 54.30, 43.03. HRMS m/z (EI-TOF): calcd for $C_{25}H_{24}N_4O_3$ (M^+): 428.1848; found 428.1849.

***N*²-(2-((4,6-dimethoxypyrimidin-2-yl)oxy)benzyl)-[1,1'-biphenyl]-2,3'-diamine (Idb)**

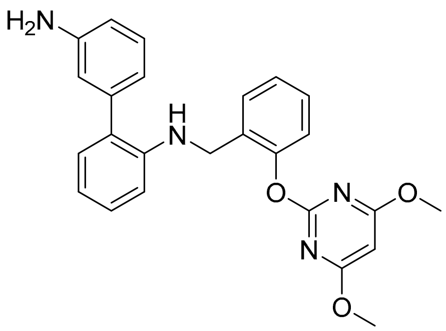

The title compound **Idb** was prepared from (3-aminophenyl)boronic acid (**6db**) according to general procedure B. **Idb** was obtained as light yellow solid (202 mg, yield 93%; mp: 51.7-53.8°C). ^{1}H NMR (400 MHz, $CDCl_3$) δ 7.44 (d, *J* = 7.5 Hz, 1H), 7.29 (td, *J* = 7.7, 1.4 Hz, 1H), 7.24 – 7.17 (m, 2H), 7.16 – 7.08 (m, 3H), 6.82 (d, *J* = 7.6 Hz, 1H), 6.79 (s, 1H), 6.74 (t, *J* = 7.4 Hz, 1H), 6.66 (d, *J* = 7.8 Hz, 1H), 6.61 (d, *J* = 8.0 Hz, 1H), 5.80 (s, 1H), 4.35 (s, 2H), 3.80 (s, 6H). ^{13}C NMR (101 MHz, $CDCl_3$) δ 172.96, 164.14, 150.82, 146.94, 144.77, 140.46, 131.70, 129.88, 129.83, 128.39, 127.87, 127.84, 125.64, 122.54, 119.37, 116.94, 115.93, 113.99, 110.74, 84.63, 54.19, 43.31. HRMS m/z (ESI-TOF): calcd for $C_{25}H_{25}N_4O_3$ $(M+H)^+$: 429.1921; found 429.1924.

N^2-(2-((4,6-dimethoxypyrimidin-2-yl)oxy)benzyl)-[1,1'-biphenyl]-2,4'-diamine (Idc)

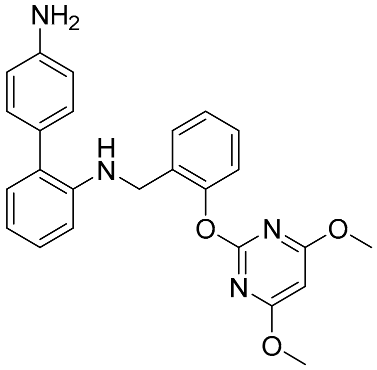

The title compound **Idc** was prepared from (4-aminophenyl)boronic acid (**6dc**) according to general procedure B. **Idc** was obtained as brown oil (210 mg, yield 97%). ^{1}H NMR (400 MHz, $CDCl_3$) δ 7.41 (dd, *J* = 7.5, 1.3 Hz, 1H), 7.27 (td, *J* = 7.8, 1.7 Hz, 1H), 7.23 (d, *J* = 8.4 Hz, 2H), 7.18 (td, *J* = 7.5, 1.1 Hz, 1H), 7.14 – 7.02 (m, 3H), 6.80 (d, *J* = 8.3 Hz, 2H), 6.72 (td, *J* = 7.4, 1.0 Hz, 1H), 6.57 (d, *J* = 8.1 Hz, 1H), 5.77 (s, 1H), 4.31 (s, 2H), 3.78 (s, 6H). ^{13}C NMR (101 MHz, $CDCl_3$) δ 173.02, 164.23, 150.82, 145.10, 144.39, 131.81, 130.42, 130.23, 128.35, 128.17, 127.90, 127.71, 125.74, 122.60, 117.13, 116.15, 110.73, 84.76, 54.25, 43.27. HRMS m/z (EI-TOF): calcd for $C_{25}H_{24}N_4O_3$ (M^+): 428.1848; found 428.1846.

2'-((2-((4,6-dimethoxypyrimidin-2-yl)oxy)benzyl)amino)-[1,1'-biphenyl]-2-ol (Iea)

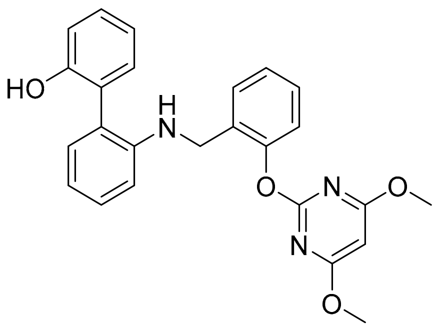

The title compound **Iea** was prepared from (2-hydroxyphenyl)boronic acid (**6ea**) according to general procedure B. **Iea** was obtained as light yellow oil (164 mg, yield 75%). ^{1}H NMR (400 MHz, $CDCl_3$) δ 7.34 – 7.13 (m, 7H), 7.12 (d, *J* = 8.0 Hz, 1H), 7.05 – 6.95 (m, 2H), 6.90 (td, *J* = 7.5, 0.9 Hz, 1H), 6.77 (d, *J* = 9.1 Hz, 1H), 5.77 (s, 1H), 4.26 (s, 2H), 3.75 (s, 6H). ^{13}C NMR (101 MHz, $CDCl_3$) δ 173.04, 164.14, 153.93, 151.06, 144.40, 131.78, 131.20, 130.63, 129.57, 129.15, 128.51, 125.94, 125.87,

125.72, 122.73, 121.12, 119.93, 117.47, 113.96, 84.85, 54.33, 44.21. HRMS m/z (ESI-TOF): calcd for $C_{25}H_{24}N_3O_4$ $(M+H)^+$: 430.1761; found 430.1760.

2'-((2-((4,6-dimethoxypyrimidin-2-yl)oxy)benzyl)amino)-[1,1'-biphenyl]-4-ol (Iec)

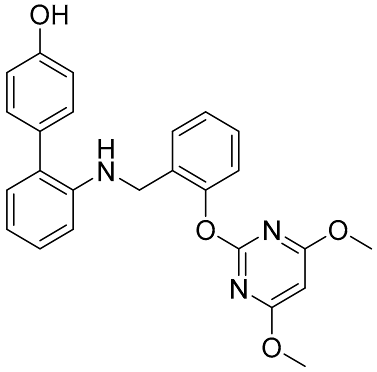

The title compound **Iec** was prepared from (4-hydroxyphenyl)boronic acid (**6ec**) according to general procedure B. **Iec** was obtained as yellow solid (195 mg, yield 90%; mp: 52.4-54.2°C). ^{1}H NMR (400 MHz, $CDCl_3$) δ 7.43 (dd, *J* = 7.6, 1.4 Hz, 1H), 7.35 – 7.23 (m, 3H), 7.20 (td, *J* = 7.5, 1.2 Hz, 1H), 7.18 – 7.11 (m, 2H), 7.07 (dd, *J* = 7.4, 1.6 Hz, 1H), 6.94 – 6.89 (m, 2H), 6.76 (td, *J* = 7.4, 1.0 Hz, 1H), 6.64 (d, *J* = 8.1 Hz, 1H), 5.82 (s, 1H), 4.35 (s, 2H), 3.81 (s, 6H). ^{13}C NMR (101 MHz, $CDCl_3$) δ 173.06, 164.18, 155.28, 150.73, 144.92, 131.60, 131.28, 130.58, 130.27, 128.49, 128.29, 127.97, 127.60, 125.76, 122.57, 117.27, 115.93, 110.91, 84.56, 54.36, 43.36. HRMS m/z (EI-TOF): calcd for $C_{25}H_{23}N_3O_4$ (M^+): 429.1689; found 429.1689.

***N*-(2-((4,6-dimethoxypyrimidin-2-yl)oxy)benzyl)-2'-fluoro-[1,1'-biphenyl]-2-amine (Ifa)**

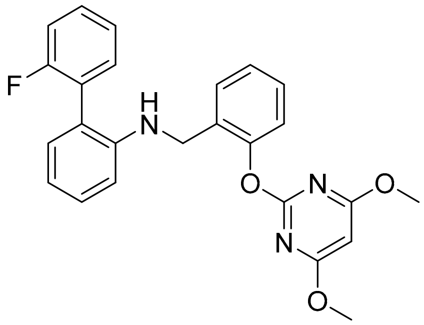

The title compound **Ifa** was prepared from (2-fluorophenyl)boronic acid (**6fa**) according to general procedure B. **Ifa** was obtained as light yellow solid(187 mg, yield 87%; mp: 101.2-102.4°C). ^{1}H NMR (400 MHz, $CDCl_3$) δ 7.43 (dd, *J* = 7.5, 1.0 Hz, 1H), 7.40 – 7.31 (m, 2H), 7.27 (td, *J* = 7.7, 1.5 Hz, 1H), 7.24 – 7.05 (m, 6H), 6.76 (dd, *J* = 7.4, 0.7 Hz, 1H), 6.62 (d, *J* = 8.1 Hz, 1H), 5.77 (s, 1H), 4.36 (s, 2H), 3.78 (s, 6H). ^{13}C NMR (101 MHz, $CDCl_3$) δ 173.02, 164.19, 160.11 (d, $J_{C\text{-}F}$ = 247.0 Hz), 150.78, 145.13, 132.26 (d, $J_{C\text{-}F}$ = 3.5 Hz), 131.51, 130.79, 129.58 (d, $J_{C\text{-}F}$ = 8.0 Hz), 129.33, 128.19, 127.95, 126.48 (d, $J_{C\text{-}F}$ = 16.5 Hz), 125.79, 124.72 (d, $J_{C\text{-}F}$ = 3.6 Hz), 122.54, 121.35, 117.10, 116.23 (d, $J_{C\text{-}F}$ = 22.3 Hz), 111.11, 84.76, 54.27, 43.08. ^{19}F NMR (376 MHz, $CDCl_3$) δ -113.73. HRMS m/z (EI-TOF): calcd for $C_{25}H_{22}N_3O_3F$ (M^+): 431.1645; found.

***N*-(2-((4,6-dimethoxypyrimidin-2-yl)oxy)benzyl)-4'-fluoro-[1,1'-biphenyl]-2-amine (Ifc)**

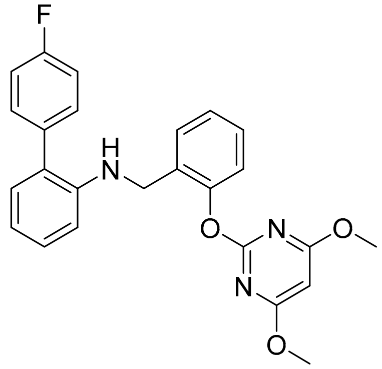

The title compound **Ifc** was prepared from (4-fluorophenyl)boronic acid (**6fc**) according to general procedure B. **Ifc** was obtained as light yellow solid (201 mg, yield 91%; mp: 89.9-91.9°C). ^{1}H NMR (400 MHz, $CDCl_3$) δ 7.45 – 7.34 (m, 3H), 7.29 (td, *J* = 7.8, 1.5 Hz, 1H), 7.23 – 7.08 (m, 5H), 7.05 (dd, *J* = 7.4, 1.5 Hz, 1H), 6.75 (dd, *J* = 7.4, 0.7 Hz, 1H), 6.65 (d, *J* = 8.1 Hz, 1H), 5.78 (s, 1H), 4.34 (s, 2H), 3.77 (s, 6H). ^{13}C NMR (101 MHz, $CDCl_3$) δ 172.98, 162.10 (d, J_{C-F} = 246.2 Hz), 160.87, 150.94, 144.79, 135.28 (d, J_{C-F} = 3.3 Hz), 131.41, 131.07 (d, J_{C-F} = 8.0 Hz), 130.27, 128.79, 128.57, 128.09, 126.71, 125.69, 122.73, 117.29, 115.87 (d, J_{C-F} = 21.2 Hz), 111.05, 84.77, 54.20, 43.46. ^{19}F NMR (376 MHz, $CDCl_3$) δ -115.12. HRMS m/z (EI-TOF): calcd for $C_{25}H_{22}N_3O_3F$ (M^+): 431.1645; found 431.1647.

2'-chloro-*N*-(2-((4,6-dimethoxypyrimidin-2-yl)oxy)benzyl)-[1,1'-biphenyl]-2-amine (Iga)

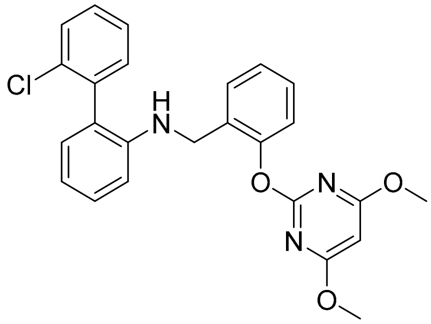

The title compound **Iga** was prepared from (2-chlorophenyl)boronic acid (**6ga**) according to general procedure B. **Iga** was obtained as light yellow solid (186 mg, yield 83%; mp: 126.2-127.2°C). ^{1}H NMR (400 MHz, $CDCl_3$) δ 7.53 – 7.47 (m, 1H), 7.44 (d, *J* = 7.5 Hz, 1H), 7.35 – 7.23 (m, 4H), 7.17 (t, *J* = 7.7 Hz, 2H), 7.11 (d, *J* = 8.0 Hz, 1H), 7.02 (dd, *J* = 7.4, 1.3 Hz, 1H), 6.76 (td, *J* = 7.4, 0.6 Hz, 1H), 6.62 (d, *J* = 8.2 Hz, 1H), 5.78 (s, 1H), 4.35 (s, 2H), 3.78 (s, 6H). ^{13}C NMR (101 MHz, $CDCl_3$) δ 173.01, 164.20, 150.73, 144.84, 137.79, 134.35, 132.17, 131.54, 130.14, 130.05, 129.21, 128.19, 127.92, 127.37, 125.76, 125.14, 122.51, 116.95, 110.97, 84.77, 54.26, 43.05. HRMS m/z (ESI-TOF): calcd for $C_{25}H_{23}N_3O_3Cl$ $(M+H)^+$: 448.1422; found 448.1421.

4'-chloro-*N*-(2-((4,6-dimethoxypyrimidin-2-yl)oxy)benzyl)-[1,1'-biphenyl]-2-amine (Igc)

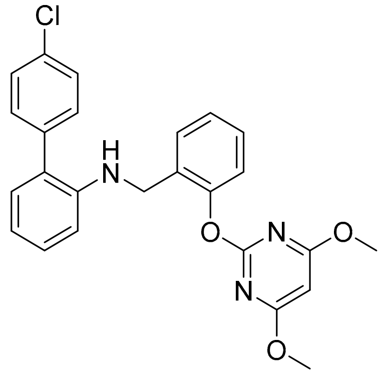

The title compound **Igc** was prepared from (4-chlorophenyl)boronic acid (**6gc**) according to general procedure B. **Igc** was obtained

as light yellow solid (196 mg, yield 89%; mp: 105.1-106.2°C). ^{1}H NMR (400 MHz, $CDCl_3$) δ 7.41 – 7.27 (m, 6H), 7.22 – 7.08 (m, 3H), 7.03 (dd, J = 7.5, 1.5 Hz, 1H), 6.75 (t, J = 7.4 Hz, 1H), 6.66 (d, J = 8.1 Hz, 1H), 5.76 (s, 1H), 4.32 (s, 2H), 3.75 (s, 6H). ^{13}C NMR (101 MHz, $CDCl_3$) δ 172.98, 164.18, 150.99, 144.55, 137.81, 133.22, 131.24, 130.78, 130.19, 129.17, 128.99, 128.71, 128.18, 126.52, 125.71, 122.78, 117.47, 111.24, 84.80, 54.23, 43.57. HRMS m/z (EI-TOF): calcd for $C_{25}H_{22}N_3O_3Cl$ (M^+): 447.1350; found 447.1350.

2'-bromo-*N*-(2-((4,6-dimethoxypyrimidin-2-yl)oxy)benzyl)-[1,1'-biphenyl]-2-amine (Iha)

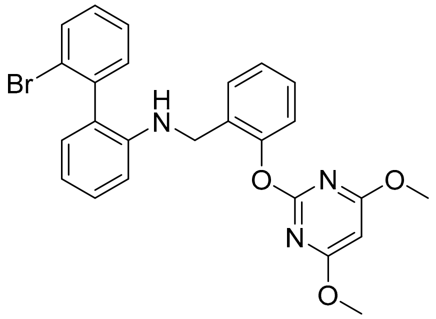

The title compound **Iha** was prepared from (2-bromophenyl)boronic acid (**6ha**) according to general procedure B. **Iha** was obtained as colorless oil (217 mg, yield 87%; mp: 39.5-42.1°C). ^{1}H NMR (400 MHz, $CDCl_3$) δ 7.69 (d, J = 7.7 Hz, 1H), 7.45 (d, J = 6.9 Hz, 1H), 7.38 (t, J = 7.2 Hz, 1H), 7.33 – 7.20 (m, 3H), 7.17 (t, J = 7.6 Hz, 2H), 7.11 (d, J = 7.9 Hz, 1H), 7.00 (dd, J = 7.4, 1.4 Hz, 1H), 6.76 (t, J = 7.3 Hz, 1H), 6.62 (d, J = 8.1 Hz, 1H), 5.78 (s, 1H), 4.35 (s, 2H), 3.78 (s, 6H). ^{13}C NMR (101 MHz, $CDCl_3$) δ 173.06, 164.22, 150.85, 144.33, 139.81, 133.31, 132.10, 131.31, 130.09, 129.45, 129.27, 128.44, 128.03, 128.02, 127.30, 125.77, 124.80, 122.53, 117.37, 111.48, 84.86, 54.30, 43.39. HRMS m/z (EI-TOF): calcd for $C_{25}H_{22}N_3O_3Br$ (M^+): 491.0845; found 491.0847.

***N*-(2-((4,6-dimethoxypyrimidin-2-yl)oxy)benzyl)-2'-(trifluoromethyl)-[1,1'-biphenyl]-2-amine (Iia)**

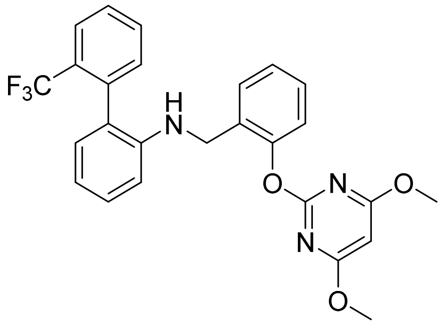

The title compound **Iia** was prepared from (2-(trifluoromethyl)phenyl)boronic acid (**6ia**) according to general procedure B. **Iia** was obtained as white solid (180 mg, yield 74%; mp: 150.6-151.7°C). ^{1}H NMR (400 MHz, $CDCl_3$) δ 7.79 (d, J = 7.8 Hz, 1H), 7.58 (t, J = 7.4 Hz, 1H), 7.48 (t, J = 7.6 Hz, 1H), 7.34 (d, J = 7.5 Hz, 2H), 7.26 (td, J = 7.7, 1.5 Hz, 1H), 7.20 – 7.12 (m, 2H), 7.09 (d, J = 8.0 Hz, 1H), 6.99 (d, J = 7.4 Hz, 1H), 6.73 (td, J = 7.4, 0.7 Hz, 1H), 6.59 (d, J = 8.1 Hz, 1H), 5.77 (s, 1H), 4.31 (s, 2H), 3.77 (s, 6H). ^{13}C NMR (101 MHz, $CDCl_3$) δ 173.02, 164.21, 150.73, 144.97, 137.86, 132.77, 132.10, 131.49, 130.14 (d, J_{C-F} = 1.0 Hz), 130.08 (q, J_{C-F} = 29.9 Hz), 129.19, 128.10, 128.03, 127.95, 126.78 (q, J_{C-F} = 5.3 Hz), 125.78, 124.59, 123.99 (q, J_{C-F} = 274.2 Hz) 122.53, 116.50, 110.89, 84.77, 54.27, 43.11. ^{19}F NMR (376 MHz, $CDCl_3$) δ -59.30. HRMS m/z (ESI-TOF): calcd for $C_{26}H_{23}N_3O_3F_3$ $(M+H)^+$: 482.1686; found 482.1689.

***N*-(2-((4,6-dimethoxypyrimidin-2-yl)oxy)benzyl)-4'-(trifluoromethyl)-[1,1'-biphenyl]-2-amine (Iic)**

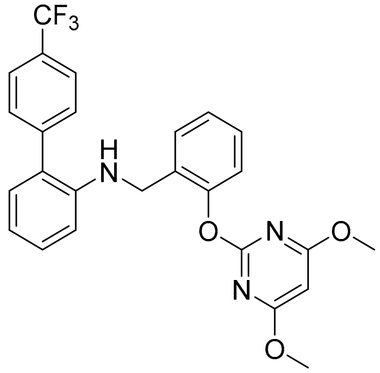

The title compound **Iic** was prepared from (4-(trifluoromethyl)phenyl)boronic acid (**6ic**) according to general procedure B. **Iic** was obtained as white solid (203 mg, yield 83%; mp: 112.2-114.2°C). ^{1}H NMR (400 MHz, $CDCl_3$) δ 7.68 (d, *J* = 8.0 Hz, 2H), 7.54 (d, *J* = 8.0 Hz, 2H), 7.42 (d, *J* = 7.5 Hz, 1H), 7.30 (t, *J* = 7.6 Hz, 1H), 7.24 – 7.10 (m, 3H), 7.05 (dd, *J* = 7.4, 1.1 Hz, 1H), 6.77 (t, *J* = 7.4 Hz, 1H), 6.68 (d, *J* = 8.2 Hz, 1H), 5.77 (s, 1H), 4.35 (s, 2H), 3.75 (s, 6H). ^{13}C NMR (101 MHz, $CDCl_3$) δ 172.99, 164.21, 151.04, 144.59, 143.33, 131.23, 130.23, 129.77, 129.57 (q, $J_{C\text{-}F}$ = 40.9 Hz), 129.36, 128.73, 128.21, 126.15, 125.91 (q, $J_{C\text{-}F}$ = 3.6 Hz), 125.71, 124.29 (q, $J_{C\text{-}F}$ = 272.0 Hz), 122.85, 117.45, 111.28, 84.80, 54.19, 43.54. ^{19}F NMR (376 MHz, $CDCl_3$) δ -62.46. HRMS m/z (EI-TOF): calcd for $C_{26}H_{22}N_3O_3F_3$ (M^+): 481.1613; found 481.1611.

References

1. Meng, J.-P.; Wang, W.-W.; Chen, Y.-L.; Bera, S.; Wu, J. Switchable Solvent-Controlled Divergent Synthesis: An Efficient and Regioselective Approach to Pyrimidine and Dibenzo[b,f][1,4]oxazepine Derivatives. *Org. Chem. Front.* **2020,** *7*, 267-272.

3. Supplementary Tables

Table S1. Herbicidal activities of **Ia**, **IIa** and **IIIa**.[a]

Compd.	Inhibition (post-emergence, %)					Inhibition (pre-emergence, %)				
	EC[b]	DS	AT	CT	Total	EC	DS	AT	CT	Total
**Ia**	60	**85**	**85**	**85**	**315**	30	80	30	30	170
**IIa**	40	55	50	10	155	0	30	0	0	30
**IIIa**	45	80	60	50	235	20	80	45	40	185
Oxyfluorfen	90	98	95	50	333	0	0	0	0	0

[a]Herbicidal activities were tested at rates of 750 g ai/ha and 30 g ai/ha for the tested compounds and the positive control (oxyfluorfen), respectively. [b]Abbreviations: EC, *Echinochloa crusgalli*; DS, *Digitaria sanguinalis*; AT, *Abutilon theophrasti*; CT, *Cassia tora*.

Table S2. Herbicidal activities of **Ia** and **Iba-ic**.[a]

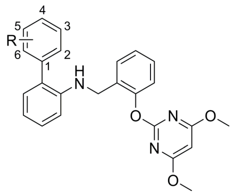

Ia, Iba-ic

Compd.	R	Inhibition (post-emergence, %)					Inhibition (pre-emergence, %)				
		EC [b]	DS	AT	CT	Total	EC	DS	AT	CT	Total
**Ia**	H	60	85	85	85	315	30	80	30	30	170
**Iba**	2-Me	**98**	**100**	45	98	341	60	90	70	35	255
**Ibb**	3-Me	100	85	45	80	310	65	90	75	30	260
**Ibc**	4-Me	100	90	60	80	330	85	87	72	55	299
**Ica**	2-OMe	**100**	**100**	50	88	338	90	92	72	50	304
**Icb**	3-OMe	**100**	**100**	40	85	325	60	90	72	35	257
**Icc**	4-OMe	**100**	**100**	70	88	358	88	90	75	50	303
**Ida**	2-NH_2	100	80	65	20	265	30	87	70	50	237
**Idb**	3-NH_2	100	82	65	90	337	40	92	72	48	252
**Idc**	4-NH_2	100	95	25	88	308	65	90	75	45	275
**Iea**	2-OH	100	80	50	85	315	55	87	68	40	250
**Iec**	4-OH	92	65	35	75	267	65	92	75	40	272
**Ifa**	2-F	92	75	40	82	289	65	87	80	30	262
**Ifc**	4-F	98	90	45	70	303	55	87	70	30	242
**Iga**	2-Cl	100	80	40	80	300	55	87	60	30	232
**Igc**	4-Cl	95	98	55	70	318	60	90	60	40	250
**Iha**	2-Br	**100**	**100**	50	45	295	55	92	65	30	242
**Iia**	2-CF_3	**98**	**100**	60	80	338	60	90	78	30	258
**Iic**	4-CF_3	100	78	45	90	313	80	92	80	45	297

[a]Herbicidal activities were tested at rates of 750 g ai/ha. [b]Abbreviations: EC, *Echinochloa crusgalli*; DS, *Digitaria sanguinalis*; AT, *Abutilon theophrasti*; CT, *Cassia tora.*

Table S3. Calculated binding free energy and energy components of **Ia** and **Iba-ic**.

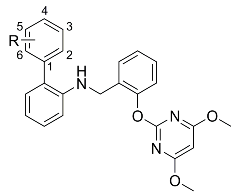

Ia, Iba-ic

Compd.	R	Calculated Energy (kJ/mol)				
		ΔE_{VdW}	ΔE_{ELE}	ΔE_{MM}	ΔG_{SOL}	ΔG_{bind}
**Ia**	-H	-190.013	-24.397	-214.410	89.455	-124.955
**Iba**	2-Me	-205.378	-34.460	-239.838	98.430	**-141.408** [a]
**Ibb**	3-Me	-185.960	-20.703	-206.663	77.665	-128.998
**Ibc**	4-Me	-204.353	-32.622	-236.975	92.798	**-144.177**
**Ica**	2-OMe	-208.265	-13.645	-221.910	76.363	**-145.547**
**Icb**	3-OMe	-210.590	-23.267	-233.857	95.137	**-138.720**
**Icc**	4-OMe	-207.160	-22.282	-229.442	86.167	**-143.275**
**Ida**	2-NH_2	-196.530	-25.322	-221.852	101.108	-120.744
**Idb**	3-NH_2	-200.991	-40.320	-241.311	116.020	-125.291
**Idc**	4-NH_2	-203.915	-31.864	-235.779	108.229	-127.550
**Iea**	2-OH	-187.291	-30.212	-217.503	92.274	-125.229
**Iec**	4-OH	-194.716	-21.136	-215.852	95.472	-120.380
**Ifa**	2-F	-201.466	-14.390	-215.856	80.679	-135.177
**Ifc**	4-F	-198.385	-27.831	-226.216	93.998	-132.218
**Iga**	2-Cl	-192.136	-32.716	-224.852	96.415	-128.437
**Igc**	4-Cl	-204.084	-28.334	-232.418	91.562	**-140.856**
**Iha**	2-Br	-212.315	-38.509	-250.824	110.122	**-140.702**
**Iia**	2-CF_3	-207.124	-39.566	-246.690	106.587	**-140.103**
**Iic**	4-CF_3	-196.017	-21.764	-217.781	87.862	-129.919

[a]Virtual hits are shown in bold font.

4. Supplementary Figure

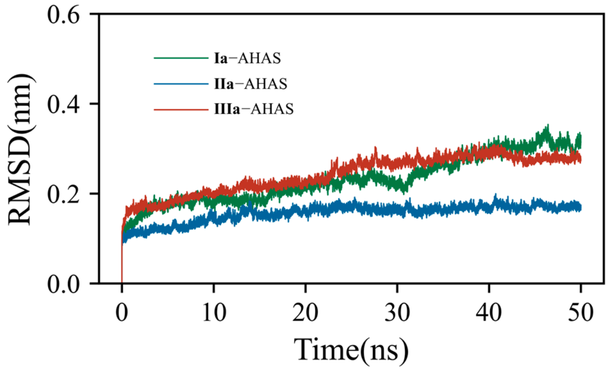

Figure S1. Backbone root mean square deviation (RMSD) of AHAS in the simulation systems.

5. Original NMR Spectra for Ia, IIa, IIIa and Iba-ic

^{1}H-NMR spectrum for **Ia** (400 MHz, $CDCl_3$)

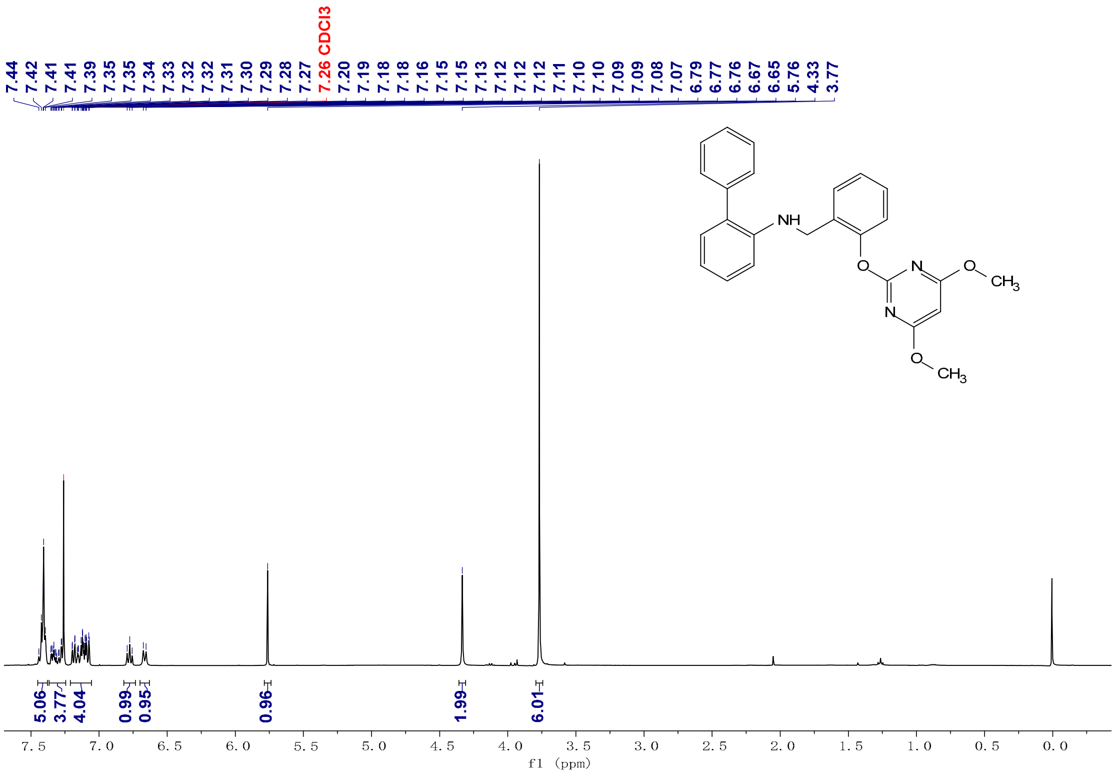

^{13}C-NMR spectrum for **Ia** (101 MHz, $CDCl_3$)

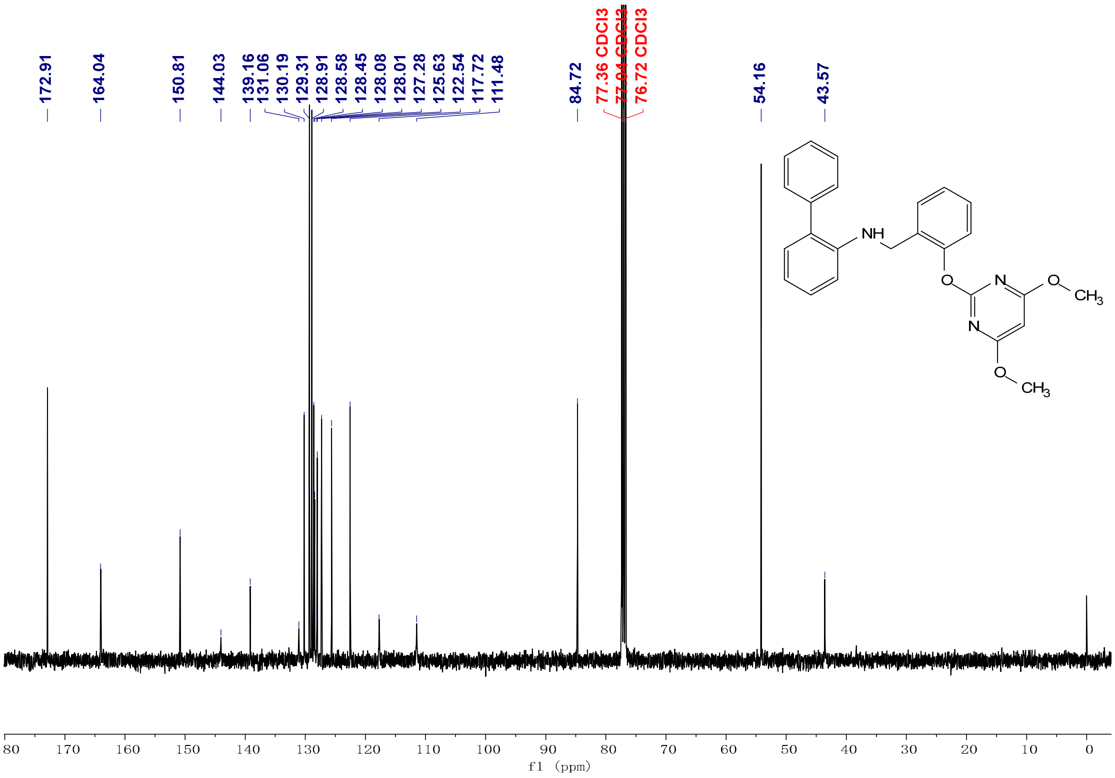

^{1}H-NMR spectrum for **IIa** (400 MHz, $CDCl_3$)

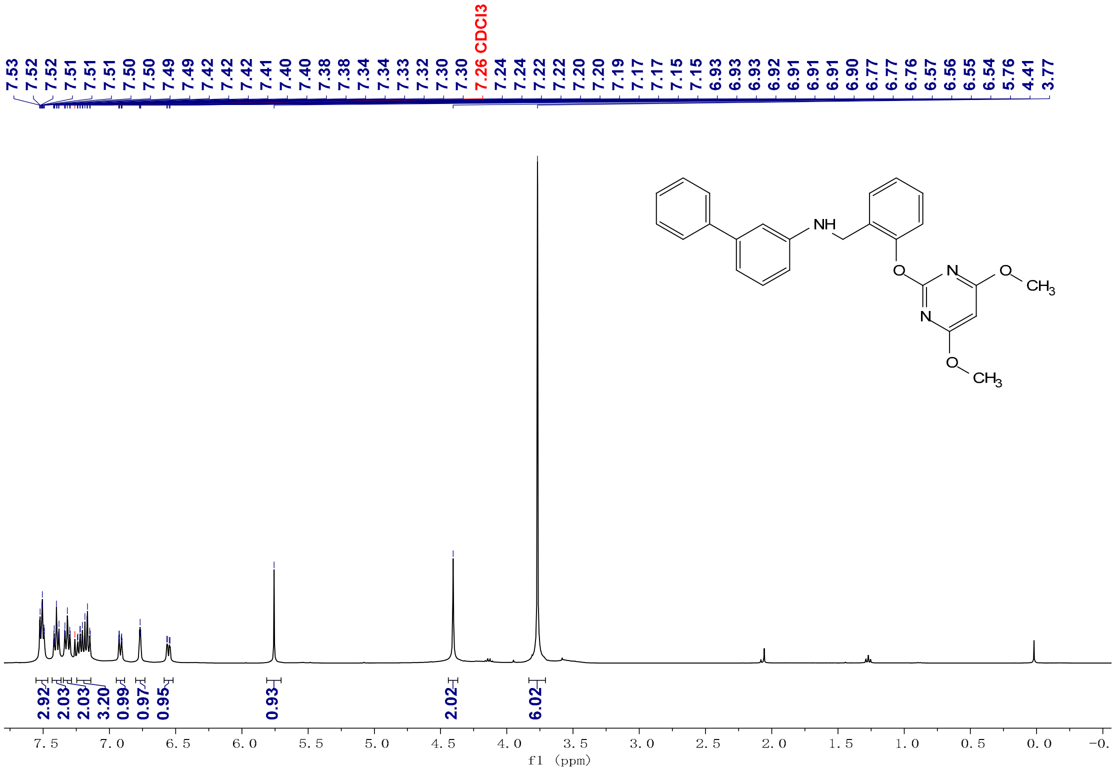

^{13}C-NMR spectrum for **IIa** (101 MHz, $CDCl_3$)

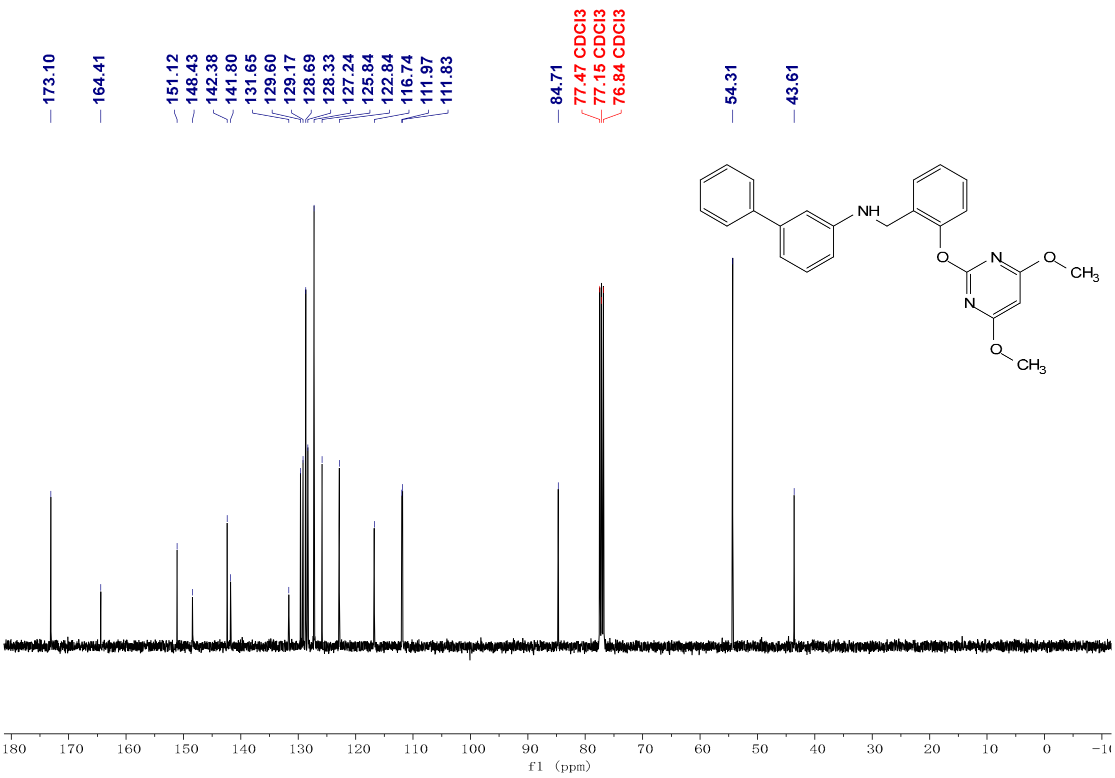

^{1}H-NMR spectrum for **IIIa** (400 MHz, $CDCl_3$)

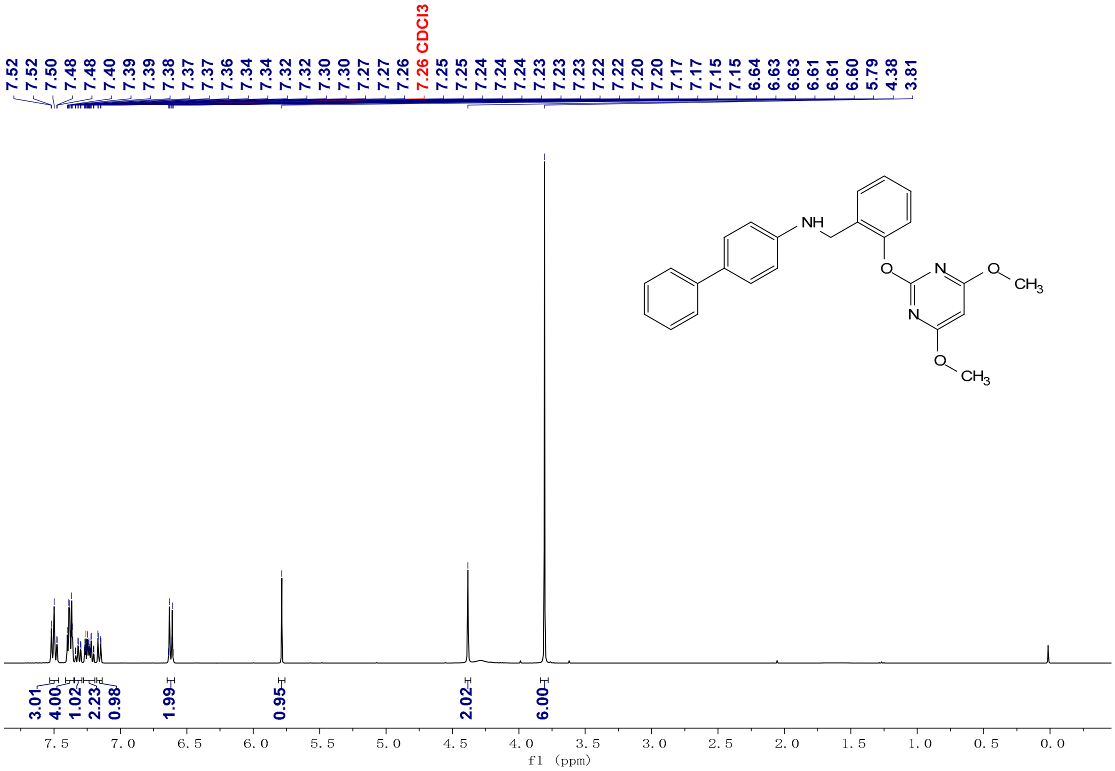

^{13}C-NMR spectrum for **IIIa** (101 MHz, $CDCl_3$)

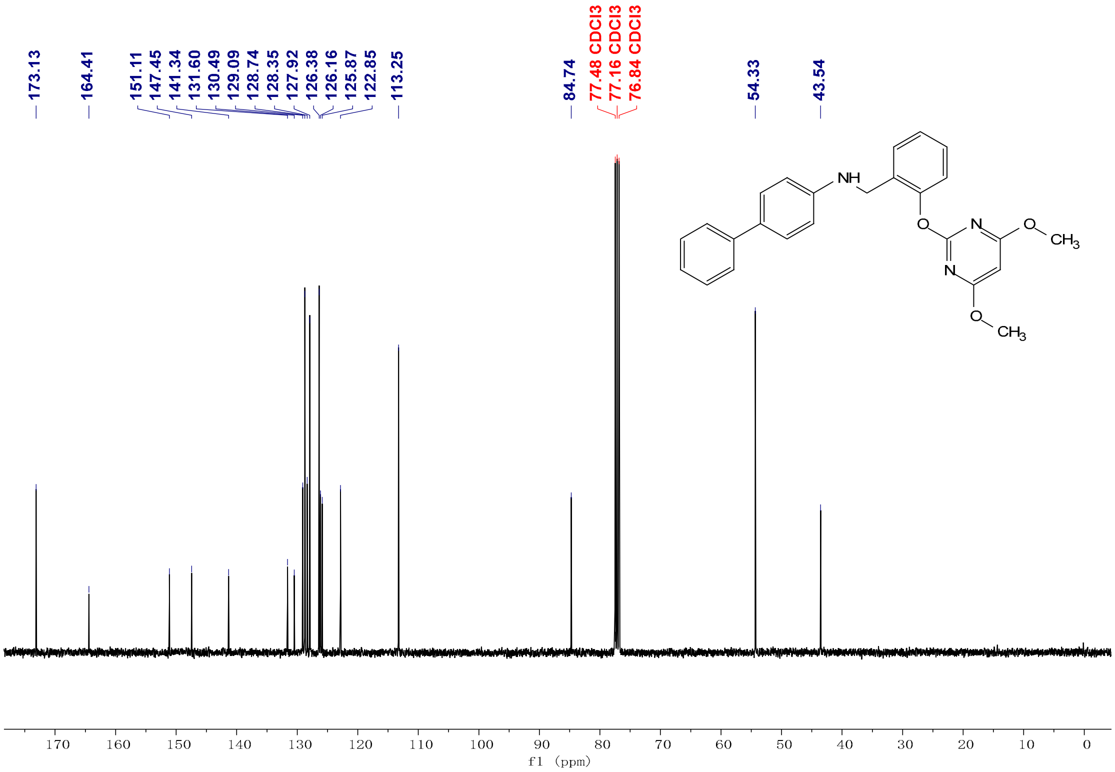

^{1}H-NMR spectrum for **Iba** (400 MHz, $CDCl_3$)

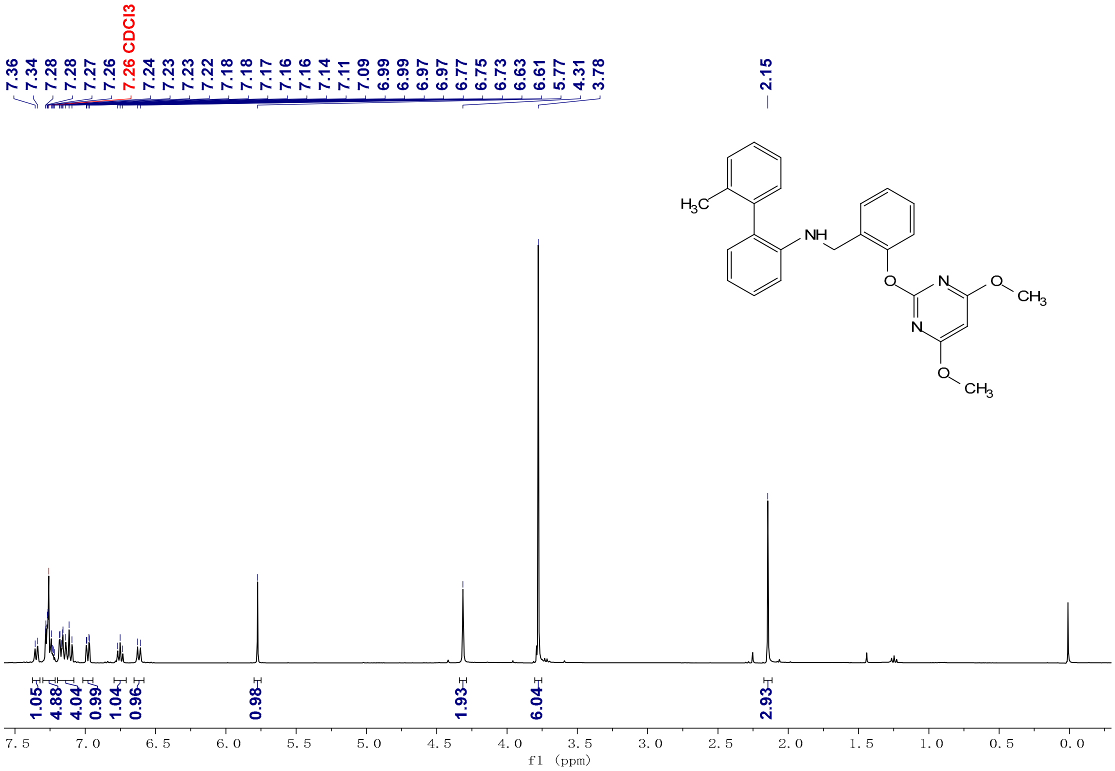

^{13}C-NMR spectrum for **Iba** (101 MHz, $CDCl_3$)

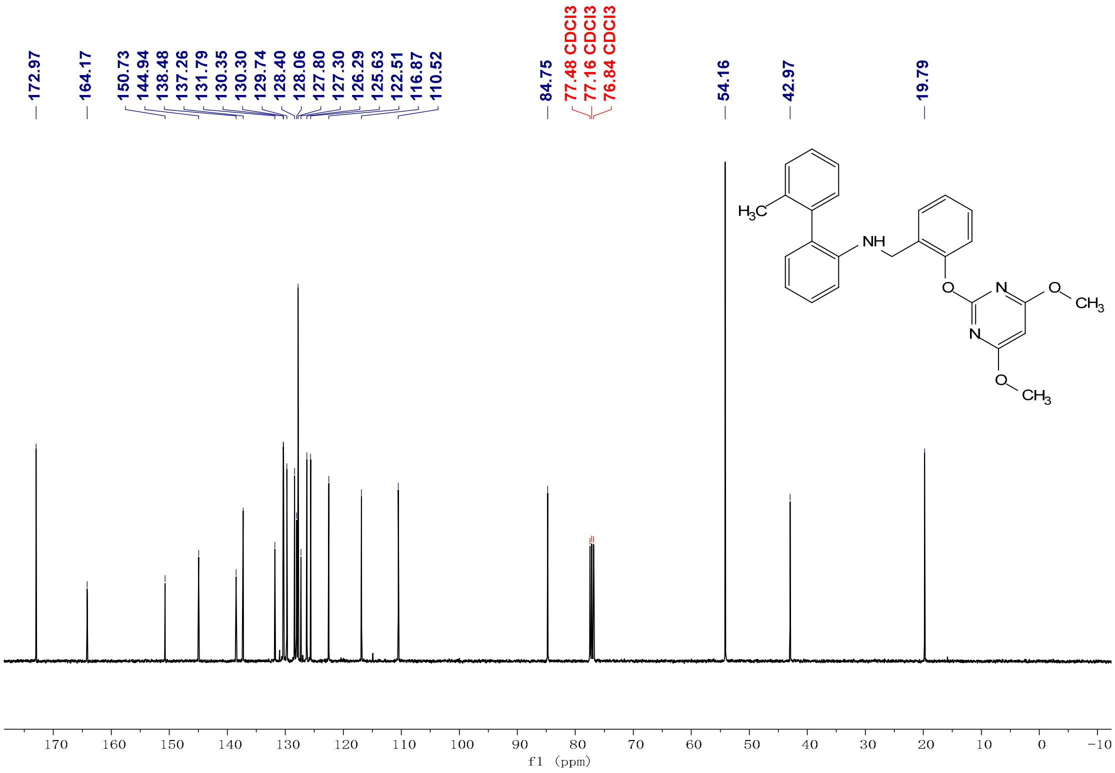

^{1}H-NMR spectrum for **Ibb** (400 MHz, $CDCl_3$)

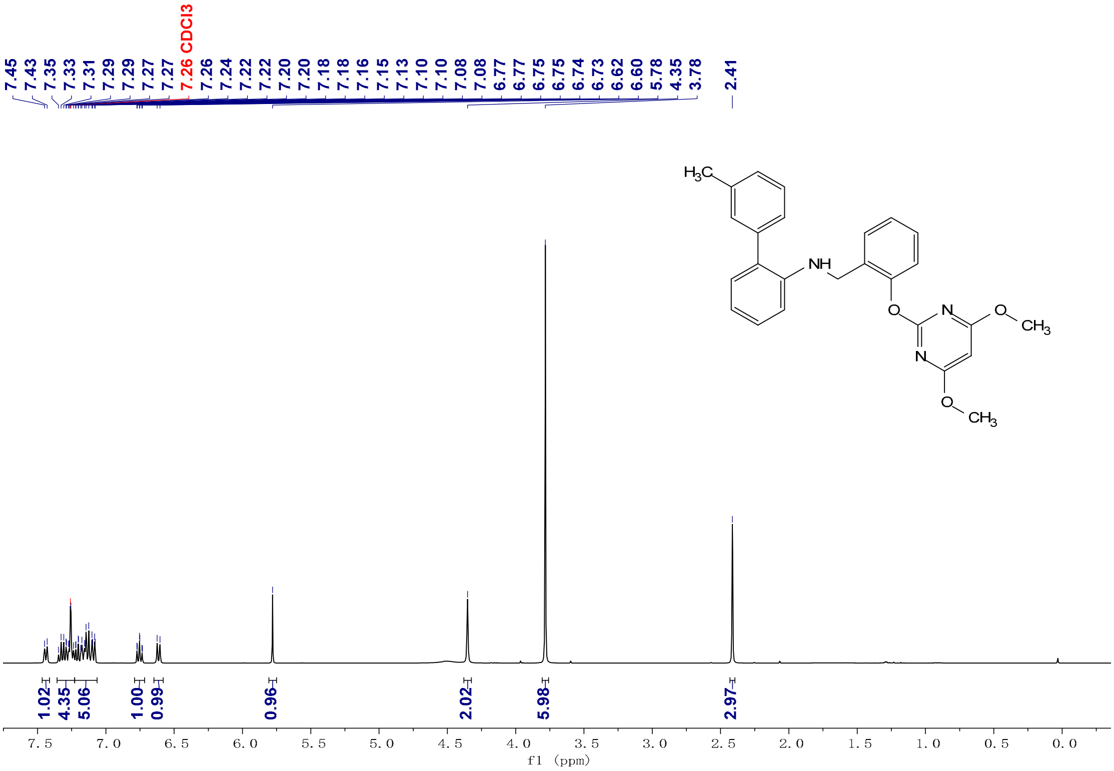

^{13}C-NMR spectrum for **Ibb** (101 MHz, $CDCl_3$)

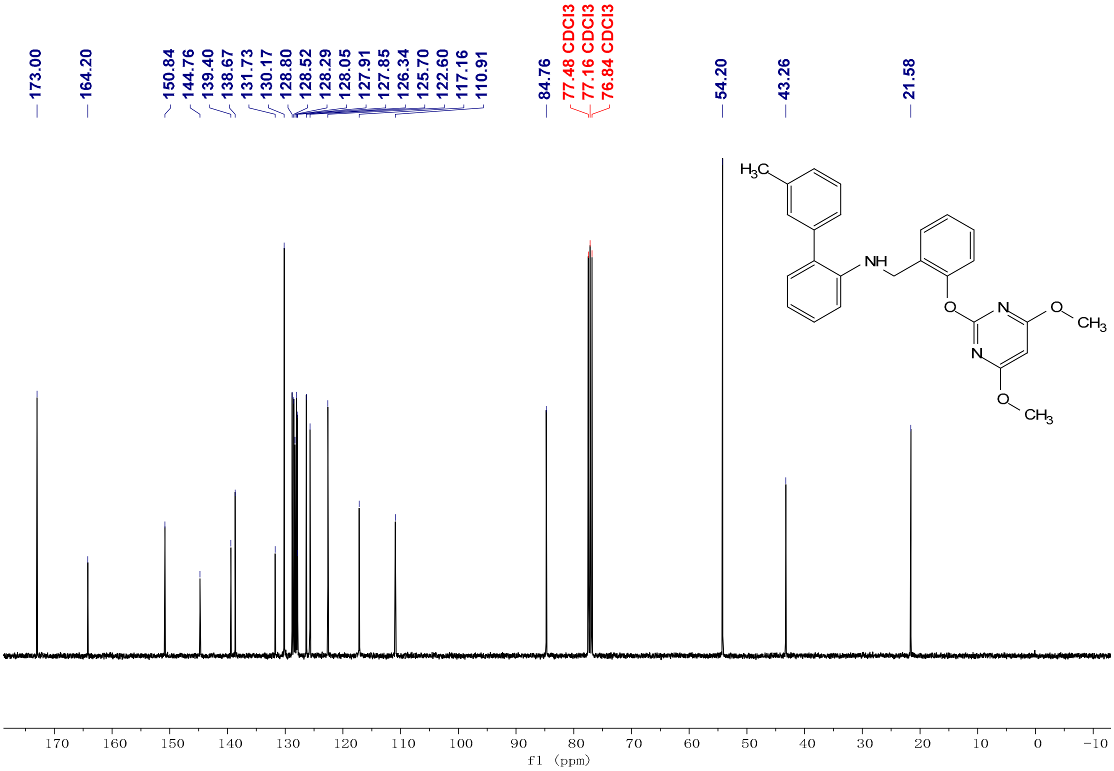

^{1}H-NMR spectrum for **Ibc** (400 MHz, $CDCl_3$)

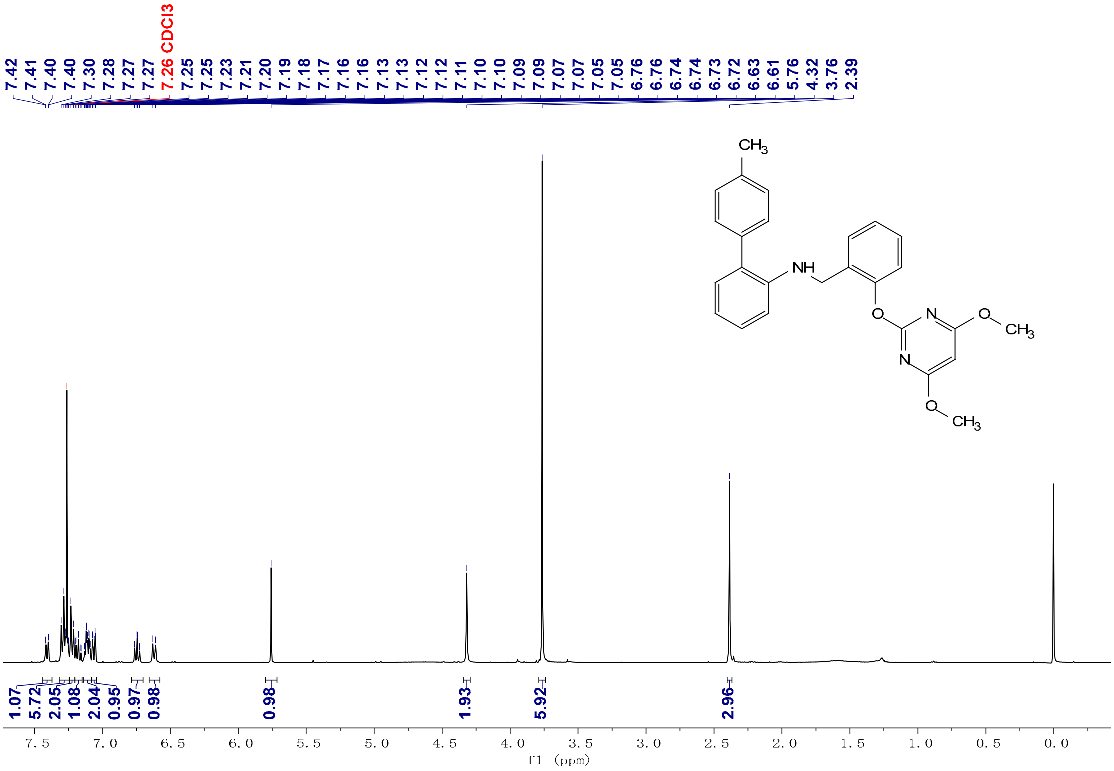

^{13}C-NMR spectrum for **Ibc** (101 MHz, $CDCl_3$)

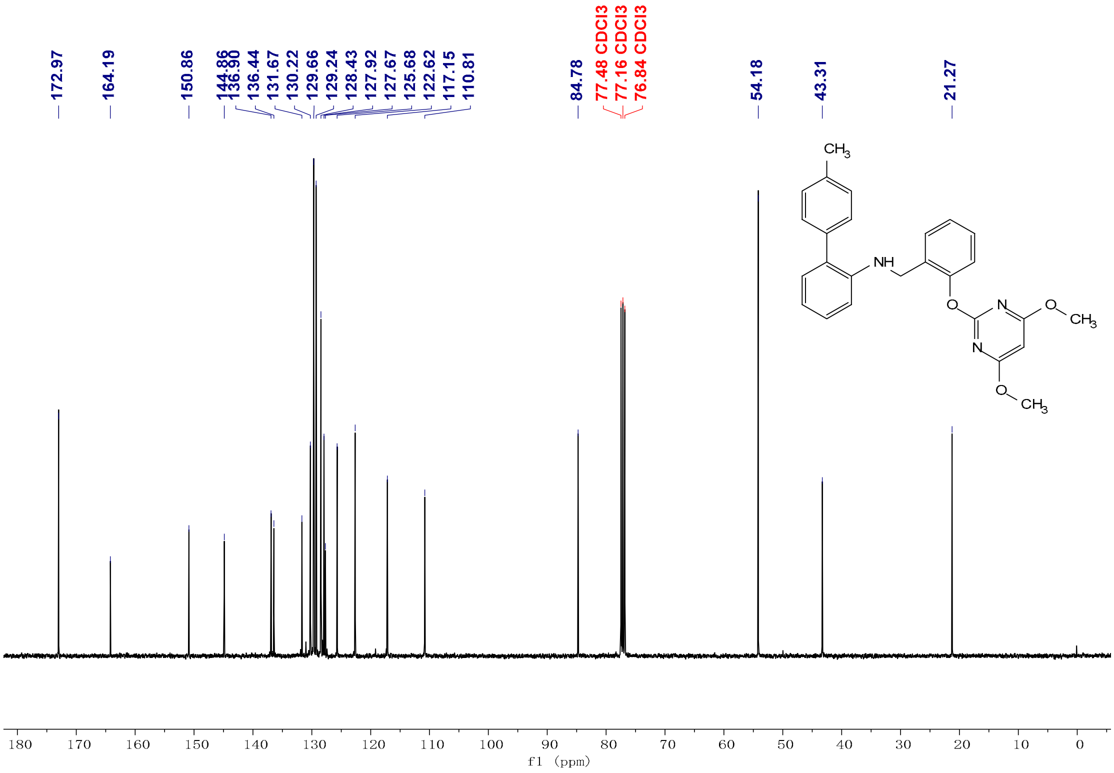

^{1}H-NMR spectrum for **Ica** (400 MHz, $CDCl_3$)

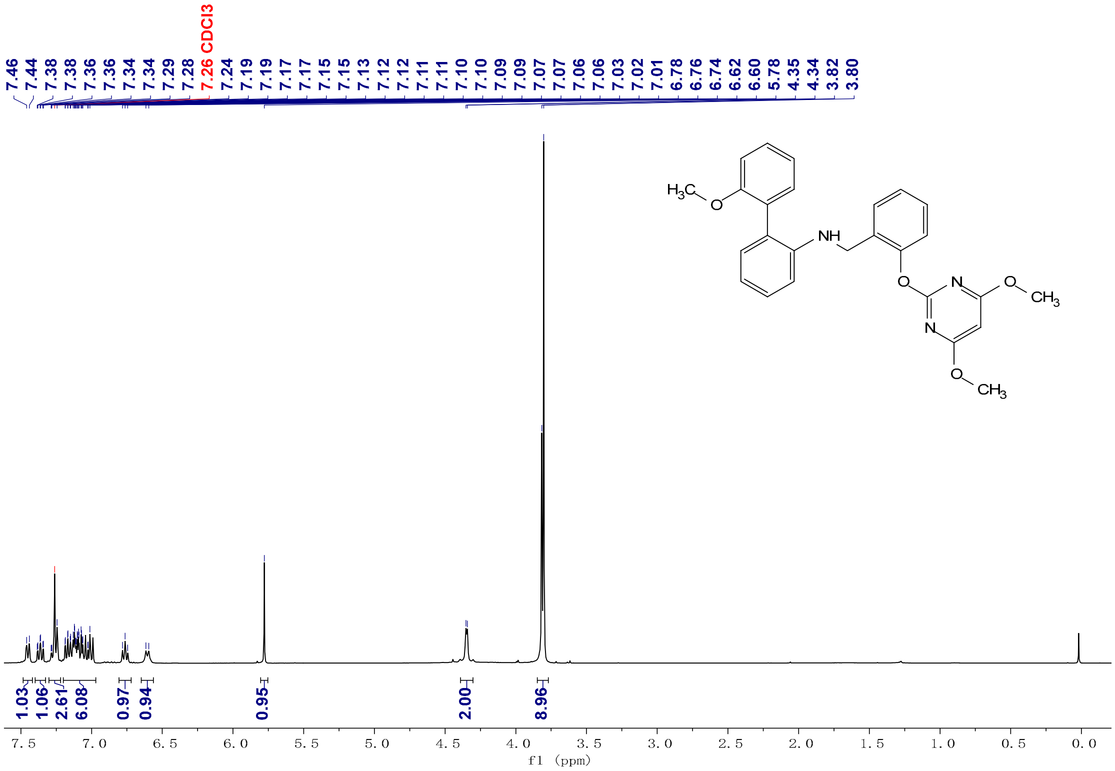

^{13}C-NMR spectrum for **Ica** (101 MHz, $CDCl_3$)

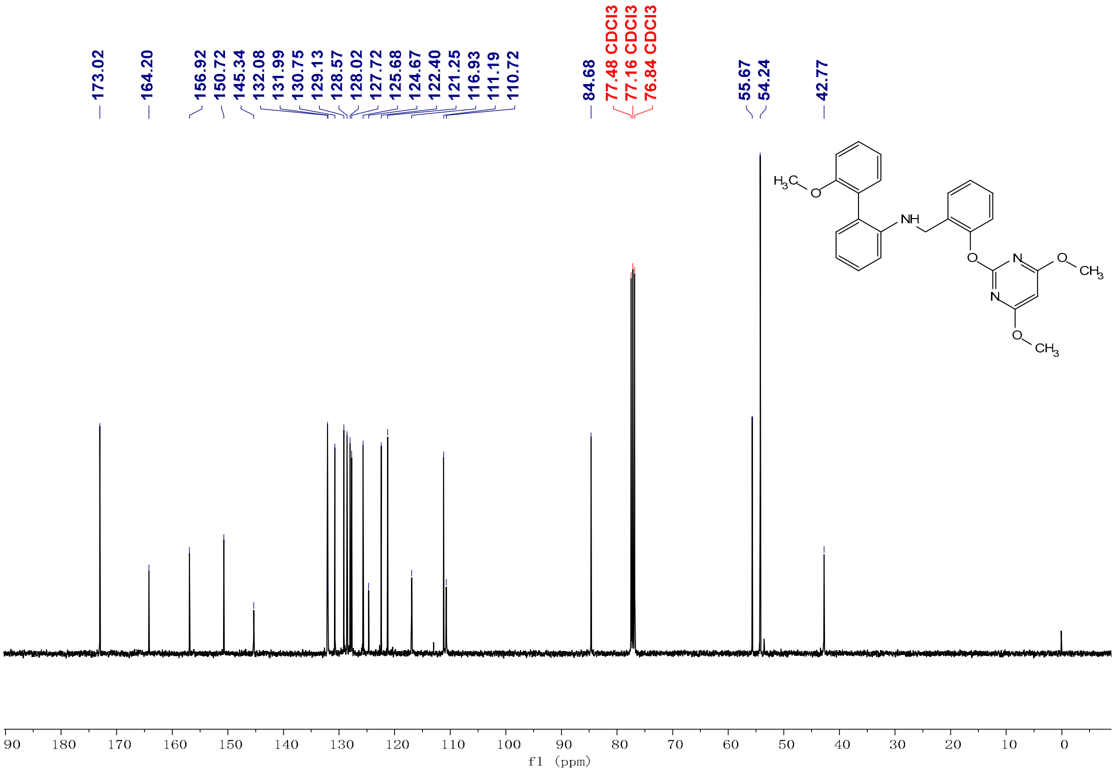

^{1}H-NMR spectrum for **Icb** (400 MHz, $CDCl_3$)

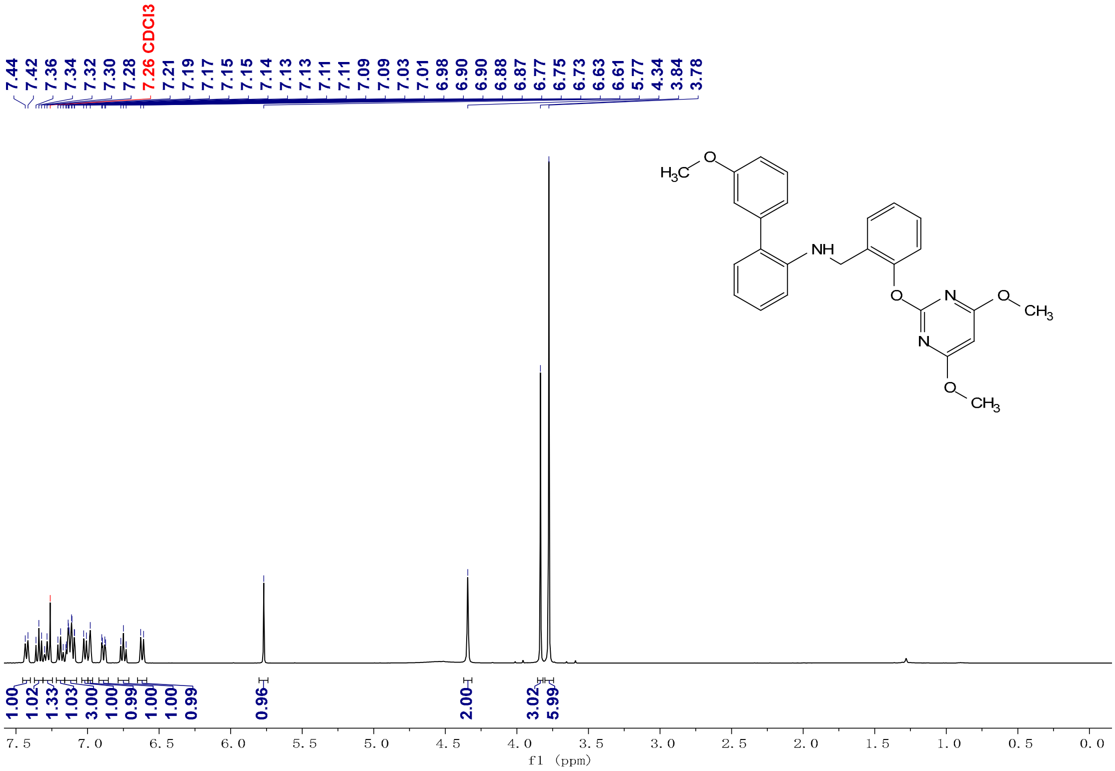

^{13}C-NMR spectrum for **Icb** (101 MHz, $CDCl_3$)

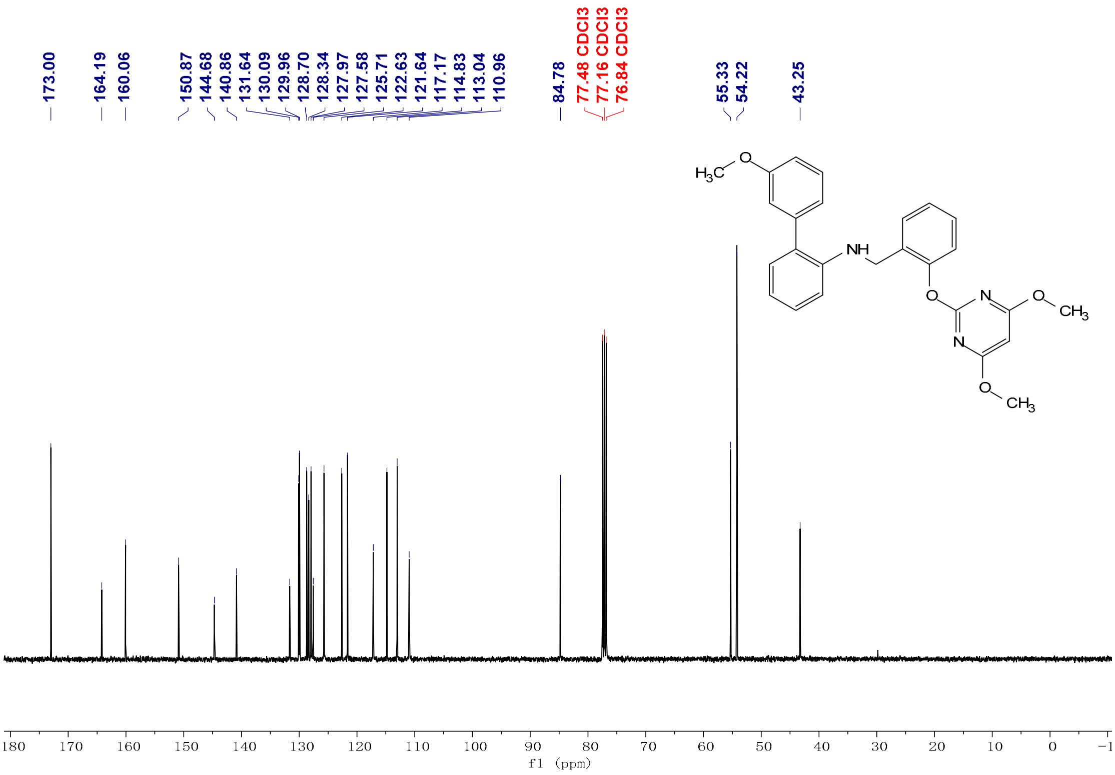

^{1}H-NMR spectrum for **Icc** (400 MHz, $CDCl_3$)

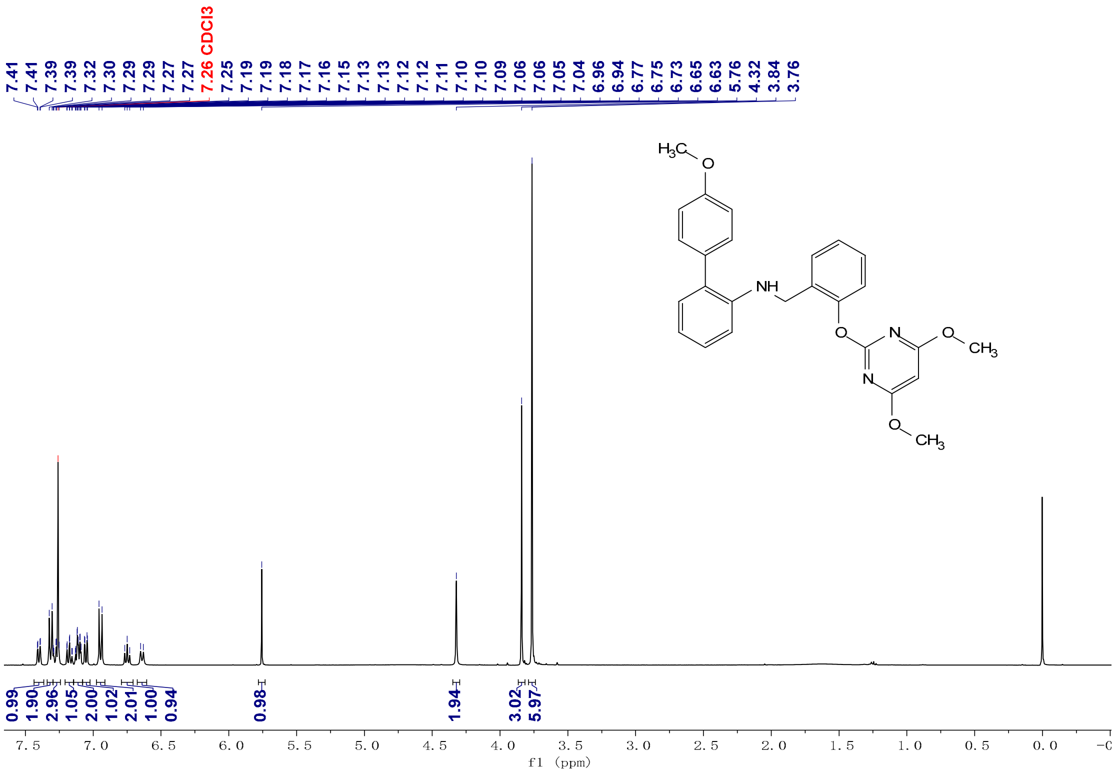

^{13}C-NMR spectrum for **Icc** (101 MHz, $CDCl_3$)

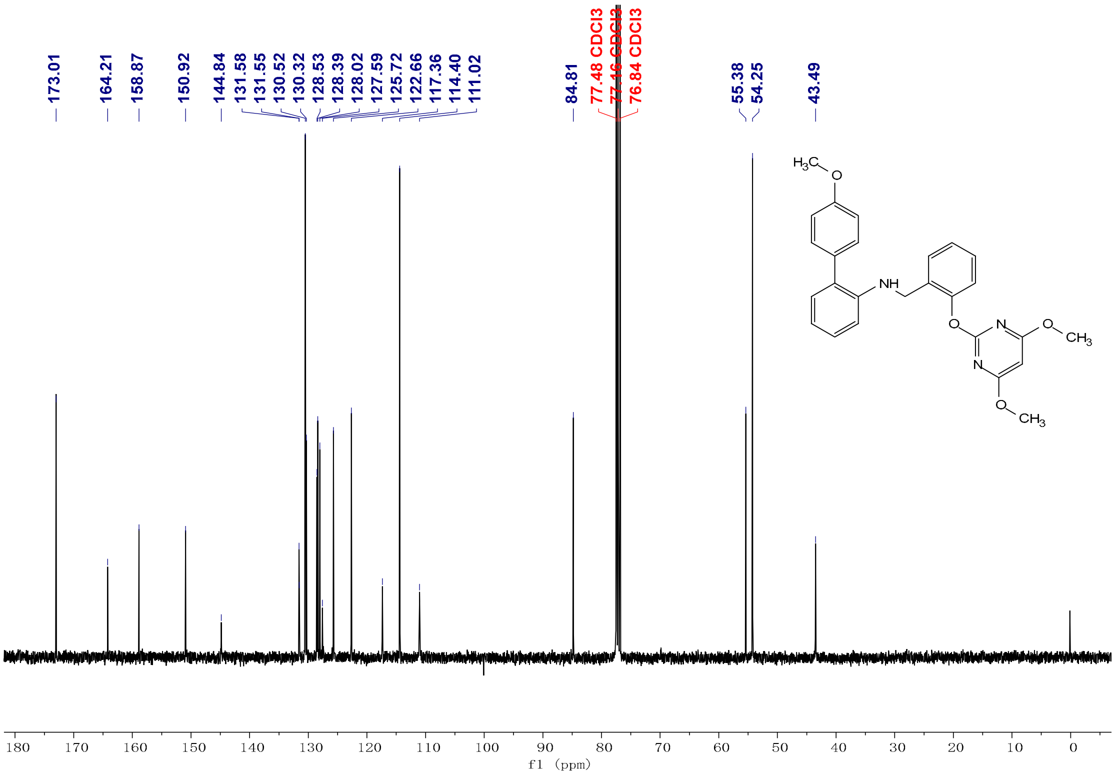

^{1}H-NMR spectrum for **Ida** (400 MHz, $CDCl_3$)

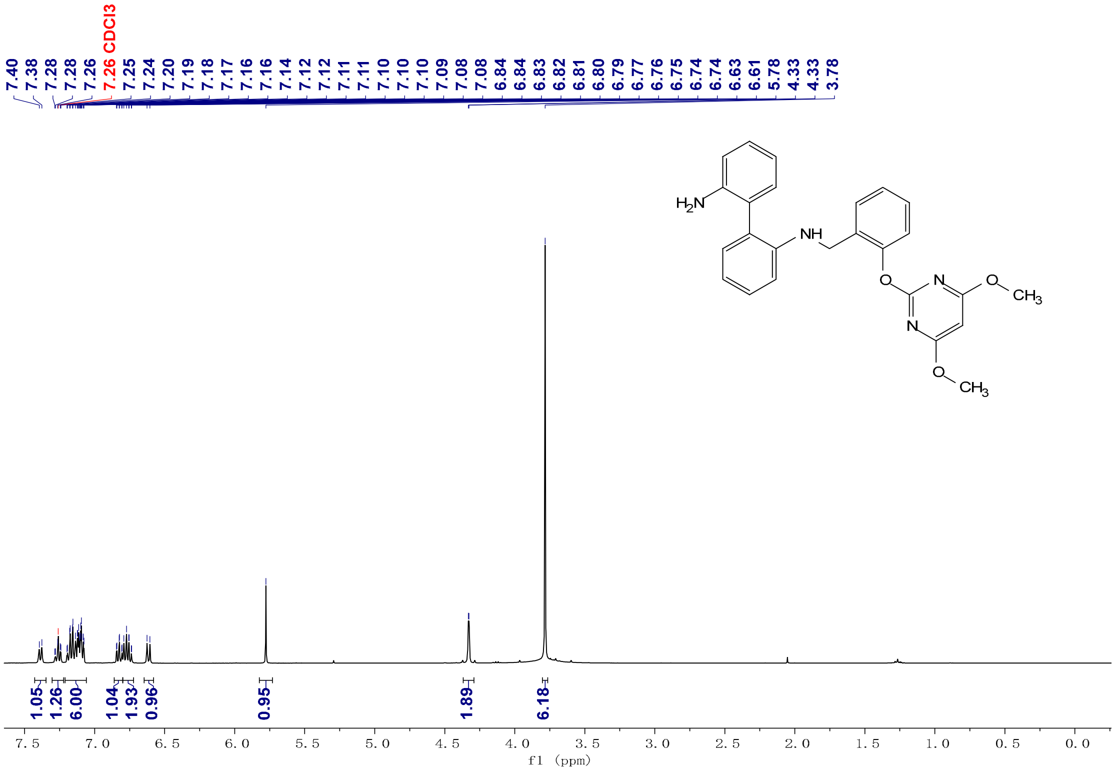

^{13}C-NMR spectrum for **Ida** (101 MHz, $CDCl_3$)

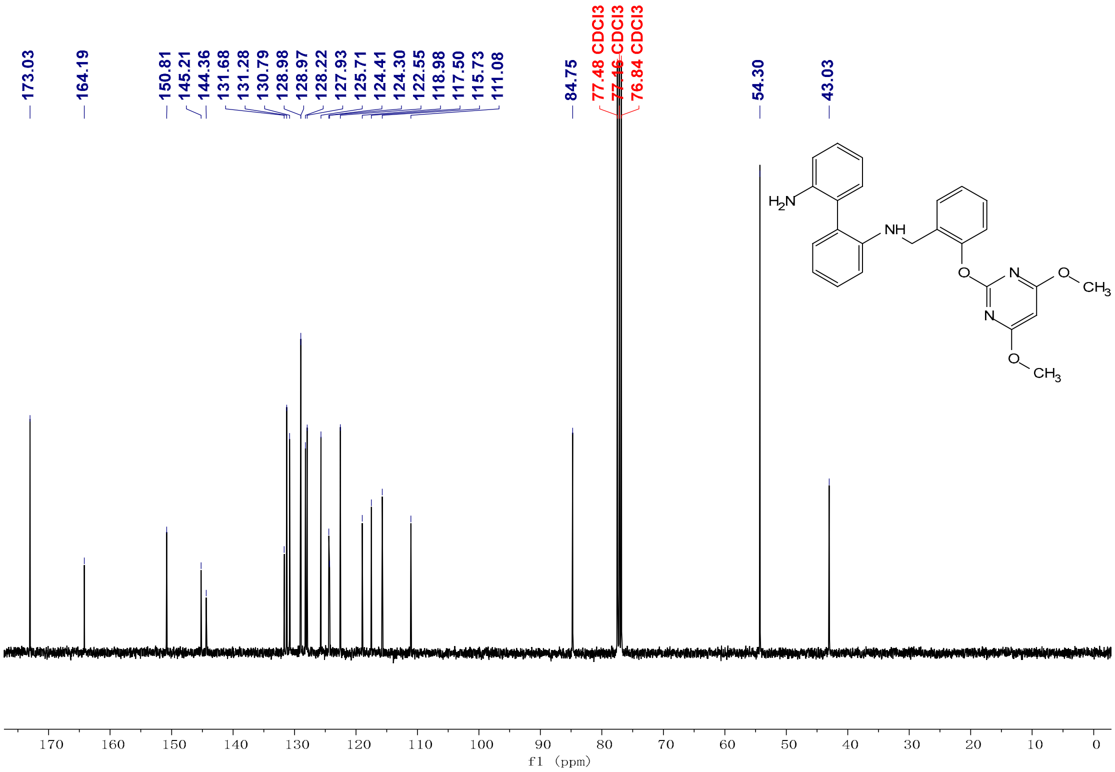

^{1}H-NMR spectrum for **Idb** (400 MHz, $CDCl_3$)

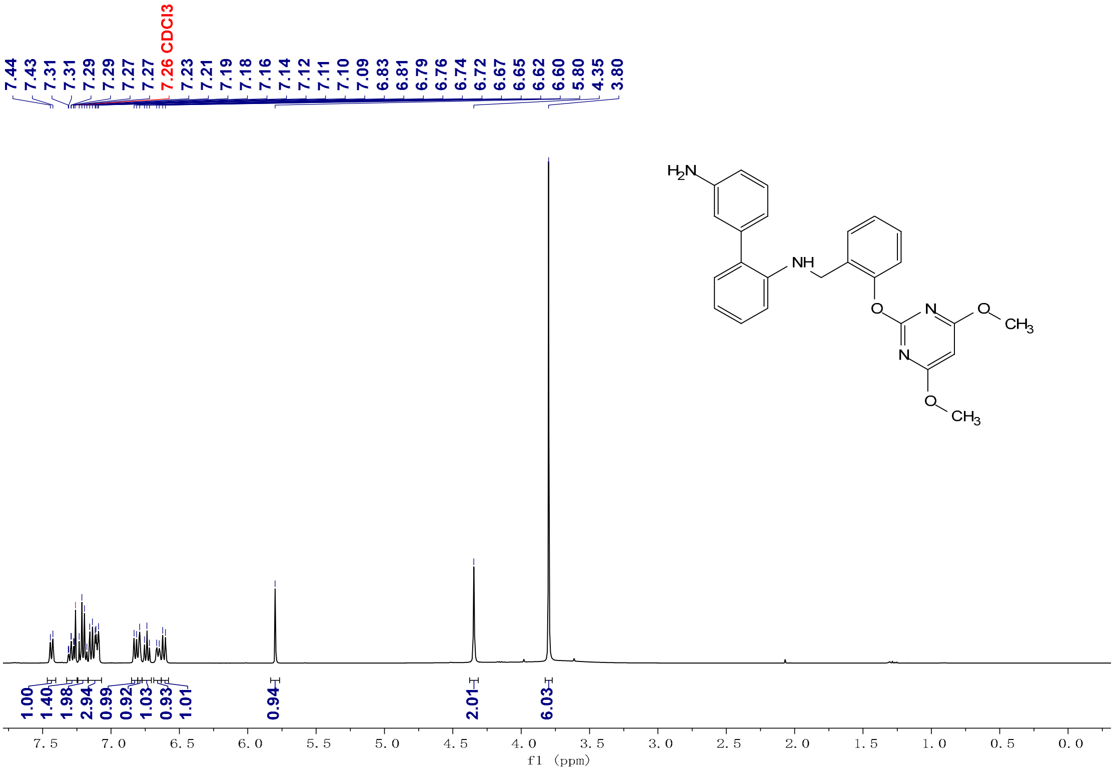

^{13}C-NMR spectrum for **Idb** (101 MHz, $CDCl_3$)

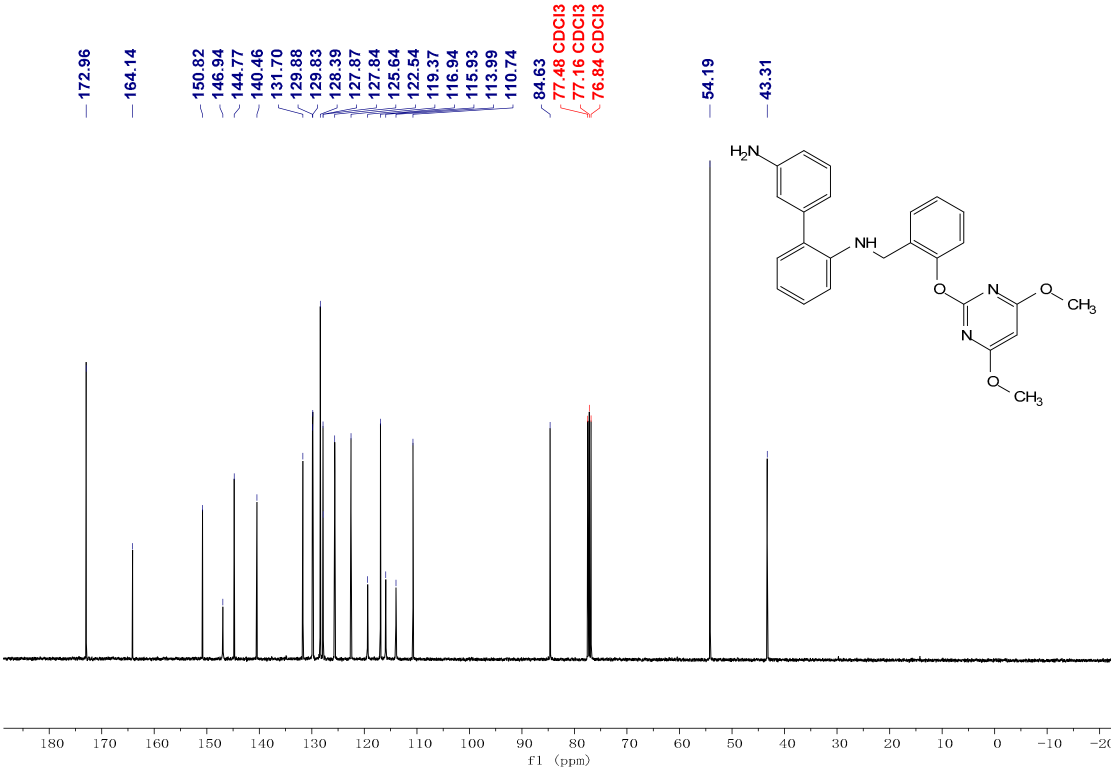

^{1}H-NMR spectrum for **Idc** (400 MHz, $CDCl_3$)

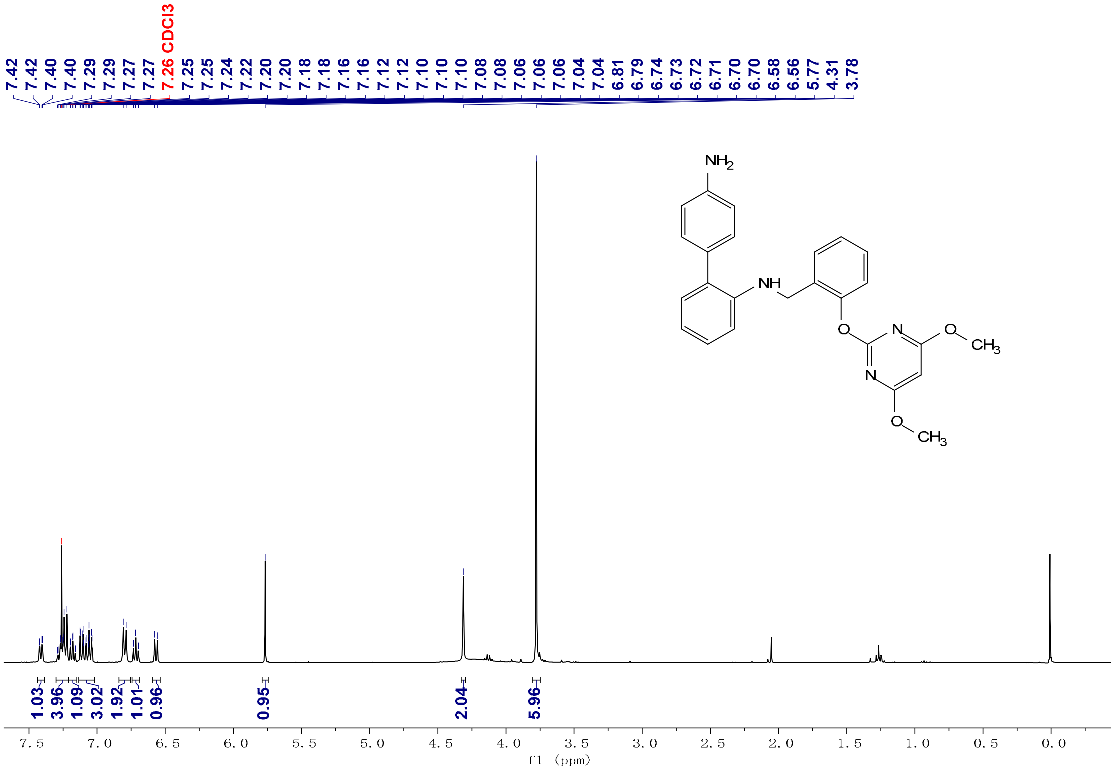

^{13}C-NMR spectrum for **Idc** (101 MHz, $CDCl_3$)

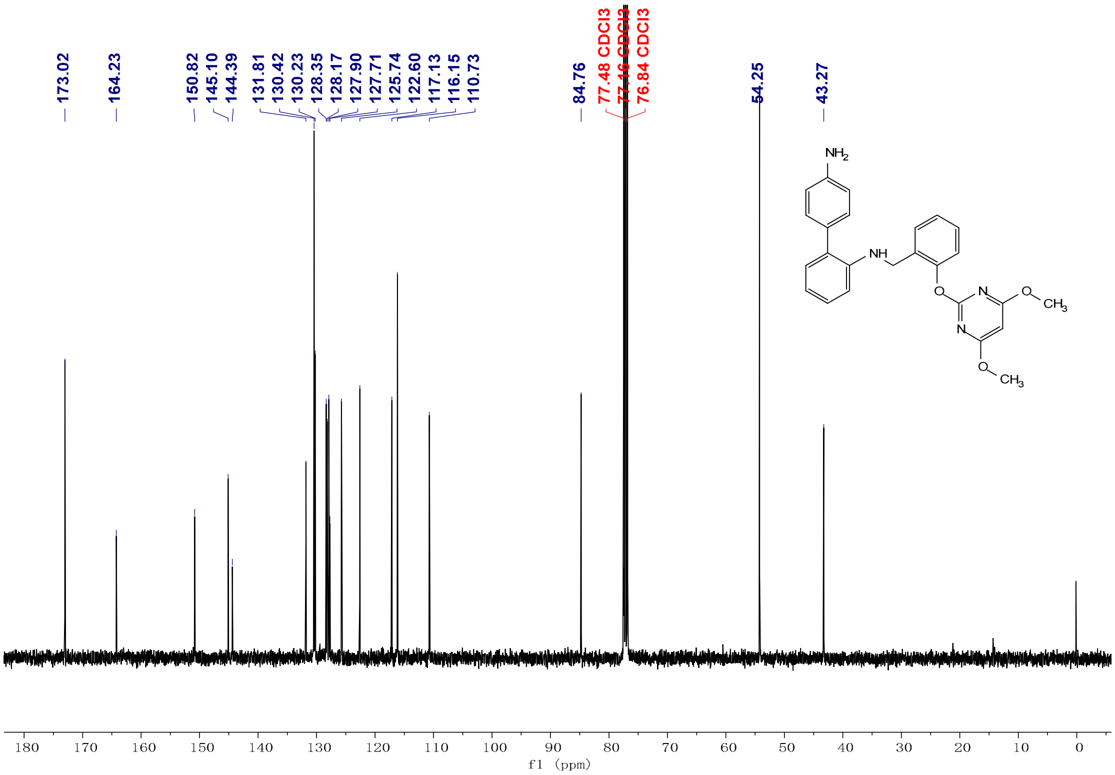

^{1}H-NMR spectrum for **Iea** (400 MHz, $CDCl_3$)

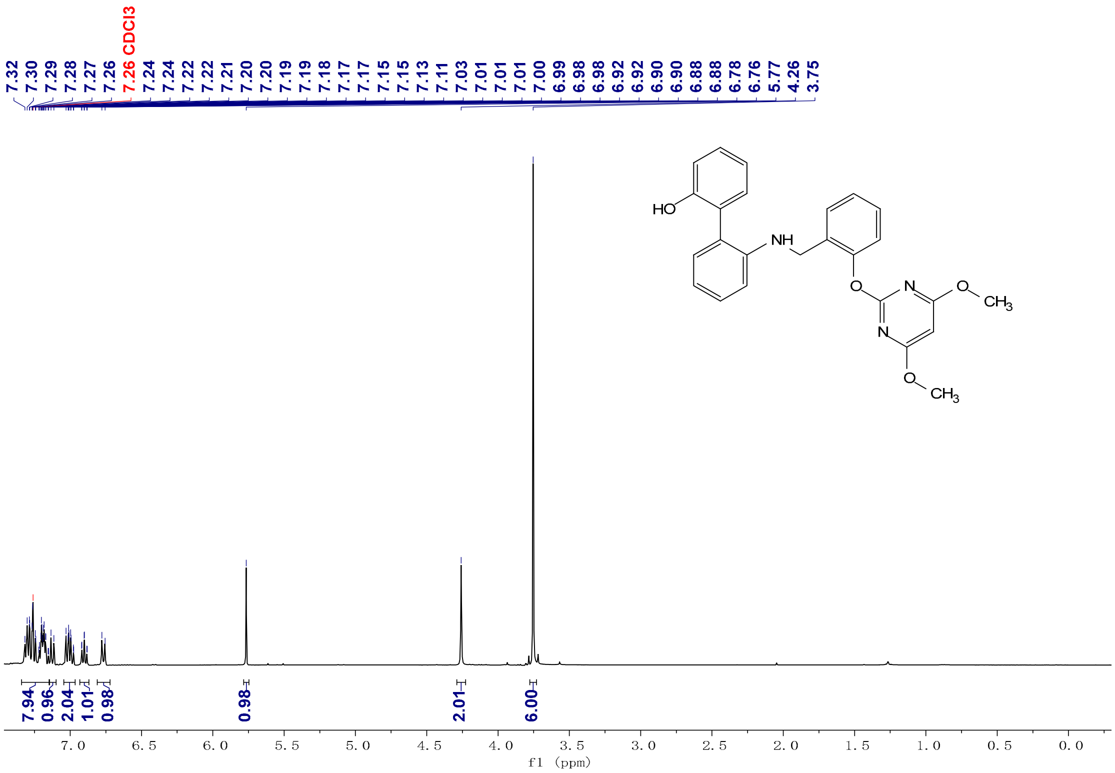

^{13}C-NMR spectrum for **Iea** (101 MHz, $CDCl_3$)

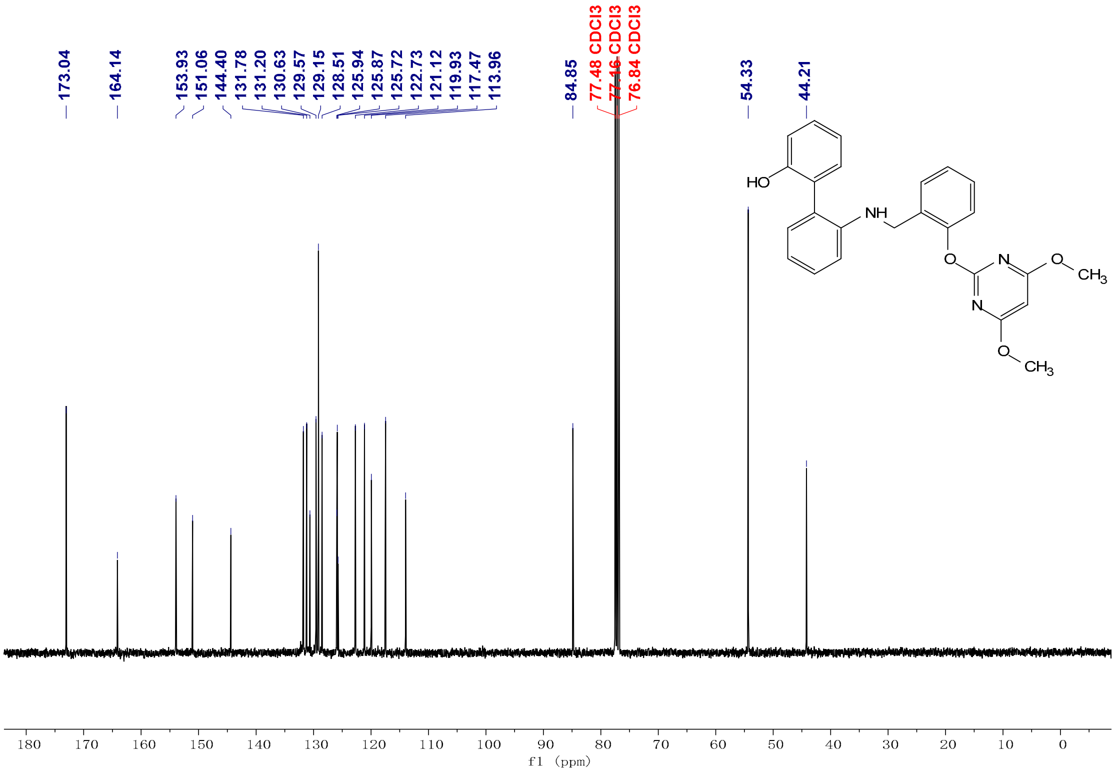

^{1}H-NMR spectrum for **Iec** (400 MHz, $CDCl_3$)

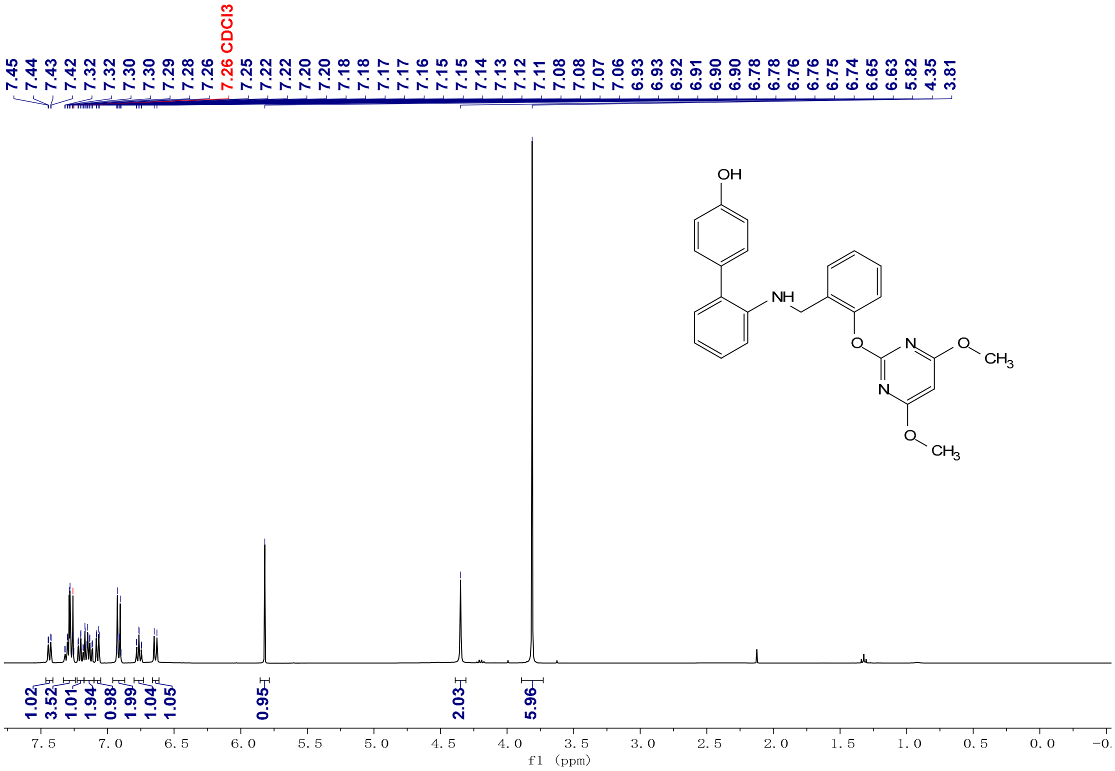

^{13}C-NMR spectrum for **Iec** (101 MHz, $CDCl_3$)

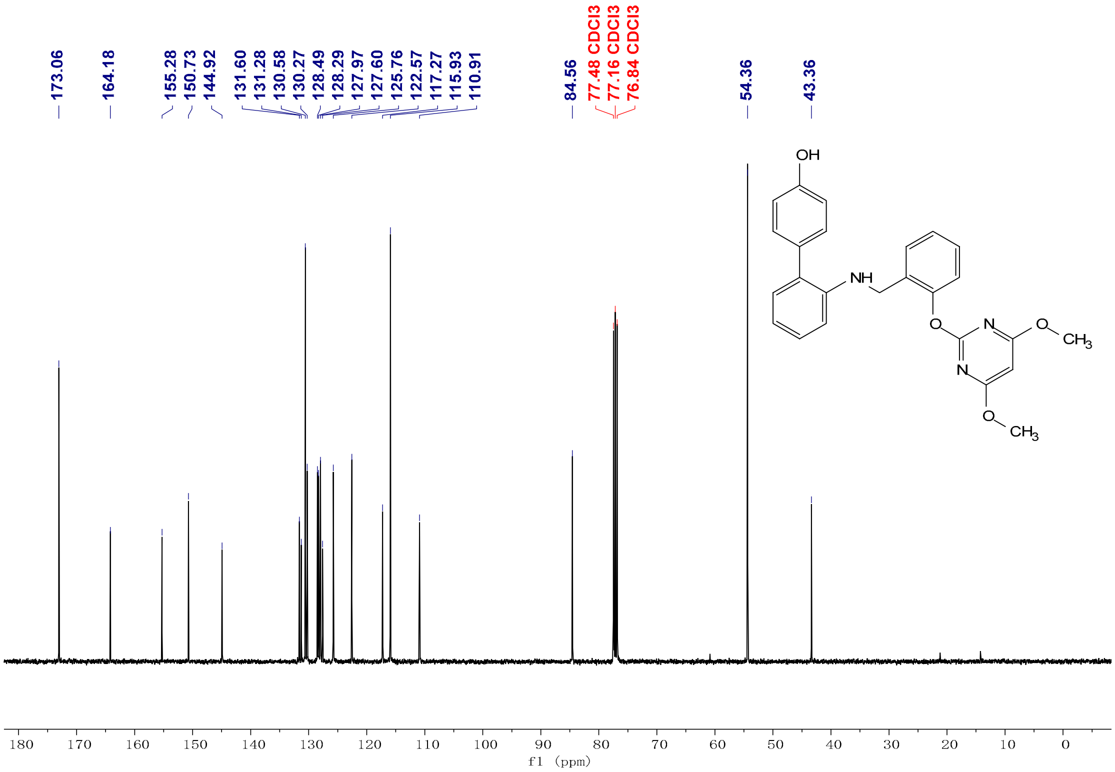

^{1}H-NMR spectrum for **Ifa** (400 MHz, $CDCl_3$)

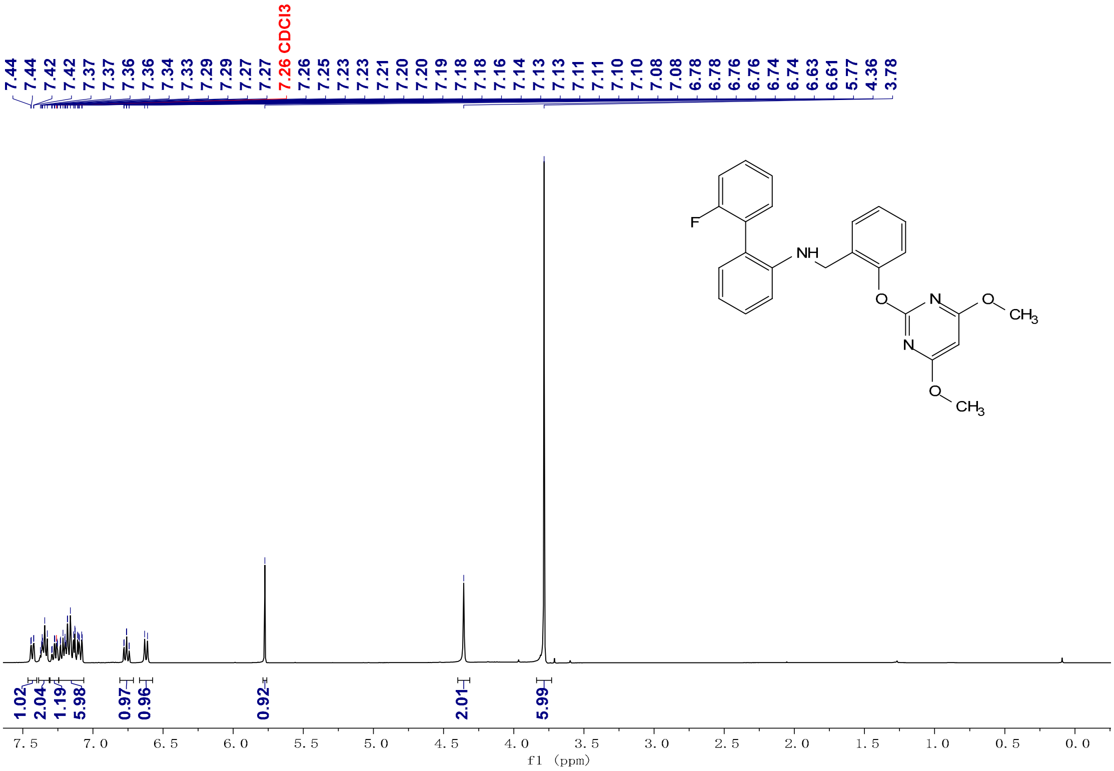

^{13}C-NMR spectrum for **Ifa** (101 MHz, $CDCl_3$)

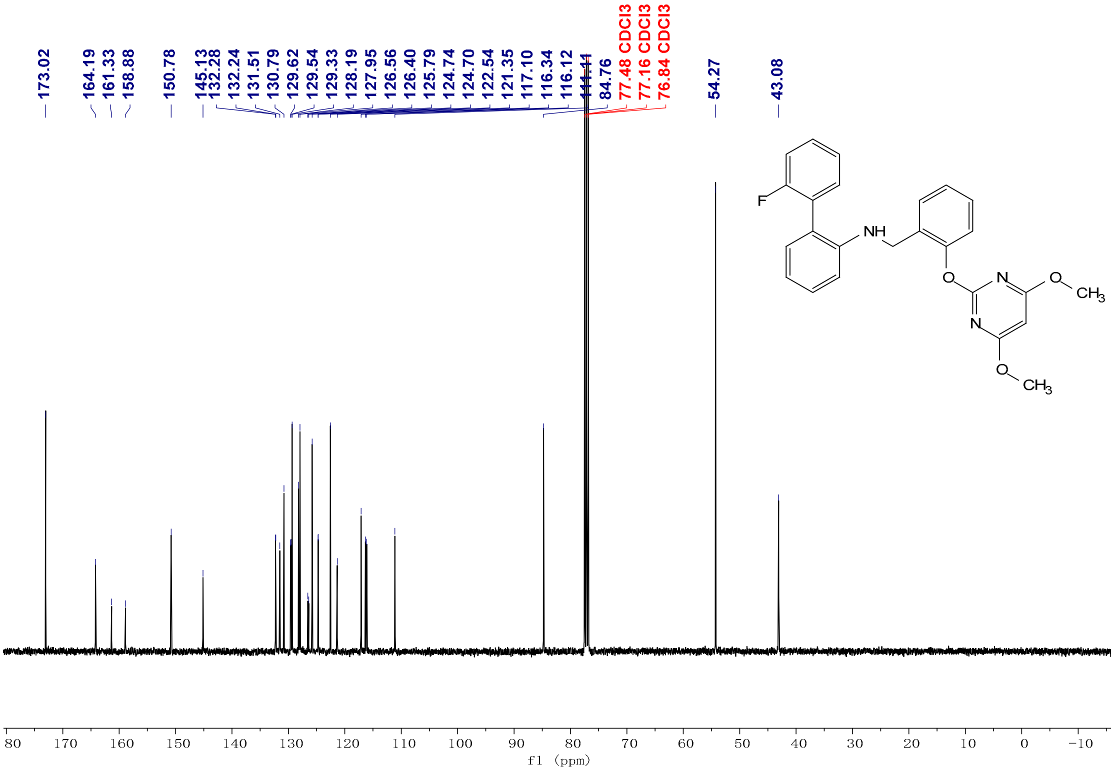

^{19}F-NMR spectrum for **Ifa** (376 MHz, $CDCl_3$)

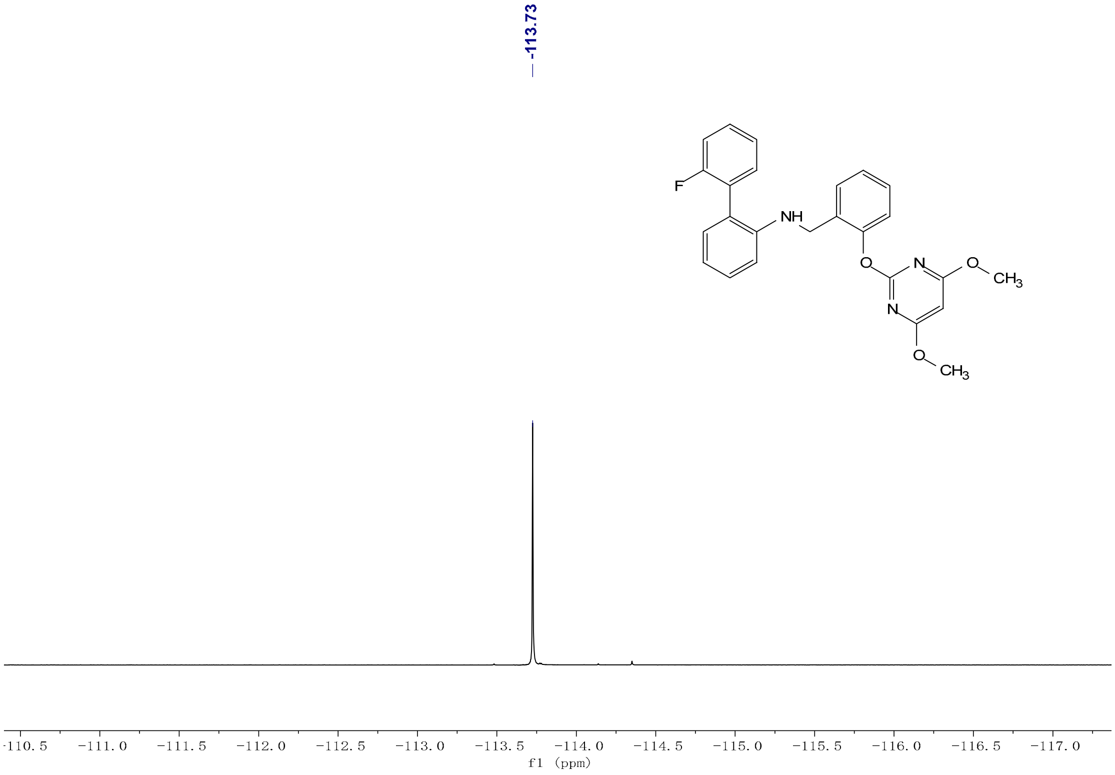

^{1}H-NMR spectrum for **Ifc** (400 MHz, $CDCl_3$)

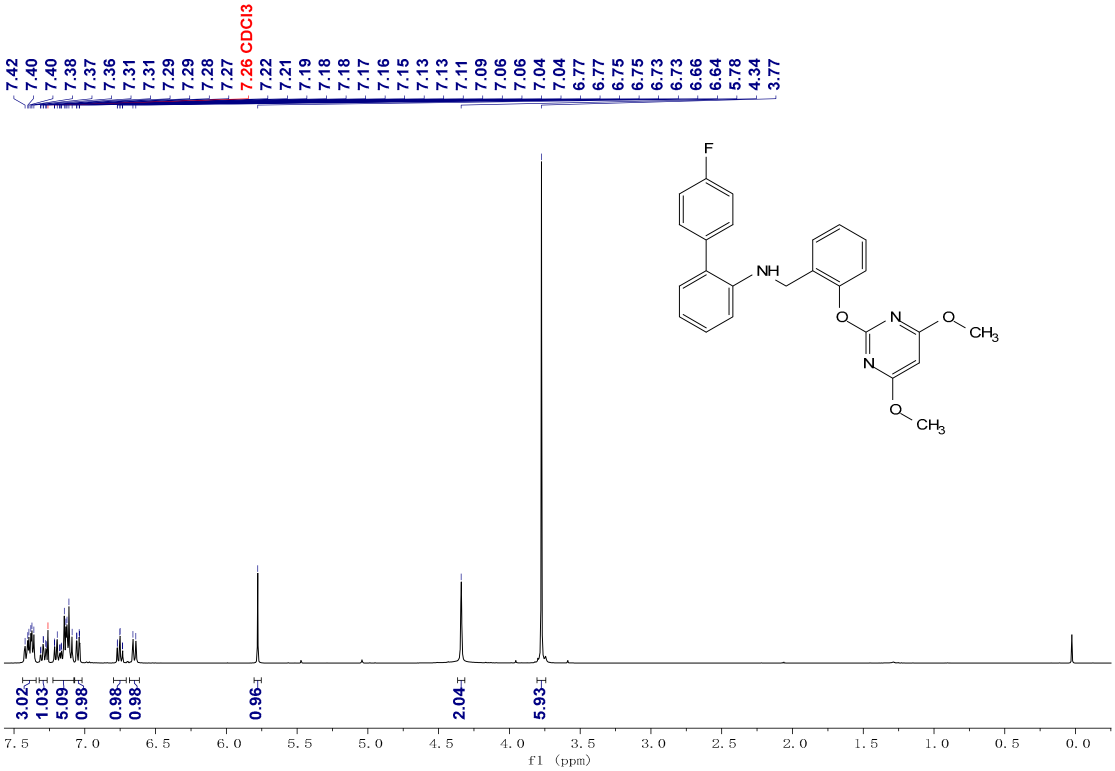

^{13}C-NMR spectrum for **Iec** (101 MHz, $CDCl_3$)

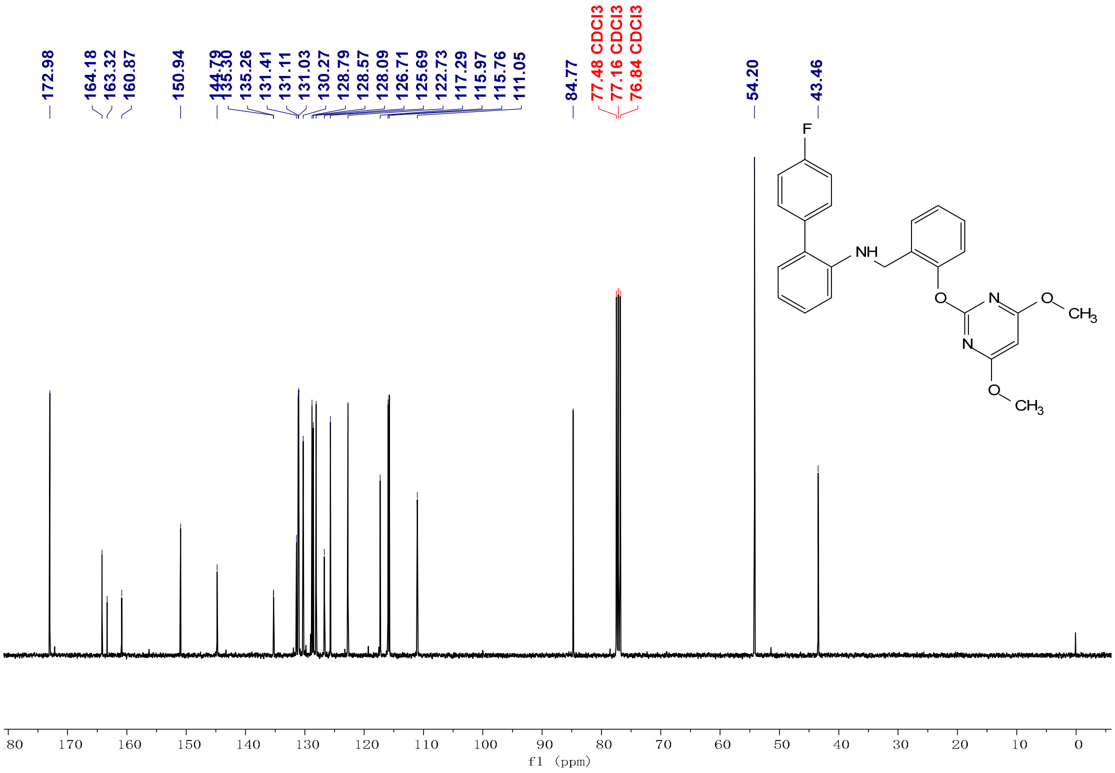

^{19}F-NMR spectrum for **Iec** (376 MHz, $CDCl_3$)

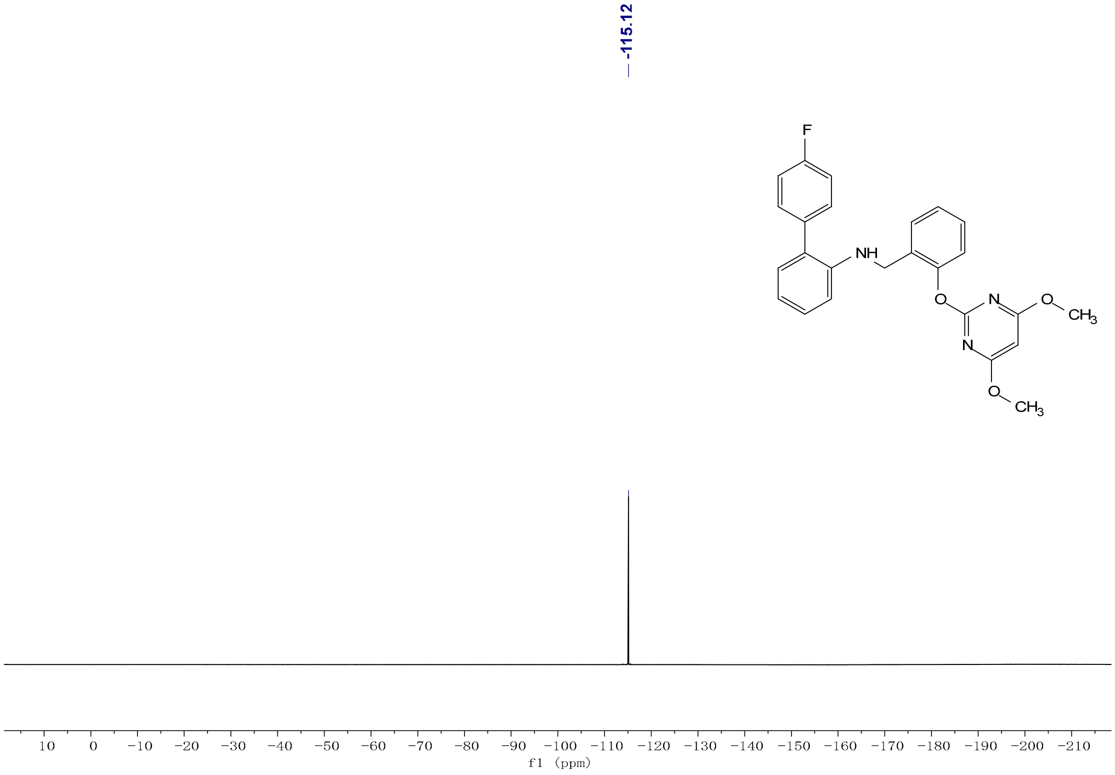

^{1}H-NMR spectrum for **Iga** (400 MHz, $CDCl_3$)

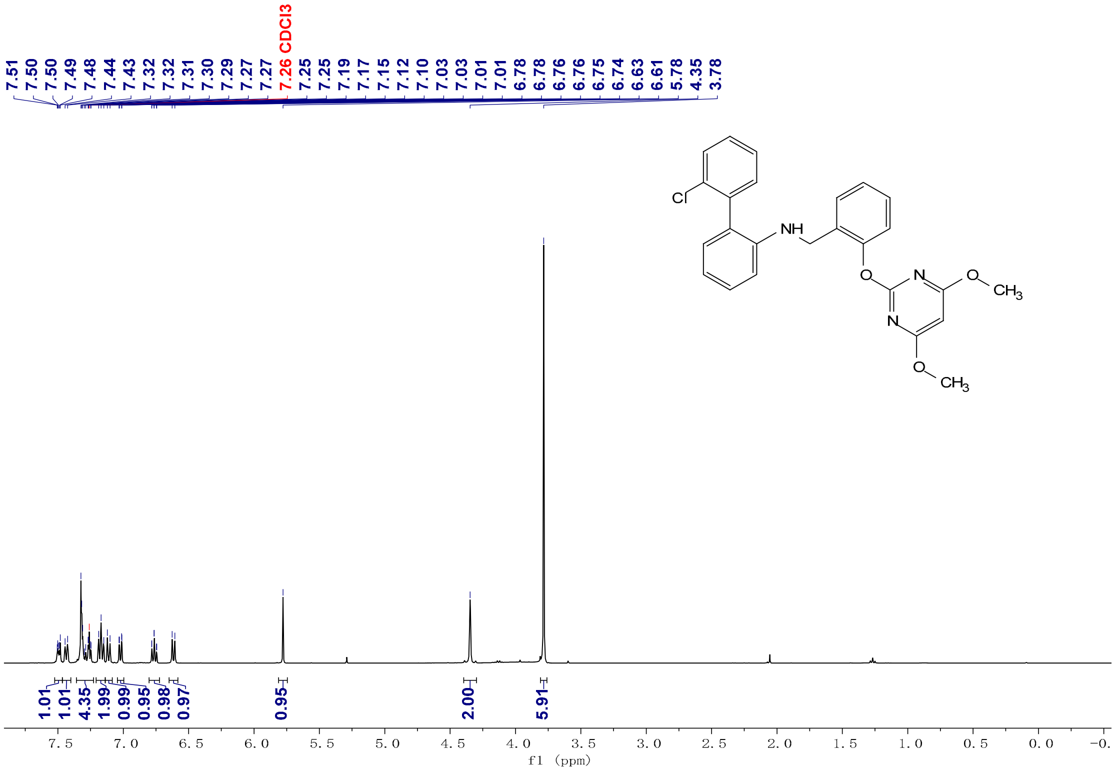

^{13}C-NMR spectrum for **Iga** (101 MHz, $CDCl_3$)

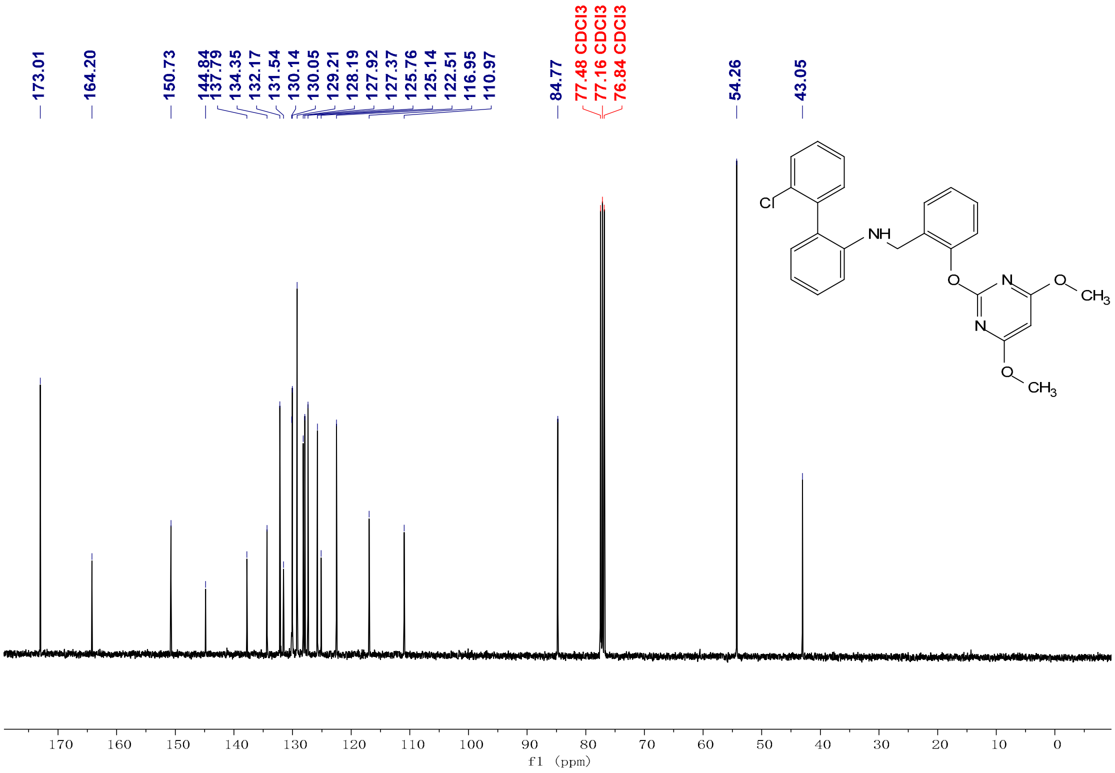

^{1}H-NMR spectrum for **Igc** (400 MHz, $CDCl_3$)

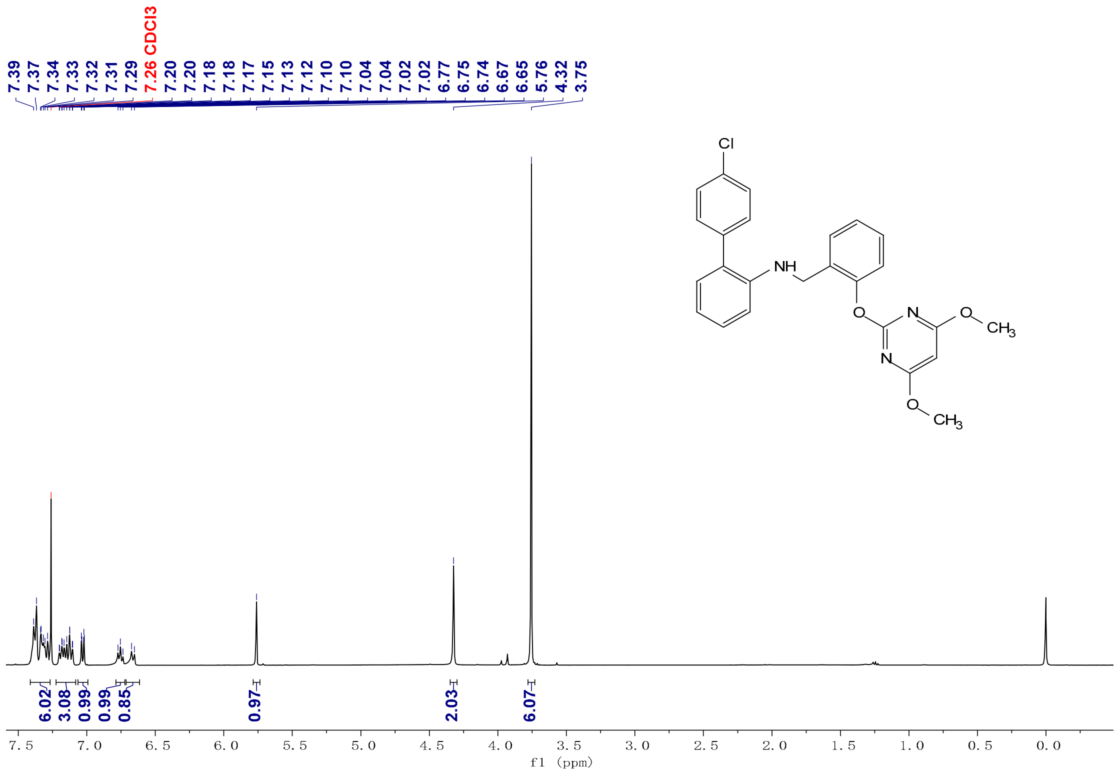

^{13}C-NMR spectrum for **Igc** (101 MHz, $CDCl_3$)

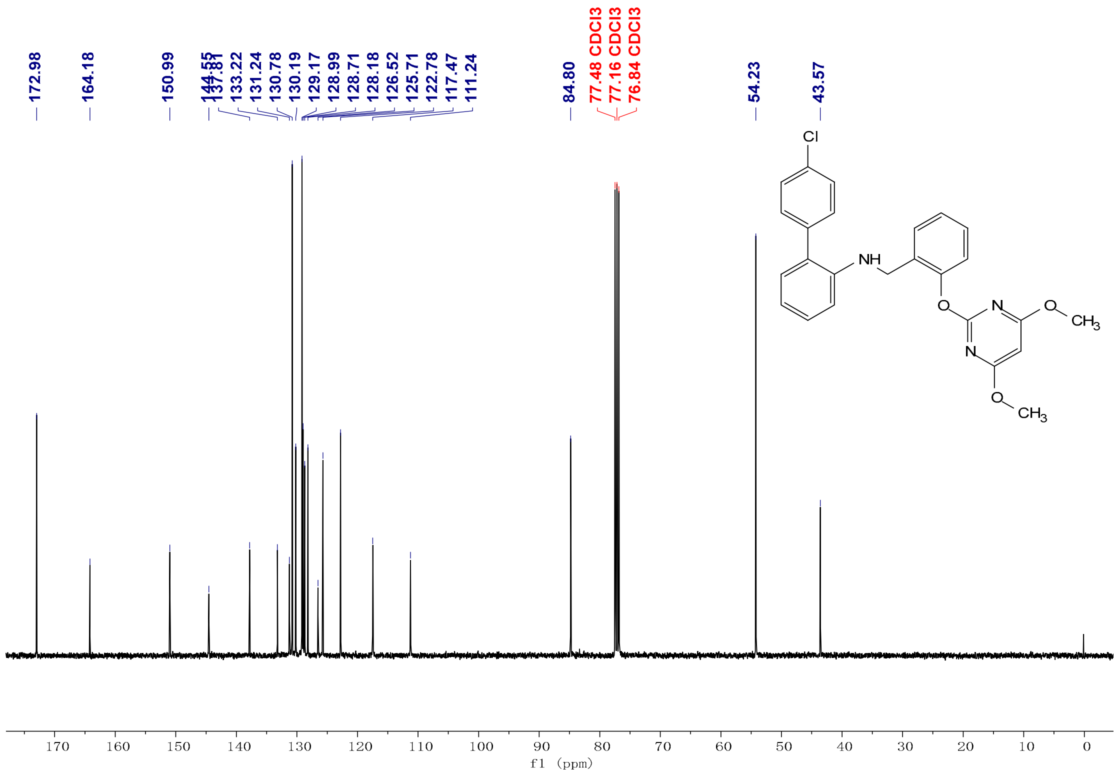

^{1}H-NMR spectrum for **Iha** (400 MHz, $CDCl_3$)

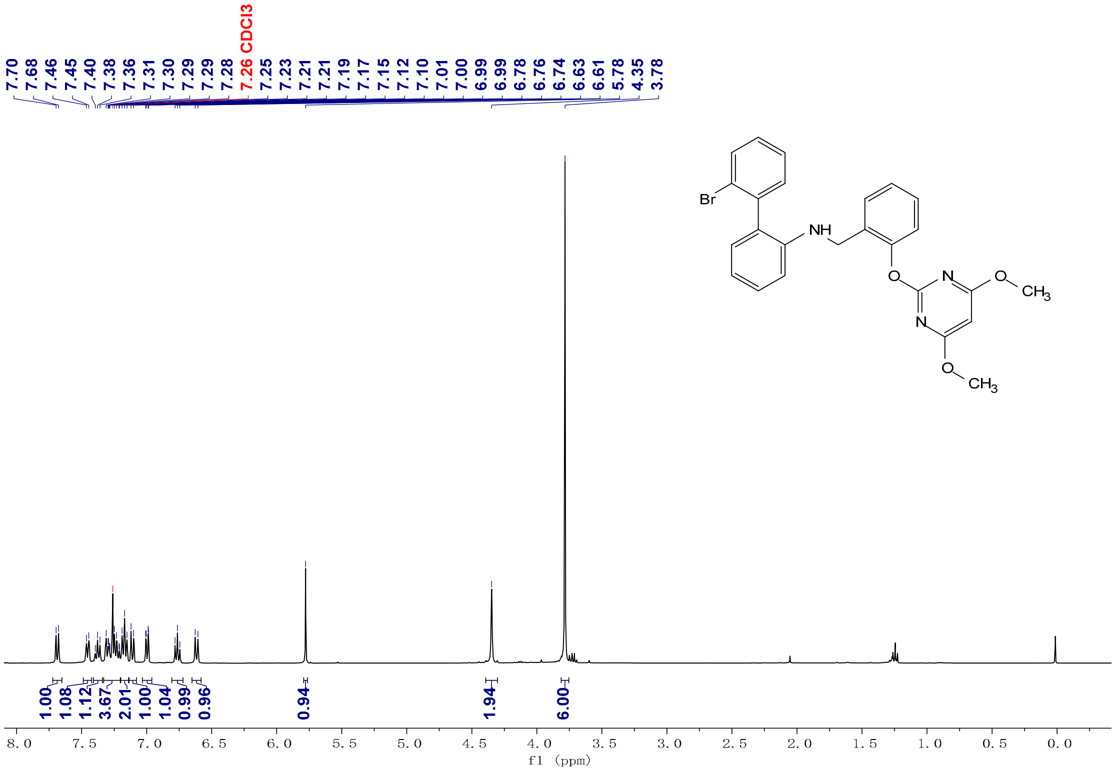

^{13}C-NMR spectrum for **Iha** (101 MHz, $CDCl_3$)

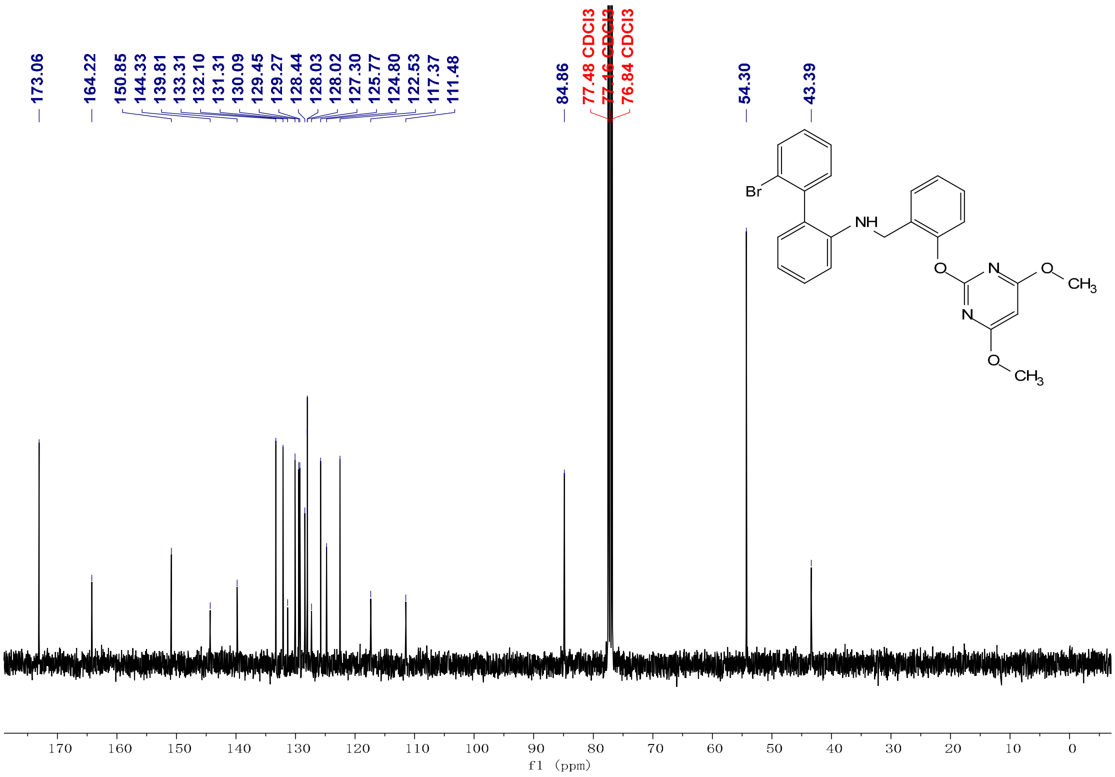

^{1}H-NMR spectrum for **Iia** (400 MHz, $CDCl_3$)

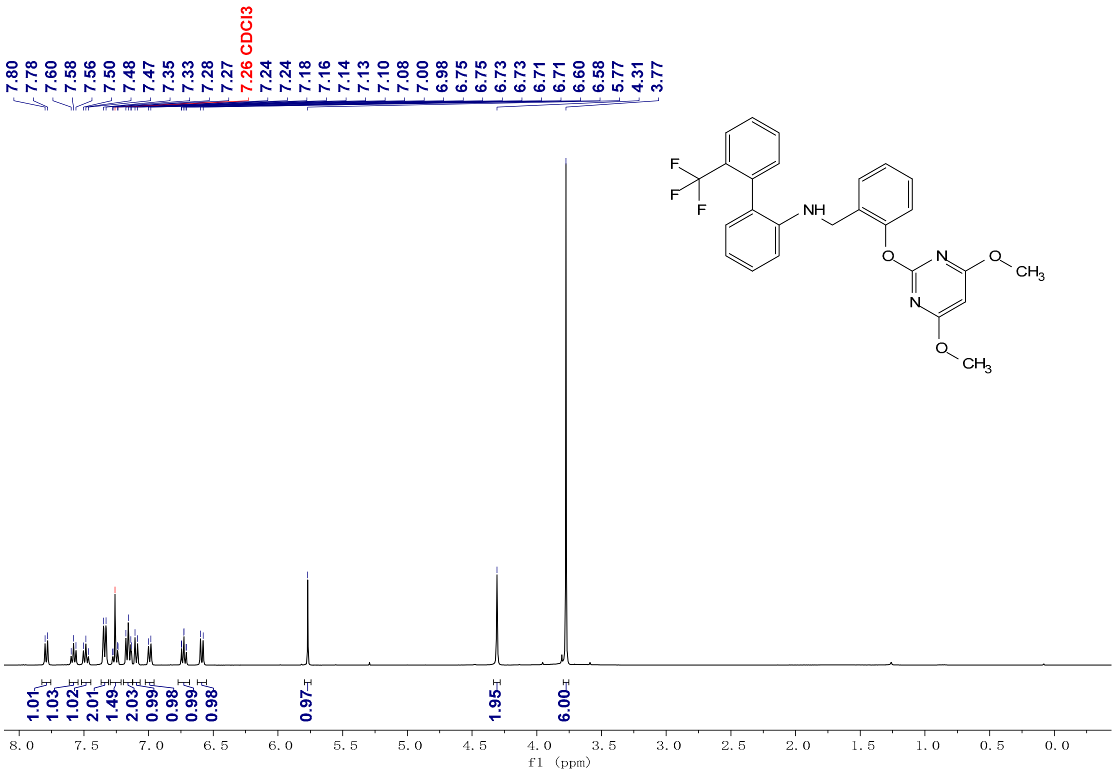

^{13}C-NMR spectrum for **Iia** (101 MHz, $CDCl_3$)

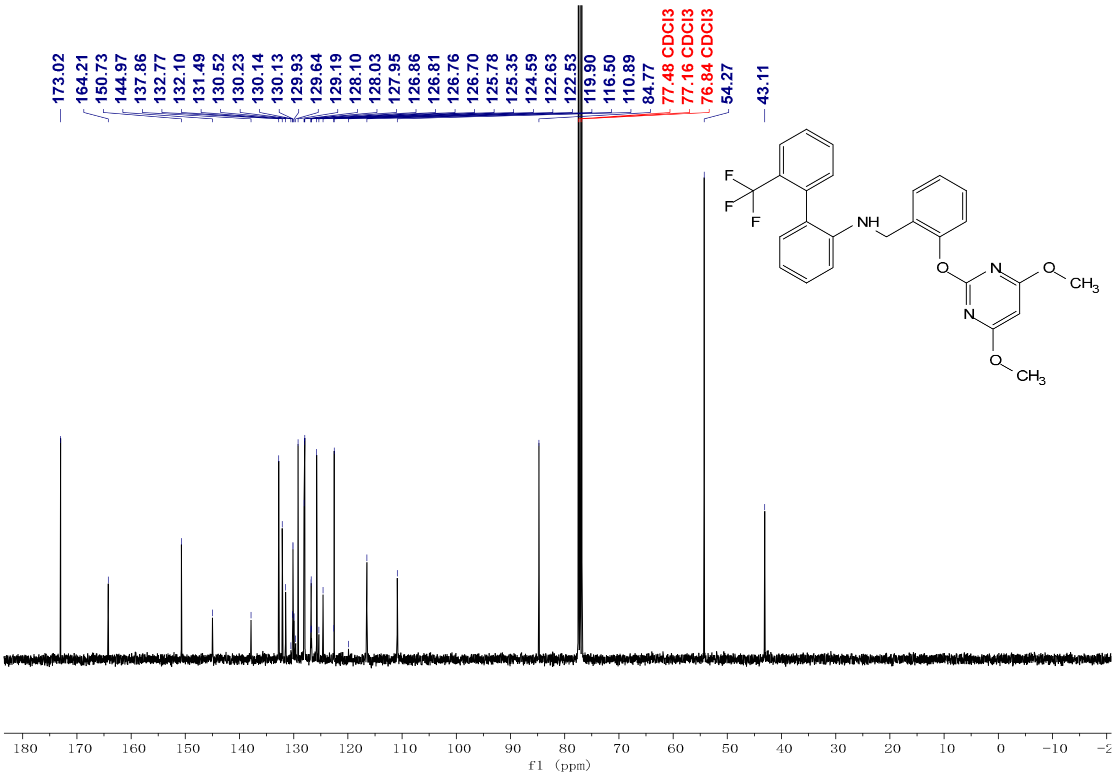

^{19}F-NMR spectrum for **Iia** (376 MHz, $CDCl_3$)

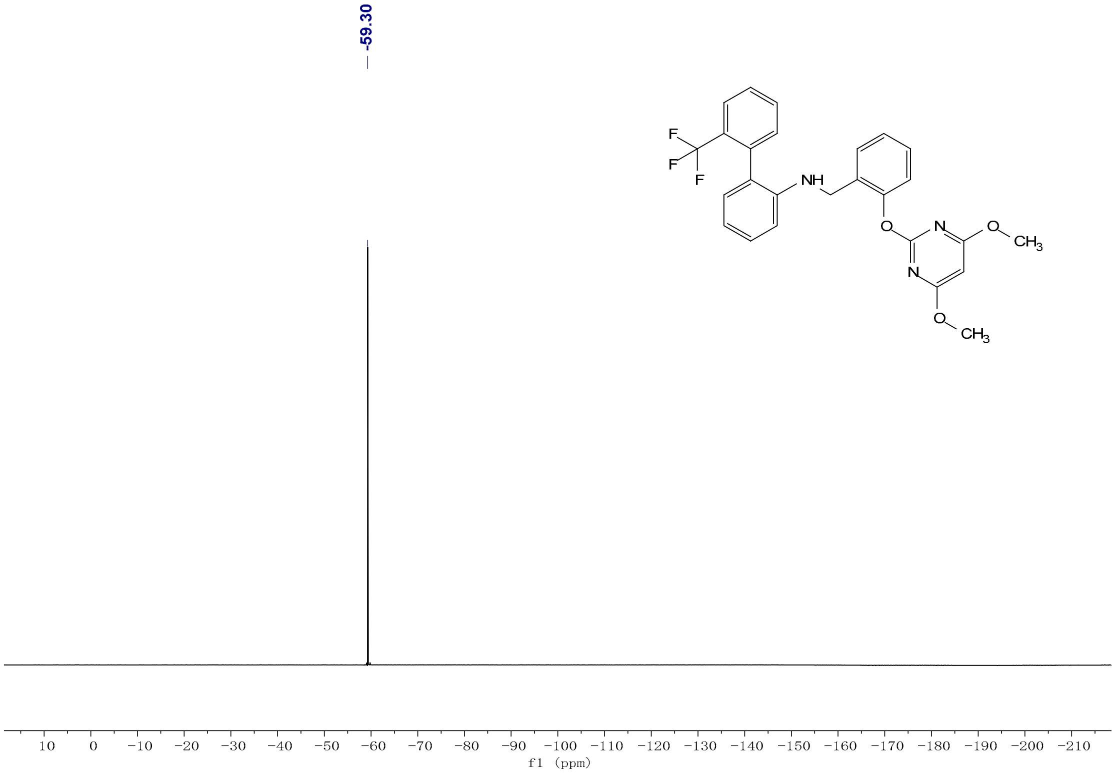

^{1}H-NMR spectrum for **Iic** (400 MHz, $CDCl_3$)

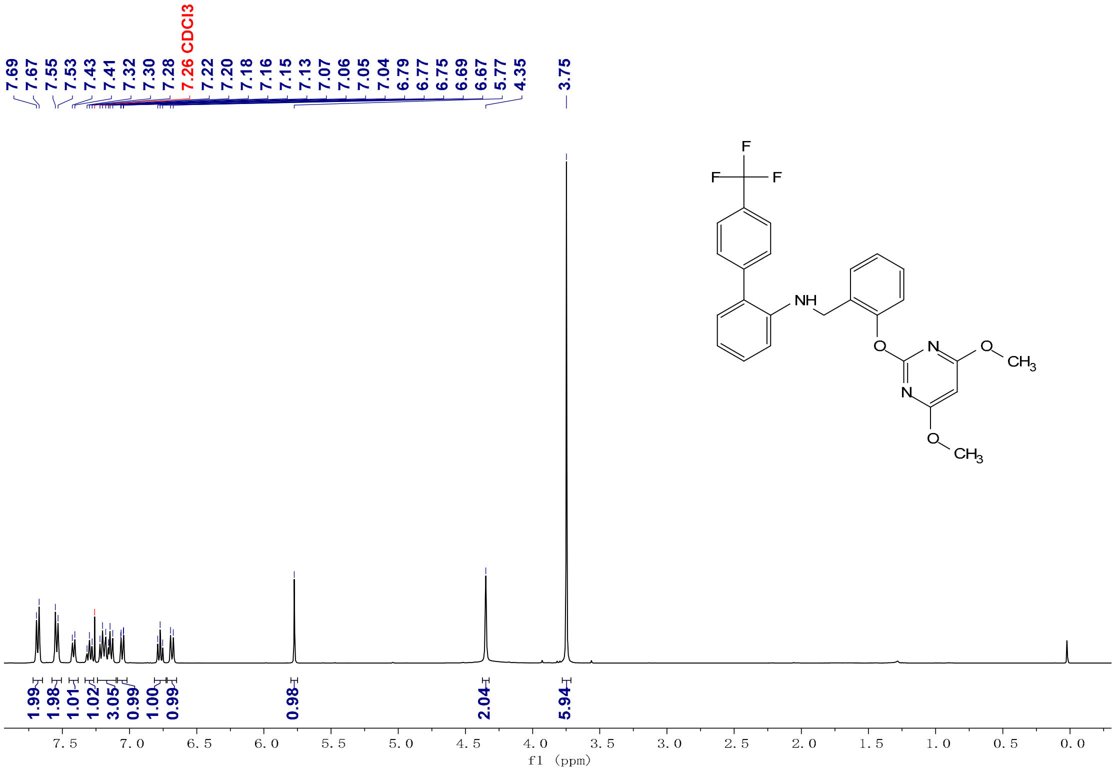

^{13}C-NMR spectrum for **Iic** (101 MHz, $CDCl_3$)

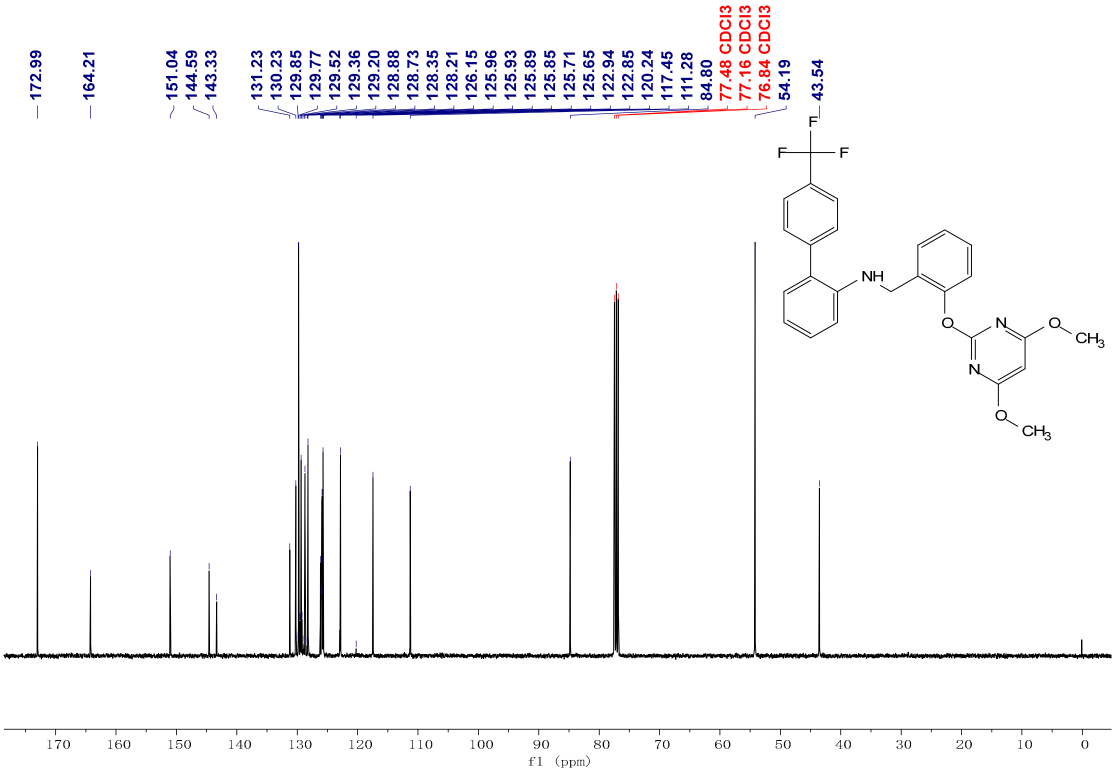

^{19}F-NMR spectrum for **Iic** (376 MHz, $CDCl_3$)

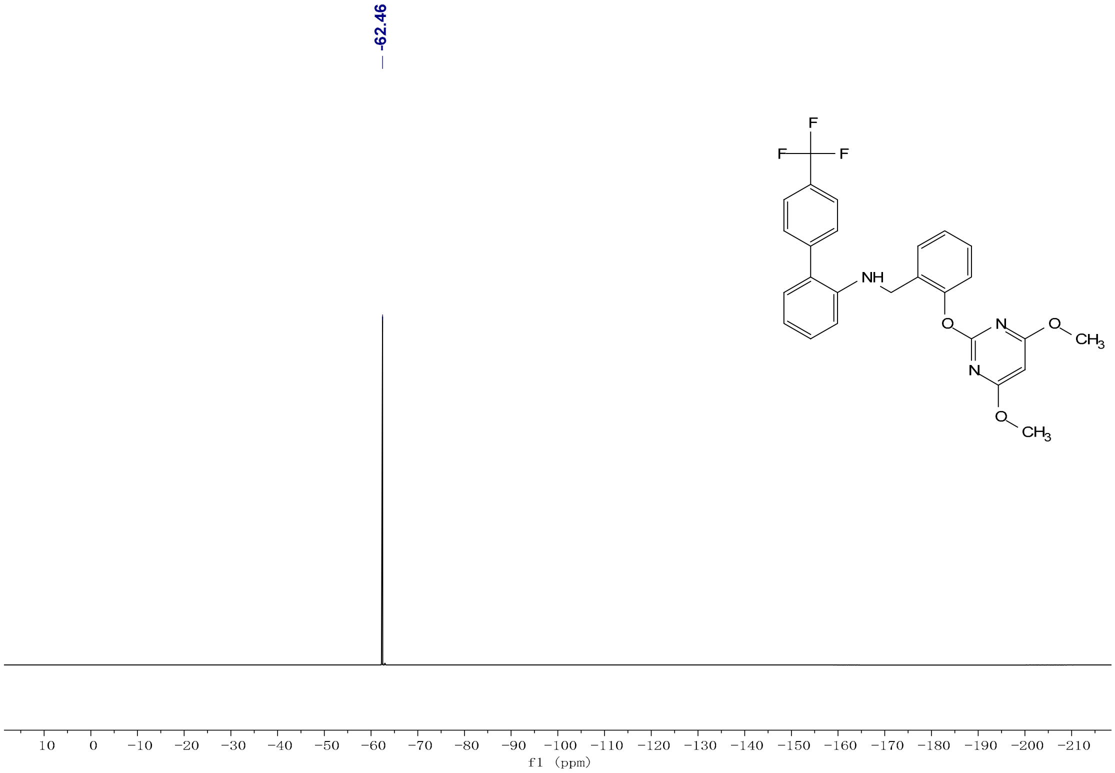

Supplement: Supplementary file 1 [file molecules-29-02409-s001.zip › molecules-3001194-supplementary.pdf]
